# An empirical assessment of a single family‐wide hybrid capture locus set at multiple evolutionary timescales in Asteraceae

**DOI:** 10.1002/aps3.11295

**Published:** 2019-10-25

**Authors:** Katy E. Jones, Tomáš Fér, Roswitha E. Schmickl, Rebecca B. Dikow, Vicki A. Funk, Sonia Herrando‐Moraira, Paul R. Johnston, Norbert Kilian, Carolina M. Siniscalchi, Alfonso Susanna, Marek Slovák, Ramhari Thapa, Linda E. Watson, Jennifer R. Mandel

**Affiliations:** ^1^ Botanischer Garten und Botanisches Museum Berlin Freie Universität Berlin Königin‐Luise‐Str. 6–8 14195 Berlin Germany; ^2^ Department of Botany Faculty of Science Charles University Benátská 2 CZ 12800 Prague Czech Republic; ^3^ Institute of Botany The Czech Academy of Sciences Zámek 1 CZ 25243 Průhonice Czech Republic; ^4^ Data Science Lab Office of the Chief Information Officer Smithsonian Institution Washington D.C. 20013‐7012 USA; ^5^ Department of Botany National Museum of Natural History Smithsonian Institution Washington D.C. 20013‐7012 USA; ^6^ Botanic Institute of Barcelona (IBB‐CSIC‐ICUB) Pg. del Migdia s.n. ES 08038 Barcelona Spain; ^7^ Freie Universität Berlin Evolutionary Biology Berlin Germany; ^8^ Berlin Center for Genomics in Biodiversity Research Berlin Germany; ^9^ Leibniz‐Institute of Freshwater Ecology and Inland Fisheries (IGB) Berlin Germany; ^10^ Department of Biological Sciences University of Memphis Memphis Tennessee 38152 USA; ^11^ Center for Biodiversity University of Memphis Memphis Tennessee 38152 USA; ^12^ Plant Science and Biodiversity Centre Slovak Academy of Sciences SK‐84523 Bratislava Slovakia; ^13^ Department of Plant Biology, Ecology, and Evolution Oklahoma State University Stillwater Oklahoma 74078 USA

**Keywords:** Asteraceae, Compositae, hybrid capture, Hyb‐Seq, non‐paralogy, phylogenetics

## Abstract

**Premise:**

Hybrid capture with high‐throughput sequencing (Hyb‐Seq) is a powerful tool for evolutionary studies. The applicability of an Asteraceae family‐specific Hyb‐Seq probe set and the outcomes of different phylogenetic analyses are investigated here.

**Methods:**

Hyb‐Seq data from 112 Asteraceae samples were organized into groups at different taxonomic levels (tribe, genus, and species). For each group, data sets of non‐paralogous loci were built and proportions of parsimony informative characters estimated. The impacts of analyzing alternative data sets, removing long branches, and type of analysis on tree resolution and inferred topologies were investigated in tribe Cichorieae.

**Results:**

Alignments of the Asteraceae family‐wide Hyb‐Seq locus set were parsimony informative at all taxonomic levels. Levels of resolution and topologies inferred at shallower nodes differed depending on the locus data set and the type of analysis, and were affected by the presence of long branches.

**Discussion:**

The approach used to build a Hyb‐Seq locus data set influenced resolution and topologies inferred in phylogenetic analyses. Removal of long branches improved the reliability of topological inferences in maximum likelihood analyses. The Astereaceae Hyb‐Seq probe set is applicable at multiple taxonomic depths, which demonstrates that probe sets do not necessarily need to be lineage‐specific.

Evolutionary studies at high and low taxonomic levels have frequently been hindered by poor phylogenetic resolution. High‐throughput sequencing (HTS) approaches enable biologists to sample a larger portion of the genome compared to traditional Sanger sequencing, and it is now possible to robustly test a range of phylogenetic hypotheses. However, phenomena such as whole genome duplications (WGDs), ancestral and recent hybridization, and rapid radiations remain a challenge even when using HTS data (Straub et al., 2014; Tiley et al., 2016). Asteraceae, the largest flowering plant family (10–12% of all flowering plants, 25,000–33,000 species; Mandel et al., 2017, 2019), serves as a good example for the aforementioned challenges (Fig. 1). Since its origin in the Late Cretaceous (76–66 mya), the family has undergone multiple rounds of WGDs (Barreda et al., 2015; Huang et al., 2016) and hybridization across various timescales (e.g., within Senecioneae; Pelser et al., 2010). Furthermore, rapid radiations are common in the family; for example, Hawaiian silverswords (Baldwin and Sanderson, 1998), Hawaiian *Bidens* L. (Knope et al., 2012), and tropical Andean *Espeletia* Mutis ex Bonpl. (Diazgranados and Barber, 2017; Pouchon et al., 2018) are among a few well‐studied Asteraceae radiations.

**Figure 1:**
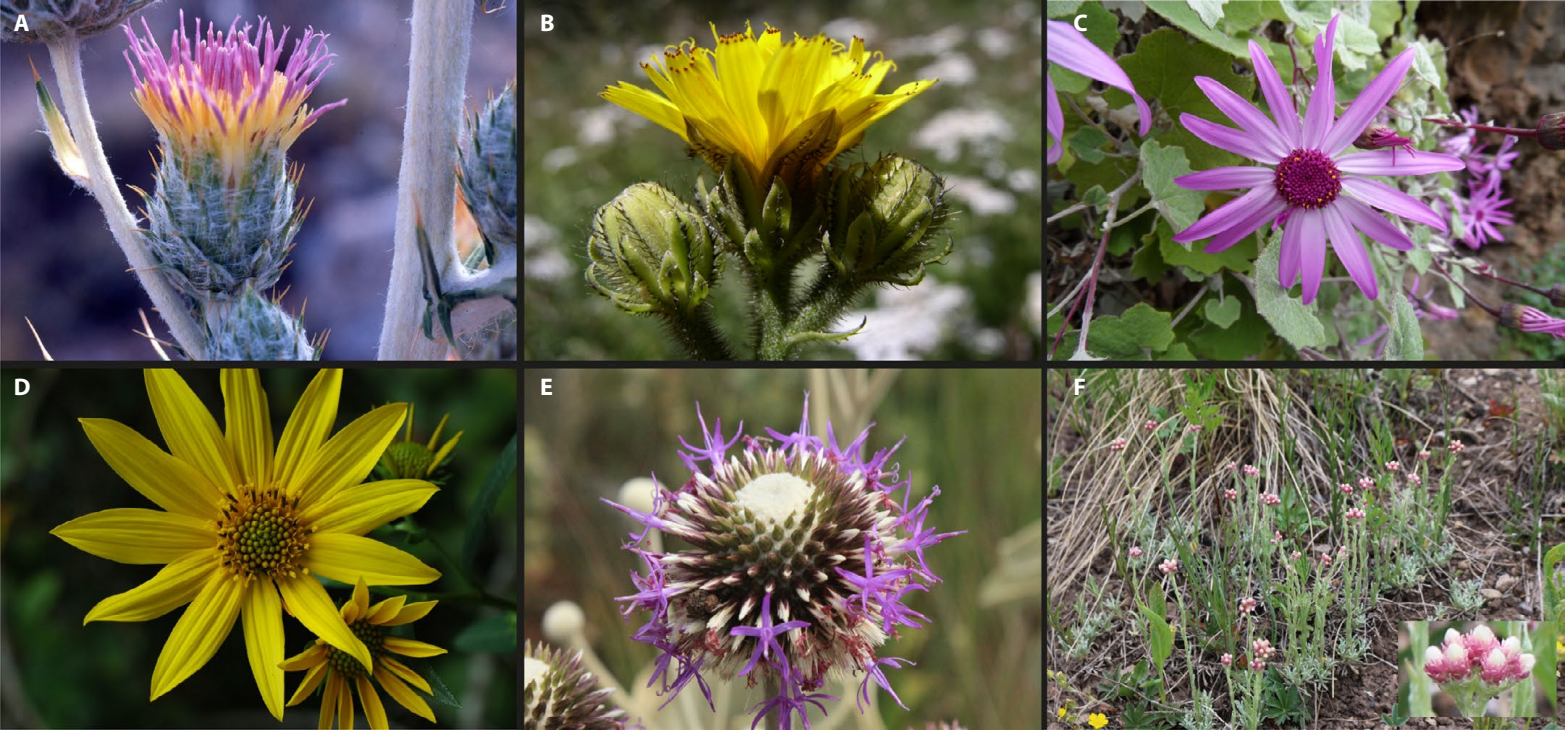
Diversity of Asteraceae shown by representative species from the genera sampled in this study from six tribes across the Asteraceae. For each image, we provide species name (tribe), locality, and (photo by, year taken); where vouchers exist, the collector name, number, and herbarium are also given. (A) *Cousinia lanata* (Cardueae), in Voru, Tajikistan (A. Susanna, 2004), a member of one of the largest genera of the Asteraceae. (B) *Picris hieracioides* subsp. *umbellata* (Cichorieae) growing in Soldeu village in the Pyrenean mountains, Andorra (M. Slovák, 2004), a member of the *P. hieracioides* species complex that shows differences in topological inferences depending on the COS locus subset and phylogenetic analyses. (C) *Pericallis lanata* (Senecioneae) growing on steep slopes in Guía de Isora, Barranco Tagara, Tenerife, Canary Islands, Spain (K. E. Jones, 2011); voucher: *K. E. Jones and A. Reyes‐Betancort 231* (BM). (D) *Helianthus verticillatus* (Heliantheae), growing in Georgia, USA (Christopher Brown, 2006). (E) *Chresta sphaerocephala* (Vernonieae), growing in Parque Nacional Serra da Canastra Minas Gerais, São Roque de Minas, Brazil (C. M. Siniscalchi, 2014); voucher: *C. M. Siniscalchi 444* (SPF). (F) *Antennaria rosea* (Gnaphalieae) in Carson National Forest, Rio Arriba County, New Mexico, USA (Ram Thapa, 2017); voucher: *R. J. Bayer, R. Thapa, N. P. Prather & S. M. Bollou NM‐17002* (MEM).

Recent studies have estimated family‐level phylogenies for Asteraceae: Fu et al. (2016) and Panero and Crozier (2016) used multi‐locus plastid data, and Huang et al. (2016) used HTS to obtain 175 orthologous nuclear markers from transcriptome data. Following Funk et al.'s (2009) Asteraceae family‐wide supertree approach and phylogenetic studies for different tribes (see parts 2–4 within Funk et al., 2009), the Asteraceae community needed a set of loci that could be used for phylogenetic analyses across the family and, if possible, for multiple taxonomic levels (i.e., family, tribe, genus, species). Therefore, Mandel et al. (2014) published a probe set designed for hybrid capture in combination with genome skimming, using HTS (hereafter Hyb‐Seq; Weitemier et al., 2014), that targets 1061 nuclear loci that are potentially low‐copy and orthologous across the Asteraceae family, based on conserved markers (hereafter referred to as the conserved orthologous set [COS]; Mandel et al., 2014). The COS locus set (MyBaits COS Compositae/Asteraceae1kv1; Arbor Biosciences, Ann Arbor, Michigan, USA) has been demonstrated to provide a well‐resolved family backbone, with high resolution at the subfamily and tribal levels (Mandel et al., 2017, 2019). The locus set has also helped to disentangle relationships among complex and diverse genera within tribe Cardueae (Herrando‐Moraira et al., 2018, 2019). Robust divergence time estimations across the family can now be performed (Mandel et al., 2019). However, there is a need for a critical assessment of the applicability of the Asteraceae COS locus set across multiple taxonomic levels (i.e., tribes, genera, species), including tests for the proportions of parsimony informative (PI) loci.

Probe design for Hyb‐Seq may be lineage‐specific, most often at the genus level (e.g., *Bartsia* L. [Uribe‐Convers et al., 2016], *Heuchera* L. [Folk et al., 2015], *Inga* Mill. [Nicholls et al., 2015], *Sarracenia* L. [Stephens et al., 2015], *Oxalis* L. [Schmickl et al., 2015], and *Sabal* Adans. [Heyduk et al., 2015]). Such a lineage‐specific design may also work at higher taxonomic levels (e.g., families Annonaceae [Couvreur et al., 2019], Arecaceae [de La Harpe et al., 2019], and Fabaceae [Vatanparast et al., 2018] and order Zingiberales [Carlsen et al., 2018]) and even at lower taxonomic levels (e.g., at the population‐level in *Euphorbia balsamifera* Aiton using a genus‐level probe set for *Euphorbia* L. [Villaverde et al., 2018]). In contrast to lineage‐specific probe sets, a universal angiosperm‐wide set for anchored hybrid enrichment of ~400 loci has been developed (Buddenhagen et al., 2016) that has been successfully applied to a number of studies, for example for *Aristolochia* L. (Wanke et al., 2017) and *Protea* L. (Mitchell et al., 2017). More recently, a universal kit for Hyb‐Seq has also become available that is parsimony informative at the infrageneric level across potentially all angiosperm families, including Linaceae, Onagraceae, Portulacaceae, and Poaceae (Johnson et al., 2019), as well as *Nepenthes* L. (Murphy et al., 2019). Studies on *Erica* L. (Kadlec et al., 2017) and Buddlejeae (Chau et al., 2017) suggest that a lineage‐specific probe design strategy provides more markers that are phylogenetically informative at lower taxonomic levels, compared to universal probe sets. However, Liu et al. (2019) showed that good target enrichment is possible when probe sets are <30% divergent from the target regions. Johnson et al. (2019) took this threshold into account when designing the angiosperm‐wide 353 probe set. Therefore, universal probe sets have the potential to be just as informative as lineage‐specific probe sets at lower taxonomic levels, as long as sufficient probes are included to account for the diversity they encompass and they account for the <30% threshold between probes and target regions. Furthermore, universal probe sets have the advantage of enabling comparable hybrid capture efficiency for both ingroup and outgroup taxa, which is particularly important if one aims to conduct divergence time estimates. The Asteraceae COS probe set can be considered both lineage‐specific (Asteraceae) and universal (the loci have been designed to work across this enormous family, not for a small lineage). The COS probe set also includes 1061 target loci; therefore, there is good potential to capture the diversity and build large multi‐locus data sets at multiple taxonomic depths. This provides an opportunity to empirically assess its applicability as a non‐paralogous and phylogenetically informative locus set for multiple taxonomic levels (i.e., tribe, genus, species) and therefore different evolutionary timescales. Furthermore, because more universal probe sets are becoming available at even broader phylogenetic scales across land plants, not only for flowering plants (e.g., Buddenhagen et al., 2016; Johnson et al., 2019), but also for flagellate plants such as mosses (Liu et al., 2019) and ferns (Wolf et al., 2018), the results of this study should be relevant for anyone wishing to undertake a Hyb‐Seq approach.

When the optimal probe set for Hyb‐Seq has been selected for a study group, whether lineage‐specific or universal, phylogenetic resolution largely depends on the sampling of loci. For lineages that have undergone rampant WGDs, like in Asteraceae, it is recommended to restrict analyses to loci that are non‐paralogous across the study group (Mandel et al., 2015, 2017). However, studies have shown that potentially paralogous loci can be informative in phylogenetic studies of *Artocarpus* J. R. Forst. & G. Forst. (Gardner et al., 2016; Johnson et al., 2016). Furthermore, under the multispecies coalescent (MSC) model, species tree inference with paralogous loci can be accurate (Du et al., 2019a). In some studies, loci that are potentially paralogous, in one or more samples, are removed from the entire data set prior to tree estimations (e.g., Crowl et al., 2017). When potential paralogs are removed and taxonomic sampling is broad, loci that are phylogenetically informative for clades at lower taxonomic levels might get removed if they are paralogous in only a few members of more distantly related clades. In a large phylogeny, this may negatively affect resolution or influence the topologies inferred for some clades. The taxon composition of the sample group under investigation would likely influence which loci are flagged as paralogous. Increasingly larger sets of loci are becoming available for phylogenomic studies; however, little investigation has focused on testing the strategies for locus sampling. Edwards (2016) highlighted the significance of “phylogenomic subsampling,” whereby loci are sampled at random from a large data set from HTS to build different matrices (i.e., subsets) for phylogenetic analyses and to test for consistency between the analyses of different locus subsets. Other studies have illustrated the power of the ordered addition of loci to increasingly larger matrices for phylogenetic analyses (Simon et al., 2012; Bayzid and Warnow, 2013). However, Adams and Castoe (2019) recently showed that statistical gene tree binning, an approach that attempts to avoid gene tree error, can in fact lead to further exacerbation of gene tree error (Adams and Castoe, 2019). As an additional approach to phylogenomic data subsampling, we explore the impact of a “guided” locus subsampling strategy to build alternative data sets, based on the identification of non‐paralogous loci at different taxonomic levels (tribe to species), on levels of PI sites. Therefore, this study tests how different data sets of the Asteraceae COS locus set built for different taxonomic levels may influence resolution and topological inference in phylogenetic reconstructions in Cichorieae.

In addition to the strategy used to build the locus data set, phylogenetic resolution may be influenced by the method used to generate the phylogenetic hypothesis. A widely used phylogenetic method is concatenation analysis with maximum likelihood (ML), which involves combining all locus alignments into a supermatrix and using an ML method such as randomized axelerated maximum likelihood (RAxML; Stamatakis, 2006). Biological processes such as hybridization and incomplete lineage sorting can cause gene trees estimated from different loci to differ from the overall species tree and lead to discordance among gene trees. Incomplete lineage sorting occurs when genes from two taxa fail to coalesce in the most recent ancestor (Chou et al., 2015). Thus, a supermatrix approach may be statistically inconsistent under the MSC model and can result in a tree that does not reflect the species tree (Chou et al., 2015). As well as biological processes, methodological artifacts create obstacles for phylogenetic reconstruction and can cause inaccurate gene tree estimations (Qu et al., 2017). Examples of such artifacts include alignment issues and homology errors, such as unrecognized paralogy (Gatesy et al., 2019) and long‐branch attraction, whereby long branches are erroneously grouped together in estimated trees (Felsenstein, 1978; Sanderson et al., 2000; Parks and Goldman, 2014; Qu et al., 2017; Mai and Mirarab, 2018). A number of approaches can help to improve the reliability of concatenation analyses, for example, the use of partitioning and best‐fit substitution models (Xi et al., 2012; Kainer and Lanfear, 2015; Lanfear et al., 2016), elimination of fast‐evolving sites, removal of long branches, or increasing taxon sampling; the latter approach is often challenging due to rare taxa or unknown extinction events (Pisani, 2004; Bergsten, 2005; Qu et al., 2017). The recently developed software TreeShrink can detect (and remove) outlier long branches among gene trees, which can help to alleviate the impact of long‐branch attraction on gene and species tree reconstruction (Mai and Mirarab, 2018). Methods have been developed to estimate species trees in the presence of incomplete lineage sorting under the MSC model; these may be performed using gene tree summary methods (e.g., NJst [Liu and Yu, 2011], SVDquartets [Chifman and Kubatko, 2014], and ASTRAL [Mirarab and Warnow, 2015]). Incomplete lineage sorting, hybridization, and gene duplication processes may even be untangled all at once (Sousa et al., 2017). This approach, however, requires genomic location information, which is not available for most non‐model species. Alternatively, the following approaches do not require genomic location information: guenomu, a Bayesian hierarchical model that estimates species trees from unrooted gene trees from multiple gene families (de Oliveira Martins and Posada, 2017), and a recent model within PhyloNet that incorporates incomplete lineage sorting and gene duplication and loss (Du et al., 2019b). Conflict analyses allow further investigation into discordance between gene and species trees and detection of outlier gene trees for large genomic data sets, for example, using the software phyparts (Smith et al., 2015), which has been used for conflict analyses in a number of lineages, including Pleurothallidinae (Orchidaceae; Bogarín et al., 2018), Portulacineae (Wang et al., 2019), Caryophyllales (Walker et al., 2018), and Metazoa (Shen et al., 2017). At lower taxonomic levels, network approaches might supersede tree‐based approaches due to the large extent of reticulation in such data sets. Tribe Cichorieae, one of the largest tribes in the Asteraceae (>1500 species; Kilian et al., 2009, 2009), is used in this study as a model to test how phylogenetic analyses using different data sets of the Asteraceae COS loci at different taxonomic levels may influence resolution and inferred topologies. Furthermore, we investigate the impact of using different approaches (e.g., ML, ASTRAL, and networks), as well as the influence of removing long branches, on resolution and topologies inferred within Cichorieae.

Finally, little is known about the factors that may influence the number of reads mapping to targets and off‐target regions, and wet‐laboratory procedures during Hyb‐Seq are not always reported in studies where this technique is used (but see Hart et al., 2016; Johnson et al., 2019; and Villaverde et al., 2018). Because the same COS locus set is used for Hyb‐Seq in this study across a wide range of taxa within the Asteraceae family, we explore the influence of combinations of lab steps on the number of reads mapped to targets and off‐target regions (i.e., the plastome).

## Aims

This study represents one of the first assessments of the applicability of a Hyb‐Seq locus set and the impact of different phylogenetic analyses across a wide taxonomic range of plants. The specific aims are to (i) test the suitability of the COS locus set for analyses at a range of taxonomic levels in Asteraceae (seven sample groups at tribe level, 10 at generic level, and four at species complex or species level; Table 1). The broad sampling across the Asteraceae in the present study (Fig. 1, Table 1) enables us to assess the proportions of phylogenetically informative loci for different data sets built for each of the taxonomic levels across a much wider range of tribes and genera compared to previous studies. We then (ii) demonstrate the power of the COS locus set for phylogenetic analyses at different taxonomic levels (broad taxon sampling: tribe‐wide vs. shallow taxon sampling: species complex level) in greater detail, utilizing the tribe Cichorieae as a model. Therefore, we investigate how resolution and topological inference are influenced by the specific data set of non‐paralogous loci that are selected according to the taxonomic level. We demonstrate the influence and applicability of different analyses (i.e., species tree, concatenation, networks, data partitioning), and we compare analyses based only on targeted exons and those based on exons with flanking intron regions (the splash‐zone; Weitemier et al., 2014). Furthermore, we investigate the impact of removing long branches on resolution and topology estimation within Cichorieae. In addition, we (iii) explore how different lab approaches may influence the number of reads mapped to targets and the off‐target plastome across the entire set of samples across the family.

**Table 1:** Taxonomic levels of each sample group, sample group names, number of samples, number of paralogous loci flagged by HybPiper across the sample group, and number of non‐paralogous loci.

Taxonomic level^a^	Sample group name	No. of samples^b^	No. of paralogous loci^c^	No. of non‐paralogous loci^d^
Tribe (19)	Vernonieae	26	636	174
Genus	*Lychnophora* Mart.	6	485	482
Genus	*Chresta* Vell.	6	389	432
Tribe (7)	Heliantheae	13	500	238
Genus	*Helianthus* L.	4	348	702
Genus	*Lipochaeta* DC.	3	376	419
Tribe (5)	Cardueae	14	267	465
Genus	*Cousinia* Cass.	5	250	702
Species	*Carlina vulgaris*	6	190	658
Tribe (5)	Senecioneae	16	590	401
Genus	*Pericallis* D. Don	6	476	404
Genus	*Senecio* L.	7	544	306
Tribe (8)	Gnaphalieae	11	477	240
Genus	*Antennaria* Gaertn.	4	424	452
Tribe (9)	Cichorieae	30	721	212
Genus	*Sonchus* L.	4	341	680
Genus	*Lactuca* L.	6	520	524
Species complex	*Picris hieracioides* complex	9 (6 taxa)	376	610
Species	*Hieracium alpinum*	6	370	647
Species	*Picris hieracioides*	5	371	664
Tribe (2)	Moquinieae	2	461	547

a Numbers in parentheses next to tribe represent the number of different genera sampled within that tribe. Refer to Appendix 1 for list of all samples included at the tribal‐level sampling.

b Number of species per group for tribes and genera and number of samples within a species at the species level.

c Total number of paralogous loci for the sample group.

d After missing data accounted for (samples with >70% missing data removed, followed by removal of loci with any missing samples; see pipeline in Fig. 2; see Appendix S7 for numbers of loci removed at each stage of cleaning).

## METHODS

### Sampling and sample groups

A total of 112 samples across the Asteraceae were included (Appendix 1). To test the suitability of the COS locus set at a range of taxonomic depths and to demonstrate the power of the COS locus set at multiple taxonomic levels in greater depth, the samples were grouped according to monophyletic taxa at different taxonomic levels (i.e., tribe, genus, species complex, species; Table 1). Seven tribes were included, the number of species per tribe ranged from two (Moquinieae; a tribe of just two species) to 30 (Cichorieae; a tribe of >1500 species). Sample size for the five remaining tribes ranged from 11 to 26 species (Table 1). Sampling for 10 of the genera included four to seven species (one individual per species); three samples were included for genus *Lipochaeta* DC. The *Picris hieracioides* L. species complex, with *P. amalecitana* (Boiss.) Eig as the outgroup, consisted of nine individuals and six ingroup taxa: *P. olympica* Boiss., *P. japonica* Thunb., *P. nuristanica* Bornm., *P. hieracioides* subsp. *umbellata* (Schrank) Ces., *P. hieracioides* subsp. *hieracioides*, *P. hieracioides* subsp. *hispidissima* (Bartl.) Slovák & Kučera (one sample per taxon, with the exception of the latter two for which there were two samples per taxon; Table 1, Appendix 1). The three species‐level sample groups consisted of six (*Carlina vulgaris* L., Cardueae; *Hieracium alpinum* L., Cichorieae) and five individuals (*P. hieracioides*, Cichorieae) for the same species. To assess how different factors may influence the number of reads mapped to targets and the off‐target plastome, analyses were conducted across the entire data set and are described below under “Variables influencing numbers of reads mapped to targets and off‐target plastomes” (Appendix S1).

### Laboratory methods

Material for genomic DNA extractions from leaves were either from herbarium specimens, silica‐dried material, or fresh leaf material (see Appendix S1 for details for each sample). This study incorporates data generated in three different labs (University of Memphis, Charles University Prague, and Berlin Botanic Garden); therefore, a number of the wet‐lab steps varied among samples. Below we summarize each step and the range of approaches used; see Appendix S1 for details of steps specific to each sample in this study, and refer to Appendix S2 for detailed COS Hyb‐Seq lab workflows in each lab. Different DNA extraction kits were used: DNeasy Plant Mini Kit (QIAGEN, Hilden, Germany), NucleoSpin Plant II (Macherey‐Nagel GmbH, Düren, Germany), E.Z.N.A. SQ Plant DNA Kit (Omega Bio‐Tek, Norcross, Georgia, USA), Invisorb Spin Plant MiniKit (Invitek Molecular GmbH, Berlin, Germany), and cetyltrimethylammonium bromide (CTAB) with Sorbitol extraction buffer (Merck, Darmstadt, Germany; Štorchová et al., 2000). Sonication was used to shear genomic DNA either with Qsonica 700 (Qsonica, Newtown, Connecticut, USA), Covaris S220, or Covaris M220 (Covaris, Brighton, United Kingdom); see Appendices S1 and S2 for settings for each model. Genomic DNA was sheared to a target size of ~500 bp. DNA was already well‐fragmented for two herbarium samples, and therefore sonication was not applied. Subsequently, DNA libraries were prepared according to the manufacturer's protocol, which varied between samples (NEBNext Ultra I or Ultra II [New England BioLabs, Ipswich, Massachusetts, USA], or TruSeq [Illumina, San Diego, California, USA]). During library preparation, dual‐index primers were used for six samples (*Lactuca* L.), and for the remaining samples, single‐index primers were used. The number of PCR cycles during library preparation ranged from eight to 15 (Appendix S2). The libraries were then pooled (equimolar) in preparation for hybrid capture reactions, and the number of libraries per pool was 1, 3, 4, 18, or 24, depending on the lab. For hybrid capture reactions, the same set of probes and protocol were used for all samples (MyBaits COS Compositae/Asteraceae1kv1; Mandel et al., 2014); however, different versions of the probe kit were used (versions 1–3). Incubation temperature was always 65°C, as per the manufacturer's protocol; incubation times were 26, 27, or 36 h; and the number of cycles for amplification of the capture reactions to yield enriched libraries was either 12 or 16. Prior to sequencing, 31 of the 112 enriched libraries were spiked with unenriched library (enriched to unenriched library ratios were either 1 : 3 or 1 : 4); the remainder were not spiked. Subsequently, spiked or unspiked enriched libraries were pooled (equimolar) and the following sequencing platforms were used: HiSeq 2000 (200 cycles), HiSeq 2500 (high‐output mode; 300 cycles), HiSeq 3000 (200 cycles), NextSeq (mid‐output mode; 300 cycles) or MiSeq v. 2 (300 cycles) (Illumina).

### Data cleaning and reference‐guided assembly

Refer to Fig. 2 for the pipeline with details of data preparation and analyses. A combination of HybPhyloMaker (Fér and Schmickl, 2018) and HybPiper (Johnson et al., 2016) was used for data preparation and analyses in the following sections. The first steps in data preparation for each sample were conducted in HybPhyloMaker, a pipeline that makes use of already available software (see details below) to perform Hyb‐Seq data analyses. Specifically, HybPhyloMaker steps 1–3 were used for raw read quality filtering, mapping to targets, and contig assembly (top of Fig. 2). Within the HybPhyloMaker pipeline, adapter trimming and quality filtering steps were conducted using Trimmomatic v.0.32 (Bolger et al., 2014). Quality filtering parameters were as follows: bases at read ends with quality <Q20 were discarded, the remaining parts were trimmed if the average quality in a 5‐bp window was <Q20, and whole reads were removed if read length fell below 36 bp after trimming. FastUniq v.1.1 (Xu et al., 2012) was then used for duplicate removal, also within HybPhyloMaker. Exon matrices were built using HybPhyloMaker (step A in Fig. 2). The probes for hybrid capture of the COS loci were developed by Mandel et al. (2014) via BLAST searches of expressed sequence tags (ESTs) from three divergent Asteraceae species (*Helianthus annuus* L. [sunflower; Asteroideae], *Lactuca sativa* L. [lettuce; Cichorioideae], and *Carthamus tinctorius* L. [safflower; Carduoideae]) against single‐copy *Arabidopsis* Heynh. genes. There are two to three reference sequences from those three different species for each of the 1061 COS loci. A single reference sequence (“pseudoreference”) is necessary to perform reference‐guided assemblies in HybPhyloMaker using Burrows–Wheeler Aligner (BWA). Therefore, we used the reference EST sequences of the Asteraceae COS loci to build three genome‐specific reference sequences (sunflower, lettuce, safflower) in Geneious 6.1.5 (Biomatters Ltd., Auckland, New Zealand). Mapping was then performed three times for all samples, using the different pseudoreferences in HybPhyloMaker. HybPhyloMaker generates contigs by mapping to the reference sequence using BLAT (Kent, 2002) and calling a consensus sequence for each locus using Kindel v. 0.1.4 (Constantinides and Robertson, 2017). A 70% majority rule consensus was applied for positions with >4× coverage (Carlsen et al., 2018). The sequences for each sample after mapping to the three different references were processed to obtain the maximum numbers of loci per sample. Reference sequences used for mapping in HybPhyloMaker and scripts for building the final set of loci for each sample are available at https://github.com/tomas-fer/Asteraceae.

**Figure 2:**
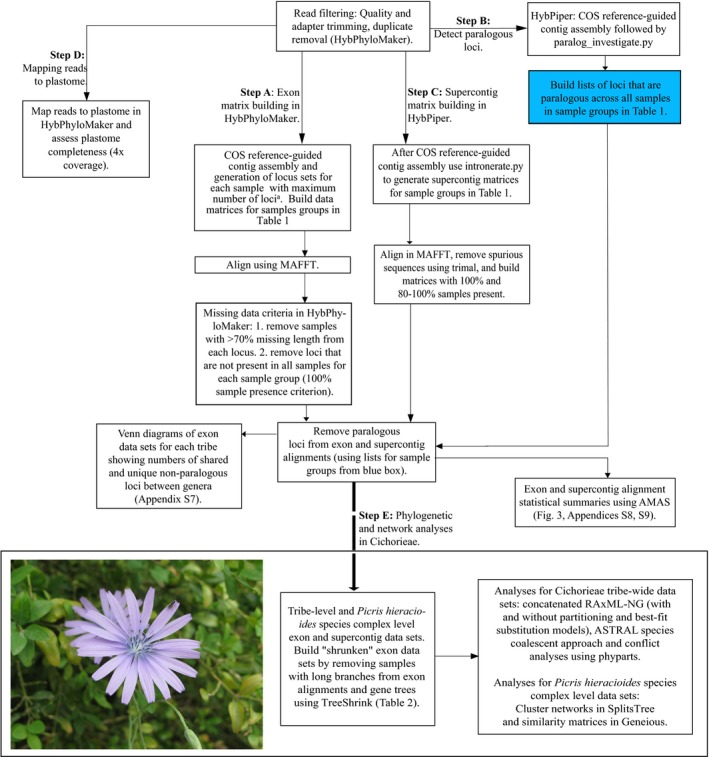
Pipeline for preparation and analyses of exon and supercontig data sets for sample groups in Table 1 in HybPhyloMaker (exon matrices, step A) and HybPiper (paralogous locus detection and supercontig matrices, steps B and C). Mapping to the plastome is described in step D and details of analyses within Cichorieae are provided in step E. ^a^See https://github.com/tomas-fer/Asteraceae for pipeline to build exon data sets per sample after contig assembly in HybPhyloMaker. Photo: *Lactuca perennis* (Cichorieae), growing below Rougon, Provence, France (photo by N. Kilian); voucher: *N. Kilian 10298* (BM).

HybPhyloMaker does not identify potentially paralogous loci; instead, the consensus calling after assembly represents the most abundant sequence, which is considered to be the ortholog. Although analyses of data sets with paralogs can be accurate under the MSC model (Du et al., 2019a), they may still cause inaccurate phylogeny estimations, especially for lineages that are rapidly evolving and show rampant WGDs (Mandel et al., 2014, 2015, 2017). Therefore, in parallel to the HybPhyloMaker pipeline, cleaned data (after adapter trimming, quality filtering, and duplicate removal in HybPhyloMaker) were also processed in HybPiper v. 1.2 in order to identify potentially paralogous loci (step B in Fig. 2). The single reference file for read mapping in HybPiper contained all reference sequences for each of the 1061 reference loci; read mapping was conducted using BWA, and contig assembly was performed using SPAdes in HybPiper (see Fér and Schmickl [2018] for further comparisons between HybPiper and HybPhyloMaker). Paralogous loci were flagged in HybPiper using the following (default) settings: multiple long‐length contigs (>85% of the reference locus) with similar coverage (within 10× of each other) that mapped to a reference locus.

### Building data matrices, alignments, and summary statistics (by sample group)

Preliminary analyses had shown that mapping to references and assembly in HybPhyloMaker led to higher numbers of target loci captured per sample compared to processing data in HybPiper (Appendix S3). Therefore, for building matrices of exon regions it was beneficial to use a combined approach with HybPhyloMaker (step A in Fig. 2; to obtain the maximum number of loci per sample) and HybPiper (step B in Fig. 2; to identify potentially paralogous loci that should be removed from the set of loci). For each sample group, exon matrices were built using the following criteria (step A in Fig. 2): First, exon alignments for each sample group were conducted using MAFFT v. 7.409 (Katoh and Standley, 2013) in HybPhyloMaker. Second, we removed samples with >70% missing data from the particular locus alignment. Next, we applied a 100% sample presence criterion, and loci that were not present in all samples were removed from each sample group (species, species complex, genus, tribe; Fig. 2, Table 1). Lists of potentially paralogous loci from HybPiper were generated for each sample group (blue box in Fig. 2). These loci were then removed from all samples in each data set following the HybPhyloMaker pipeline (Fig. 2; see Table 1 for final numbers of non‐paralogous loci per sample group). Therefore, alignments contained non‐paralogous loci only with <70% missing data and 100% of samples for each sample group (Fig. 2). AMAS v. 0.98 (Borowiec, 2016) and MstatX (Collet, 2012) were used to retrieve summary statistics for alignments of each sample group in HybPhyloMaker (Fig. 2). Loci were then concatenated for each sample using AMAS, and summary statistics were retrieved for the concatenated alignments of each sample group using the same approach as above. To investigate the proportions of group‐specific and shared non‐paralogous COS loci between sample groups within each taxonomic level (Table 1), area‐proportional Venn diagrams were produced using BioVenn (Hulsen et al., 2008).

In addition to generating sequences for the 1061 targeted coding regions, we assembled sequences of the so‐called “splash‐zone” (exons + flanking intron regions; step C in Fig. 2; Weitemier et al., 2014) using intronerate.py within HybPiper (Johnson et al., 2016). Matrices of supercontigs (exons + introns) for each sample group in Table 1 were aligned using MAFFT. Heliantheae and *Lipochaeta* sample groups were excluded from supercontig alignments due to poor capture for some samples in HybPiper (<300 genes with sequences in HybPiper; Appendix S1). The sequences recovered after running intronerate.py in HybPiper may represent introns or mis‐assembled contigs; therefore, it is recommended to remove spurious sequences from alignments (Johnson et al., 2019). A number of tools for sequence alignment trimming and masking are available, including Gblocks (Talavera and Castresana, 2007), BMGE (Criscuolo and Gribaldo, 2010), Zorro (Wu et al., 2012), and trimAl (Capella‐Gutiérrez et al., 2009). We used trimAl to remove spurious sequences using ‐resoverlap and ‐seqoverlap. Based on a preliminary assessment of two different thresholds in trimAl for two data sets (the *P. hieracioides* species complex and Cichorieae tribe‐wide data sets), the following values for minimum sequence overlap were applied to all data sets in Table 1: ‐resoverlap and ‐seqoverlap were 0.65 and 70, respectively (Appendix S4; alignments are available at https://datadryad.org/review?doi=doi:10.5061/dryad.60vb576). In addition, we applied the ‐gappyout parameter, which efficiently removes poorly aligned regions (Capella‐Gutiérrez et al., 2009). AMAS was then used to retrieve summary statistics for alignments of supercontigs. Due to the conservative trimming approach of supercontig alignments, which was necessary to remove spurious sequences, a large number of data sets had <100% samples; therefore, we summarized alignments containing both >80% and, when possible, 100% of samples.

### Analyzing different data sets of COS loci at different taxonomic depths within Cichorieae

The pipeline is presented in Fig. 2, and the data sets and analyses used are available in Table 2. We first analyzed the exon alignments of the Cichorieae tribe‐wide sample group, which consisted of *Gundelia tournefortii* L. as the outgroup taxon and ingroup species that were selected according to the composition of Clade 4 in the Cichorieae‐wide nrITS tree in Kilian et al. (2009) and Tremetsberger et al. (2012). Four of the five subtribes from Clade 4 were represented: Lactucinae (six *Lactuca* species), Crepidinae (*Taraxacum kok‐saghyz* L. E. Rodin, *Nabalus albus* (L.) Hook.), Hyoseridinae (six *Sonchus* L. species), and Hypochaeridinae (*Leontodon tingitanus* (Boiss. & Reut.) Ball and seven *Picris* L. taxa, comprising five species and three subspecies within *P. hieracioides*). *Hieracium alpinum* is subject to ongoing phylogenetic studies and is a member of a more distant clade within Cichorieae (Clade 5; Kilian et al., 2009; Tremetsberger et al., 2012); samples of this species were therefore excluded from Cichorieae‐wide phylogenetic analyses. Therefore, the Cichorieae exon alignments for phylogenetic analyses consisted of 24 samples (Table 2). We investigated the impact of different analyses (concatenated ML vs. a species coalescent approach using ASTRAL) of the tribe‐exon‐complete data set (218 loci; 100% samples in all alignments) on phylogenetic resolution and topological estimation (Table 2). Concatenated non‐partitioned data sets (using the model GTR+G) and partitioned data sets were analyzed using ML in RAxML‐NG v. 0.8.1 (Kozlov et al., 2019). For the partitioned data set, we used PartitionFinder v. 2 (Lanfear et al., 2016), with user‐defined data blocks according to gene partitions and codon positions to estimate optimal partitioning schemes and substitution models. We used a relaxed hierarchical clustering algorithm, fixing the proportion of analyzed partitioning schemes to 10, as recommended for large phylogenomic data sets (>100 loci; Lanfear et al., 2014; settings: –search rcluster and –rcluster‐percent 10). This approach tests three substitution models (GTR, GTR+G, and GTR+I+G) and enables a good balance between computational efficiency and performance for large data sets in PartitionFinder (Lanfear et al., 2014). To estimate branch support, we performed 200–450 bootstrap (BS) replicates, with the number of replicates varying depending on when bootstrapping converged; we checked for convergence using –bsconverge in RAxML‐NG. Tree likelihood for analyses with and without partitioning was estimated and compared according to log likelihood and corrected Akaike information criterion (AICc) values. The optimal branch linkage model for the partitioned data sets (brlen; linked, scaled, and unlinked) was tested according to log likelihood, AICc, and Bayesian information criterion (BIC) values of the trees using –evaluate and –brlen in RAxML‐NG.

**Table 2:** Cichorieae exon and supercontig data sets and analyses.

Taxonomic level (outgroup)	No. of samples	No. of non‐paralogous loci (No. of gene trees)	Data set name	% samples per alignment (No. of samples)	TreeShrink analyses^b^	% loci with parsimony informative characters	No. of base pairs^c^	Analyses^d^
Tribe level: Cichorieae‐wide (*Gundelia tournefortii*)	24^a^	218	Tribe‐exon‐complete	100	NA	100	59,722	ML concatenated data sets non‐partitioned and partitioned and ASTRAL
			Tribe‐exon‐shrunken	79–100 (19–24)	72	100	59,722	
		201	Tribe‐supercontig	75–100 (18–24)	NA	100	177,456	
Species complex: *Picris hieracioides* (*P. amalecitana*)	9	610	Picris‐610exon‐complete	100	NA	65.5	156,731	Network in SplitsTree and similarity matrix in Geneious
			Picris‐610exon‐shrunken	88–100 (8–9)	38	64.4	156,638	
		576	Picris‐supercontig	75–100 (7–9)	NA	99.6	596,166	
		218	Picris‐218 exon‐complete	100 (9)	NA	67.4	59,290	
			Picris‐218 exon‐shrunken	88–100 (8–9)	34.8	67	58,730	

a The number of samples in tribe‐wide tree analyses is 24 due to the exclusion of six *Hieracium alpinum* samples that were included in the non‐paralogy and alignment summary statistics in Tables 1 and 2.

b Percentage of all alignments/gene trees that were shrunk, i.e., percentage of alignments containing samples with long branches that had been removed from the corresponding ‐complete data set using TreeShrink.

c Number of base pairs in concatenated alignments.

d ML = RAxML‐ng; ASTRAL = ASTRAL III coalescent species tree.

For the tribe‐exon‐complete data set, we also used ASTRAL III, a method that is consistent under a coalescent process (Zhang et al., 2018). ASTRAL has been shown to account for incomplete lineage sorting, it uses maximum quartet support for species tree estimation, and it calculates the local posterior probabilities on nodes using gene trees (Mirarab et al., 2014). Gene trees for each locus were first estimated using RAxML with the GTR+GAMMA model and 100 rapid BS replicates (Stamatakis, 2014). Species trees were then obtained in ASTRAL by calculating quartet scores on each node, local posterior probabilities, and number of quartet trees among the gene trees. In species tree approaches, samples are typically assigned to taxa, but within the *P. hieracioides* species complex, taxon boundaries are unclear and *P. hieracioides* s.s. is non‐monophyletic (Slovák et al., 2014). However, the three *P. hieracioides* subspecies were each shown to be monophyletic based on AFLP data (Slovák et al., 2012) and according to plastid and nrITS data in Slovák et al. (2018), although sampling differed between the studies. In the present study, two individuals of *P. hieracioides* subsp. *hieracioides* and of *P. hieracioides* subsp. *hispidissima* were included, we therefore conducted a first analysis in ASTRAL with these samples unassigned (“blind” approach; see Villaverde et al., 2018) and another where they were assigned to their respective subspecies as revealed by the “blind” approach. Only one accession of *P. hieracioides* subsp. *umbellata* was included.

Topological inferences were inconsistent between the initial ML and ASTRAL analyses of the tribe‐exon‐complete data set described above, and we aimed to investigate the causes of this in the next steps. Specifically, *P. amalecitana* was resolved within the *P. hieracioides* species complex in the ML tree and outside of it in the ASTRAL tree; the latter was in accordance with previous studies on *Picris* (Appendix S5; Slovák et al., 2018). Discordance may be caused by biological processes such as incomplete lineage sorting or hybridization; however, this can also be caused by erroneous gene tree estimation, which can lead to misleading species tree reconstructions (Mai and Mirarab, 2018). Furthermore, the presence of problematic sequences in alignments may be detrimental for concatenation approaches such as ML tree reconstruction and cluster network analyses. We therefore explored the possible causes of incongruence between the ML and ASTRAL trees based on the exon‐complete data set by testing (1) whether topological inference in ML analyses is influenced by long branches and therefore the incongruence observed was due to a methodological artifact (long‐branch attraction), and (2) whether analyzing regions with more PI characters than exon‐only alignments influences topological inference in the different analyses (supercontigs; 201 alignments of exon + intron regions containing >70% [17–24] samples; Table 2). We allowed <30% samples missing per supercontig alignment for Cichorieae analyses in order to maximize numbers of loci for analyses. Lastly, (3) we also assessed gene tree conflict for all tribe‐level data sets in Table 2 using the software phyparts to test levels of support for all species trees (Stephens et al., 2015; see “Conflict analyses” below for details about phyparts; Table 2; step E in Fig. 2). Furthermore, we tested whether subsampling the locus data set at shallower taxonomic depths, in this case at the *P. hieracioides* species complex–level (with *P. amalecitana*), was more informative for inferring relationships within the species complex, compared to broad taxonomic sampling across the entire tribe (Cichorieae‐wide; Table 2). We used TreeShrink v. 1.3.1 to detect samples that had unexpectedly long branches in the ML gene trees based on the tribe‐complete‐exon data set (false‐positive tolerance level 0.10; Table 2; Mai and Mirarab, 2018) and removed those samples from gene trees and alignments generating the so called “tribe‐exon‐shrunken” data set (Mai and Mirarab, 2018; Table 2). Species complex–level data sets were concatenated and cluster network analyses were conducted in SplitsTree v. 4 (Huson and Bryant, 2006), and cluster support was assessed following 1000 BS replicates. Similarity matrices for all species complex–level data sets were estimated in Geneious. Levels of resolution and topological inferences within *P. hieracioides* were then compared between all analyses in Table 2.

In summary, the following data sets were analyzed (Table 2; step E in Fig. 2): three at the tribe‐level containing 24 samples with 218 exons (complete and shrunken) and with 201 supercontigs (complete). For the *P. hieracioides* species complex–level analysis (nine samples; sensu Slovák et al., 2018), two of the data sets contained 610 exons (‐complete and ‐shrunken), the third data set contained 576 supercontigs, and the fourth and fifth data sets contained the 218 exons from the tribe‐wide data sets, but only consisting of the *P. hieracioides* species complex–level samples (‐complete and ‐shrunken; Table 2).

### Conflict analyses

For all tribe‐wide data sets in Table 2 (tribe‐exon‐complete, ‐shrunken, and ‐supercontig), we used a bipartition‐based approach in phyparts (Smith et al., 2015) to test for conflict between gene trees and support for the species trees generated using ASTRAL and partitioned RAxML‐NG analyses; we applied a minimum 80% BS threshold. The gene and species trees were rooted using R, package ape (Paradis et al., 2004; Paradis and Schliep, 2019). Resulting pie charts were mapped onto a tree using phypartspiecharts.py (available at https://github.com/mossmatters/MJPythonNotebooks). Phyparts requires the same outgroup in all gene trees and the species tree. Therefore, for the tribe‐exon‐shrunken and ‐supercontig data sets, the number of gene trees was reduced to 201 and 139, respectively, because the outgroup taxon (*Gundelia tournefortii*) was missing in 17 and 62 alignments, respectively.

### Off‐target loci: Plastome

We measured the number of reads mapped to the off‐target plastome and the proportion of plastome recovered across all samples. To assess what proportion of the plastome was recovered per sample, cleaned reads for each sample were mapped to the sunflower (*H. annuus*) plastome (KU315426) in HybPhyloMaker (step D in Fig. 2). If the coverage was <4×, then N was called in the consensus. The percentage of the plastome recovered was calculated as the proportion of non‐N characters in the consensus.

### Variables influencing numbers of reads mapped to targets and off‐target plastomes

Here we explored the impact of wet‐lab steps on number of reads mapped to targets in HybPiper and to the off‐target plastome in HybPhyloMaker. In HybPhyloMaker, reads were mapped to the three genome‐specific reference sequences (pseudoreferences) separately; each locus was then selected from the specific pseudoreference for which that locus had the least missing data (step A in Fig. 2 and https://github.com/tomas-fer/Asteraceae). Numbers of reads mapped to each separate pseudoreference genome can be summarized (Appendix S3). However, the number of reads mapped to all loci using all three pseudoreferences in HybPhyloMaker (when selecting the “best” reference for each exon separately) could not be estimated in this study. Instead, we worked with the number of reads mapped to targets according to HybPiper (Appendix S1; reads had been cleaned using HybPhyloMaker prior to mapping in HybPiper; Fig. 2). First, we tested for correlations between total number of reads sequenced per sample and the following variables: number of reads mapping to targets, number of target genes mapped, number of targets with >25, >50, and >75% of the reference length (all according to HybPiper), reads mapping to the off‐target plastome, and percentage of plastome recovered (>4× coverage; according to HybPhyloMaker), using Pearson's correlation tests in R v. 3.5.3 (R Core Team, 2014) with the function cor.test; all *P* values were corrected using the function “p.adjust” in R (Appendix S6). Subsequently, we explored the impact of different combinations of wet‐lab steps on number of reads mapping to targets and off‐target plastome. Because this study incorporated samples from different labs, we organized samples into nine wet‐lab groups that were processed according to different combinations of the following steps: probe kit version, sequencing platform, library preparation kit, number of amplification cycles during hybrid capture, incubation time, and number of samples in the hybrid capture pool (Table 3). It was important to separate the groups according to the myBaits probe kit, sequencing platforms, and library preparation kits because preliminary ANOVA conducted in R revealed that they significantly influenced number of reads mapped to targets. Earlier myBaits probe kit versions recovered fewer COS loci but more of the plastome (data not shown). Although a number of steps overlap between groups (i.e., number of PCR cycles; Table 3), this approach was informative for summarizing and exploring read mapping according to different lab processes with the data available. Box‐and‐whisker plots were generated to show numbers of reads mapped for the different wet‐lab groups in R (Table 3). In addition to the lab groupings in Table 3, other variables likely influence number of reads mapping to targets and off‐targets, including leaf material type (fresh, silica‐dried, or herbarium used here), library spike (when an enriched library was spiked with unenriched library prior to sequencing; 31 of our samples were spiked), and genome size (*C* values). Estimations for genome size were already available for 34 species; for the remaining species, average genome size values for the respective genus or tribe were used (see references for genome sizes in Appendix S1). We tested for correlations between genome size and numbers of reads mapped (as above; both scaled; Appendix S6).

**Table 3:** Grouping of samples according to combinations of wet‐lab steps.^a^

Group	Probe kit version	Sequencing platform	Library preparation kit	Incubation time^b^	No. of amplification cycles^c^	No. of samples in hybrid capture pool
1	1	HiSeq 2000	TruSeq	36	16	1
2	2	HiSeq 2500	NEB Next Ultra II	36	16	4
3	3	HiSeq 3000	NEB Next Ultra II	36	16	4
4	3	NextSeq	NEB Next Ultra II	27	16	3
5	2	MiSeq	NEB Next Ultra I	36	16	1
6	2	MiSeq	NEB Next Ultra II	36	16	1 or 4
7	2	MiSeq	NEB Next Ultra I	26	12	24
8	3	MiSeq	NEB Next Ultra II	36	16	1, 3, or 4
9	3	MiSeq	NEB Next Ultra I	26	12	18 or 24

a Refer to Appendix S1 for details of each sample and to Fig. 7 and Appendix S16 for analyses conducted on this data set.

b Incubation time (in hours) during hybrid capture at 65°C.

c Number of PCR cycles during hybridization capture.

We conducted Bayesian regression multilevel model fitting using package Bayesian regression analyses (brms) using Stan in R (Bürkner, 2017) to test the impact of leaf material type, library spike, and genome size on number of reads mapped to targets and the off‐target plastome, while taking into account the variation among groups in Table 3 in the response variables. Brms allows the influence of variables that may vary within the response variable to be “accounted for.” This package uses the programming language Stan within R to set up single or multilevel regression models that are potentially non‐linear, unlike other regression methods that rely on linear models for distribution. The number of reads mapped to targets and the off‐target plastome are referred to here as response variables, and variables that may influence those factors (leaf material type, sample spike, and genome size) are the predictor variables. The following settings were used in brms: Adapt delta was set to 0.999 (the tuning parameter in the NUTS sampler for Hamiltonian Monte Carlo), chains = 4, iter = 3000, warmup = 600, seed = 10. We checked that chains converged (indicated when “Rhat”, the potential scale reduction factor, was equal to 1). To interpret the effect of the predictor variables on the response variables, we used the estimate values (means) and the marginal effects (function “marginal_effects” within package brms). Refer to https://github.com/katy-e-jones/Asteraceae/blob/master/lab_modelling for the script used in R to set up the brms regression model.

## RESULTS

### Hybrid capture sequencing of the COS loci

Hyb‐Seq data were generated for 112 samples across Asteraceae (Appendix 1). The average number of reads per sample was 5,044,708, ranging from 70,008 in *Lipochaeta subcordata* A. Gray to ~30.9 million for *Chresta harleyi* H. Rob. (Appendix S1). On average, 1,031,853 (39.2%) of total cleaned reads were mapped to the target COS loci, an average of 1025 of the 1061 targets were mapped, 954 targets had >30% the reference length after mapping in HybPhyloMaker, and 564 targets were >75% of the reference length, according to mapping in HybPiper (Appendix S1).

### Exon alignments

Data for each sample were arranged into sample groups (Table 1) and cleaned according to the criteria listed in step A in Fig. 2. Amounts of loci removed at each stage of sample group data trimming are given in Appendix S7 for exon alignments built in HybPhyloMaker (loci removed due to >70% data missing, not being present in other samples in the sample group [100% sample presence criterion], or potentially paralogous according to HybPiper). For sample group alignments (Table 1), an average of 76 loci were not captured per sample, on average 17 loci had >70% missing data per sample, and 248 loci were removed per sample because they were missing in other samples in the respective sample group (100% sample presence criterion; Appendix S7). An average of 434 loci per sample group were flagged as paralogous and removed (Table 1). After data trimming, the final species‐level alignments contained 647, 664, and 658 non‐paralogous COS loci for *Hieracium alpinum*,* Picris hieracioides*, and *Carlina vulgaris*, respectively. At the genus level, that value ranged from 306 for *Senecio* to 702 for both *Cousinia* and *Helianthus* (the genus‐level average was 510 loci). At the tribe level, the number of non‐paralogous COS loci after data cleaning ranged from 213 in Cichorieae (30‐sample data set) to 465 in Cardueae (the tribe‐level average number of non‐paralogous loci was 325 loci).

### Non‐paralogous loci specific to sample groups

Ten genera and one species complex were sampled with more than four species each from five different tribes (Heliantheae, Vernonieae, Senecioneae, Cardueae, and Cichorieae). Venn diagrams in Appendix S8 show that there were non‐paralogous loci unique to each genus within their respective tribes (genus‐specific non‐paralogous loci); the proportions of non‐paralogous loci that were genus specific ranged from ~5% for *Lactuca* and *Picris* in Cichorieae to 38.7% for *Lipochaeta* in Heliantheae.

### Alignments of the off‐target splash‐zone (supercontigs)

After mapping and assembly in HybPiper, supercontig sequences (exon + flanking introns) were generated for samples in each sample group (Fig. 2). The number of supercontig alignments containing 100% and 80% (or >75%, see Appendix S9) of samples showed significant variation between sample groups after potentially paralogous loci and spurious sequences had been removed (Appendix S9). Numbers of supercontig alignments across all sample groups that contained >80% samples ranged from 160 for *Senecio* to 650 for *Sonchus*; this reduced to 0 and 565 alignments with 100% samples for *Senecio* and *Sonchus*, respectively. Very few supercontig alignments remained for Heliantheae, *Lipochaeta*, and *Antennaria* after trimming; furthermore, these alignments were non‐informative, and they are therefore excluded from the results described below. Samples with the highest numbers of sequenced reads and numbers of reads mapped to targets were also members of groups with the highest numbers of supercontig alignments that remained after trimming compared to all other sample groups (Appendices S1, S9).

### Exon and supercontig alignment lengths and parsimony informative characters

**Figure 3:**
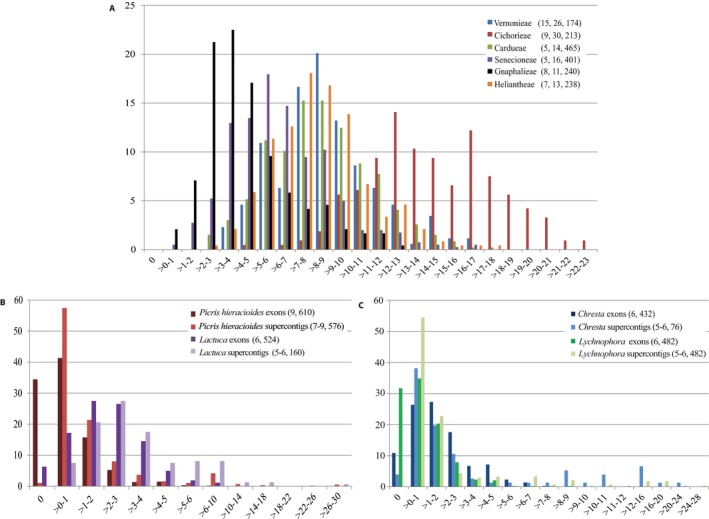
Percentages of parsimony informative (PI) sites (*x*‐axis) and conserved orthologous set loci (*y*‐axis) in alignments of non‐paralogous loci at multiple taxonomic levels across Asteraceae. (A) PI percentages for the tribe‐level alignments of target exon sequences generated using HybPhyloMaker; color coding for tribes is described in the legend, with numbers of genera, species, and loci included in the analyses given in parentheses. (B–C) PI percentages for alignments of the target exon sequences and of the exon sequences with flanking intron regions (supercontigs) generated using HybPiper (using intronerate.py), in (B) the Cichorieae at species complex level (*Picris hieracioides* complex) and genus level (*Lactuca*) and (C) the Vernonieae at genus level (*Chresta* and *Lychnophora*); color coding for taxon names is described in the legend, with numbers of samples and loci included in the alignments given in parentheses.

For all tribes, every exon and supercontig alignment had PI sites (Fig. 3A; Appendices S10, S11); the same was observed for supercontig tribe‐level alignments, but with even higher percentages of PI sites (with the exception of tribe Heliantheae for which supercontig alignments were not generated; see Appendix S9). The percentages of PI sites for exon alignments ranged from ~0.5–13%, ~0.5–16.5%, and ~0.5–17% in Gnaphalieae, Senecioneae, and Vernonieae, respectively, ~2.5–17.5% in Cardueae, ~2.5–18% in Heliantheae, and ~4.5–22.5% in Cichorieae (Fig. 3A). Higher percentages of PI sites were observed in Cichorieae exon alignments than for all other tribes; 90.6% of Cichorieae alignments contained >10% PI sites (maximum 22.5%), whereas 25.9%, 26%, 7.7%, 3.7%, and 19.9% of alignments in Vernonieae, Cardueae, Senecioneae, Gnaphalieae, and Heliantheae, respectively, had >10% PI sites (Fig. 3A).

At the genus level and below (i.e., species complex and species levels), there were alignments without PI sites and the proportion of alignments with zero PI sites was markedly lower for supercontig alignments (exons + flanking intron regions) compared to those of exons only. See Fig. 3B, C for species complex–level *Picris hieracioides* and genus‐level *Lactuca* (Cichorieae), *Chresta*, and *Lychnophora* (Vernonieae). For the remaining sample groups, see Appendices S9 and S10 for supercontig and exon alignment summary statistics, respectively. (We did not summarize supercontig alignments of *Antennaria* or *Lipochaeta* due to poor target capture in HybPiper and loss of samples during alignment trimming; data not shown.) Most exon alignments had between ~0.5–4% PI sites, and a small number of them reached PI values >4% (maximum percentage of PI sites was 10 for alignments of the *P. hieracioides* species complex; Fig. 3B, C). Supercontig alignments at the genus level and below reached >10% PI, whereas no exon alignment at that taxonomic level contained >10% PI sites. For the concatenated exon alignments, the proportion of PI sites ranged from 0.4–0.6% at the species level and from 0.2–2.2% at the genus level for *Sonchus* and *Lactuca*, respectively. (*Lipochaeta* with three samples had zero PI sites, but the proportion of variable sites for that sample group was 2.5.) At the tribal level, PI sites of concatenated exon alignments ranged from 4.6% for Gnaphalieae to 15% for Cichorieae (Appendix S11).

Across non‐paralogous locus data sets for sample sets in Table 1, mean exon alignment length was 256 bp; lower, middle, and upper quartiles were 149, 237, and 335 bp, respectively. The longest exon alignment was 735 bp (Appendix S10). The mean supercontig alignment length was 1015 bp; lower, median, and upper quartiles were 733, 880, and 1175 bp, respectively (see Appendix S9 for supercontig alignment summary statistics). A small percentage (~1%) of supercontig alignments across all sample groups reached >2500 bp.

### Removal of long branches from the tribe‐exon‐complete data set using TreeShrink

In the Cichorieae tribe‐exon‐shrunken data set, 72% of alignments and gene trees were “shrunken,” meaning that samples with long branches had been removed from 72% of the tribe‐exon‐complete alignments. A maximum of five samples were removed from an alignment; therefore, the tribe‐exon‐shrunken data set contained 19–24 samples (79–100%; Table 2). In the Picris‐610exon‐shrunken and Picris‐218exon‐shrunken data sets, 38% and 38.4% of alignments from the Picris‐610exon‐complete and Picris‐218exon‐complete data sets were shrunken, respectively, and a maximum of one sample was removed from an alignment (88–100%; Table 2).

### Phylogenetic analyses of different data sets of the COS loci: Tribe Cichorieae as a model

**Figure 4:**
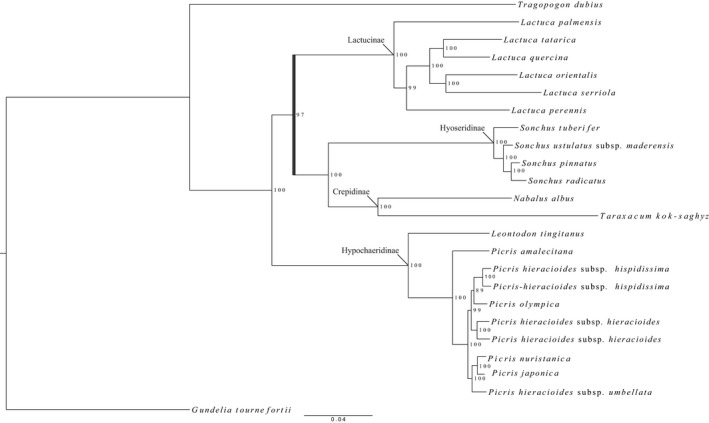
RAxML‐NG maximum likelihood tree (with partitioning applying the scaled branch linkage model) of the Cichorieae‐wide supercontig concatenated data set (Table 2; 201 loci). Subtribe names are indicated next to their corresponding nodes. The scale bar (bottom) corresponds to the expected mean number of nucleotide substitutions per site. The dark blue bar corresponds to the subtribal‐backbone node that is well resolved in this tree but unresolved in other analyses (Appendices S5, S13).

For ML analyses in this study, we consider BS values of >95% as well‐supported. Log likelihood and AICc scores were higher for ML trees with partitioning and substitution models, compared to those without partitioning (Appendix S12). To assess the optimal brlen model, we used AICc and BIC values, because log likelihood was not always in agreement with AICc and BIC; scaled brlen was the optimal model for all tribe‐wide data sets in Table 2 (Appendix S12). The tribe‐supercontig ML tree is presented in Fig. 4, the tribe‐supercontig ASTRAL tree and tribe‐exon‐shrunken ML (with partitioning and substitution models) and ASTRAL trees are available in Appendix S13, and the tribe‐exon‐complete ML and ASTRAL trees are available in Appendix S5. Every subtribe sampled from Clade 4 in Kilian et al. (2009) received full statistical support in all ML and ASTRAL analyses (100% BS and 1 posterior probability [PP]) of the tribe‐wide data sets in Table 2 (i.e., Lactucinae, Crepidinae, Hyoseridinae, and Hypochaeridinae). A sister relationship was observed between subtribes Crepidinae and Hyoseridinae with full statistical support in all ML and ASTRAL trees. At shallower taxonomic levels (intergeneric), all three genera with multiple taxa sampled received full statistical support in all analyses (*Lactuca*, *Picris*, and *Sonchus*; Fig. 4; Appendices S5, S13). At the shallowest nodes (intrageneric), resolution within *Sonchus* varied depending on the analysis. All nodes received >95% BS in all ML analyses, whereas ASTRAL analyses showed low resolution at the shallower nodes; only two of the nodes within *Sonchus* were well‐supported. In ML analyses of the tribe‐supercontig data set, the subtribal backbone was fully resolved, whereas this was unresolved in other trees (Fig. 4 vs. Appendices S5, S13). Thus, there was a sister relationship between Hypochaeridinae and a clade (100% BS) containing Lactucinae as sister to the clade with Hyoseridinae and Crepidinae (97% BS; Fig. 4). Node support in the RAxML‐NG tribe‐supercontig tree was markedly higher compared to the ASTRAL tree for the same data set (Fig. 4 vs. Appendix S13), and compared to the ML and ASTRAL trees based on the tribe‐exon‐complete and ‐shrunken data sets (Appendices S5, S13). All nodes with the exception of one received >96% BS in the tribe‐supercontig ML tree (Fig. 4). Resolution, discordances in topological inferences, and clustering within the *Picris* clade among all data sets and analyses are described below.

### *Picris hieracioides* species complex resolution and conflict analyses

**Figure 5:**
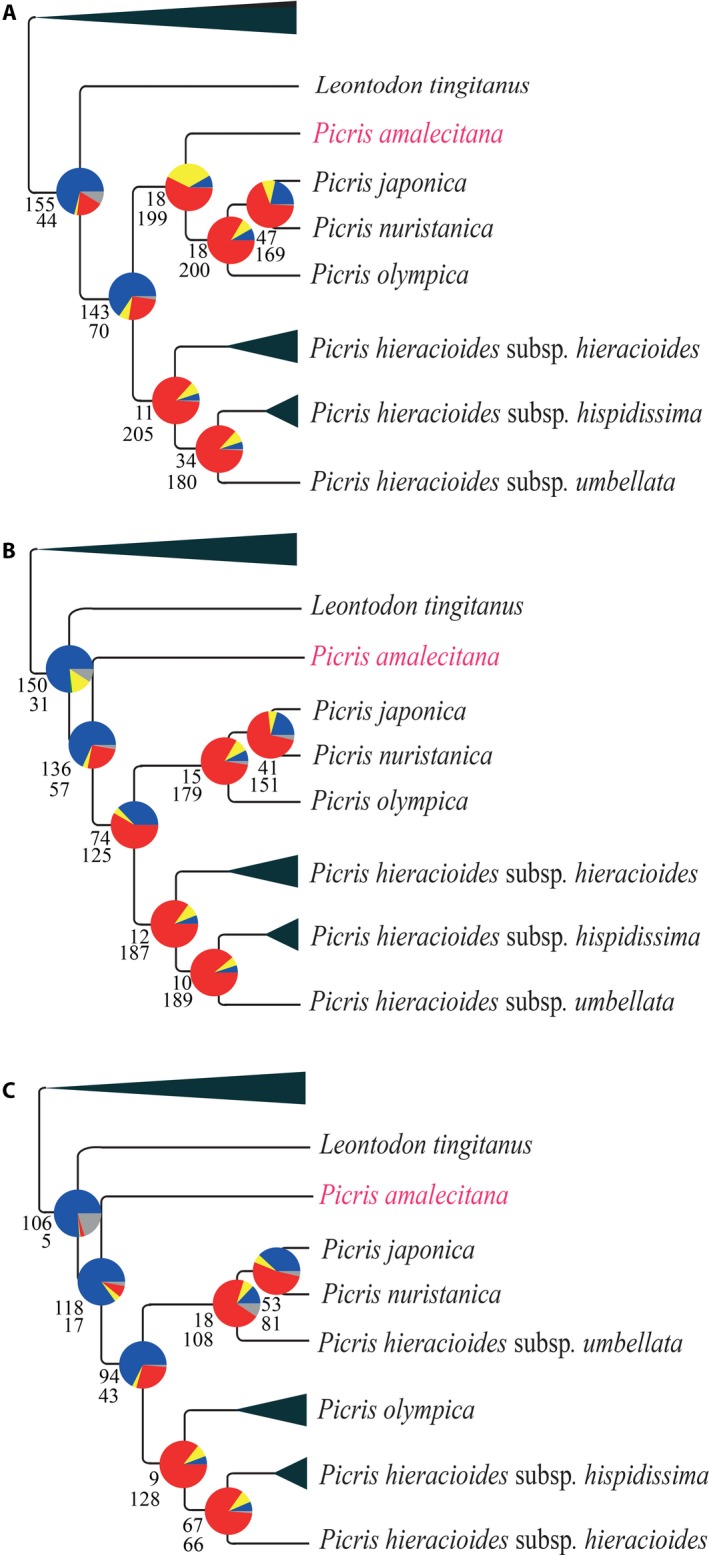
Comparisons of resolution and topological inferences within the *Picris hieracioides* species complex based on RAxML‐NG analyses of the Cichorieae tribe‐wide concatenated and partitioned data sets in Table 2, including summaries of conflicting and concordant gene trees. (A) tribe‐exon‐complete, (B) tribe‐exon‐shrunken, and (C) tribe‐supercontig data sets. For each branch, the top number indicates the number of gene trees concordant with the tree at that node and the bottom number indicates the number of gene trees in conflict with that node. The pie charts present the proportion of gene trees that support that clade (blue), the proportion that support the main alternative topology for that clade (yellow), the proportion that support the remaining alternative topologies (red), and the proportion that inform (conflict or support) that clade that have <50% bootstrap support (gray). For summaries of conflicting and concordant gene trees with the ASTRAL and all conflict across all nodes of the Cichorieae trees for the above data sets, see Appendix S15. *Picris amalecitana* is highlighted in pink to show its position and for comparison with Fig. 6.

In the ML analysis of the tribe‐exon‐complete data set (before removing long branches), *Picris amalecitana* was resolved within the *P. hieracioides* species complex and received 100% BS as sister to the clade containing *P. japonica*,* P. nuristanica*, and *P. olympica* (Appendix S5, Fig. 5A). In contrast, *P. amalecitana* was outside of and sister to the entire *P. hieracioides* species complex in the ML analyses of the exon‐shrunken data set (after long branches were removed) and of the supercontig‐complete data set (Fig. 5B and C, respectively), which is consistent with ASTRAL trees of all tribe‐wide data sets in Table 2 (Appendices S5, S13).

**Figure 6:**
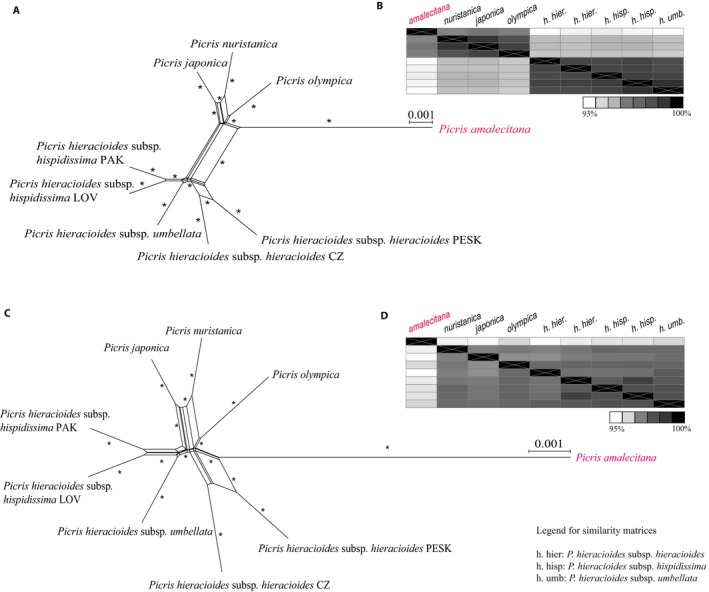
Cluster networks (A and C; 1000 bootstraps) and similarity matrices (B and D) of the *Picris hieracioides* species complex–level sample group based on alignments of different data sets. (A and B) Picris‐218exon‐complete (before removing long branches) vs. (C and D) Picris‐610‐exon‐shrunken (after removing long branches) data set. The separation of *P. amalecitana* from the *P. hieracioides* species complex is clearer as shown in C and D compared to A and B. *Indicates >90% bootstrap support. Scale bars correspond to the number of nucleotide substitutions per site. A legend for the names of samples within *P. hieracioides* used in the similarity matrices is provided at the bottom right of the figure. *Picris amalecitana* is highlighted in pink to show its position in A–D and for comparison with Fig. 5. PAK, PESK, LOV, and CZ correspond to sample codes; refer to Appendix 1 for voucher information.

Network analyses in SplitsTree were conducted for the *Picris hieracioides* species complex–level data set to investigate the impact on resolution within the complex compared to analyses based on broad taxonomic sampling (tribe‐wide data set; Table 2). Network analyses of the Picris‐exon218‐complete data set (containing loci that are non‐paralogous across the entire tribe) revealed a closer relationship between *P. amalecitana* (expected outgroup taxon) and all other taxa compared to analyses of the data set after removal of long branches (Picris‐exon218‐shrunken data set; Fig. 6A vs. C; Table 3). These relationships were also revealed by the similarity matrices (‐complete vs. ‐shrunken; Fig. 6B vs. D, respectively). Network and similarity matrices of the Picris218‐exon‐shrunken data set (Fig. 6C) were consistent with the topology in the *Picris* clade in all ASTRAL trees and the ML analysis of the tribe‐exon‐shrunken and tribe‐supercontig‐complete data sets (Appendix S13; Fig. 5B, C), but contrasted with the ML analysis of the tribe‐exon‐complete data set (Fig. 5A). Network analyses of the Picris‐610exon‐complete and Picris‐610exon‐shrunken data sets, and of the Picris‐supercontig data set are consistent with the Picris218‐exon‐shrunken data set, supporting a distant relationship of *P. amalecitana* from all other *Picris* samples (Appendix S14). Distances between samples were greater in the Picris‐supercontig‐complete data set compared to the exon‐only data sets (Appendix S14).

Conflict analyses were conducted for the entire Cichorieae data sets to investigate support within the *Picris* clade. Discussion of conflict for other nodes in the Cichorieae trees is beyond the scope of this study, but the full trees with results of phyparts are provided in Appendix S15. According to conflict analyses using the software phyparts, 18 (~8%) of all gene trees supported the clade in the tribe‐exon‐complete ML tree that resolved *Picris amalecitana* within the *P. hieracioides* species complex and 199 (91%) gene trees supported alternative topologies (Fig. 5A). In the tribe‐exon‐complete ASTRAL analysis, the clade containing *P. amalecitana* resolved outside of the species complex was supported by 143 (~65%) of all gene trees, and 70 (~32%) supported alternative topologies (five gene trees informed that clade but had <50% BS support; Appendix S15). In the tribe‐exon‐shrunken ML analysis, 136 (~67%) gene trees supported the clade containing *P. amalecitana* as sister to the *P. hieracioides* species complex and 57 (~28%) gene trees supported alternative topologies (25 [~12%] gene trees informed that clade but with <50% BS support; Fig. 5B). In the tribe‐supercontig ML tree (Fig. 5C), the clade containing *P. amalecitana* was supported by 118 (84%) of all gene trees, and alternative topologies were supported by 17 gene trees (12%; four [~2%] gene trees informed this clade but with <80% BS support).

### Variables influencing numbers of reads mapped to targets and the off‐target plastome

Correlations between total number of sequenced reads and reads mapping to targets and off‐targets, and variables associated with target capture success (numbers of targets mapped, targets with sequences, and targets with genes of different lengths after processing in HybPiper), are provided in Appendix S6, along with the percentage of the plastome recovered. The number of sequenced reads showed a positive correlation with the number and percentage of reads mapped to targets, the number of targets mapped (*P* value < 0.05), and the number of target genes reaching >50% and >75% reference sequence length (*P* value 0.03 and 0.006, respectively). No correlation was observed between number of sequenced reads and number of target genes with sequences or with target genes reaching >25% of the reference length according to HybPiper (Appendix S6). The total number of reads was positively correlated with the number of reads mapped to the off‐target plastome and percentage of the plastome recovered with >4× coverage (*P* value < 0.05), but showed no correlation with percentage of reads to plastome (Appendix S6).

**Figure 7:**
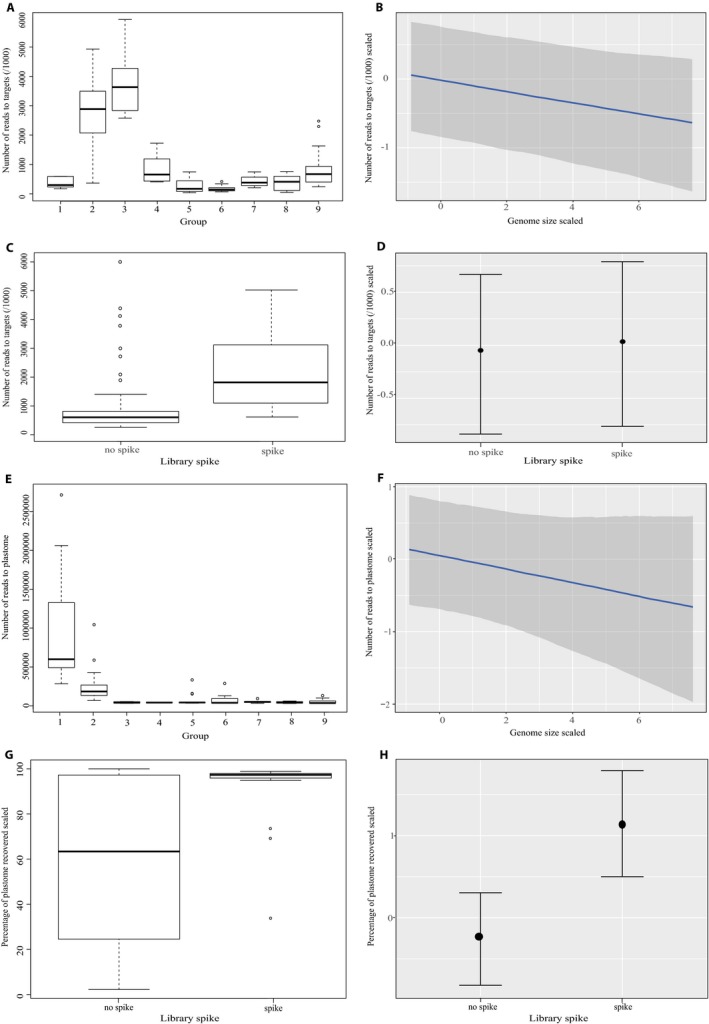
Boxplots and marginal effects graphs from Bayesian regression models using Stan in R. (A) Boxplot summarizing the variation in number of reads mapped to targets/1000 among groups 1–9. (B) Marginal effects graph showing the estimated impact of genome size (scaled) on number of reads mapped to targets/1000 (scaled) when group membership is accounted for. (C) Boxplot summarizing the number of reads mapped to targets/1000 when an enriched library is spiked with unenriched library (library spiking) or not. (D) Marginal effects graph showing the estimated impact of library spiking on number of reads mapped to targets, when group membership is accounted for (Table 3). (E) Boxplot summarizing the variation in number of reads mapped to the off‐target plastome among groups 1–9. (F) Marginal effects graph showing the estimated impact of genome size (scaled) on number of reads mapped to the off‐target plastome when group membership is accounted for. (G) Boxplot summarizing the percentage of reads mapped to the off‐target plastome with and without library spiking. (H) Marginal effects graph showing the estimated impact of library spiking on number of reads mapped to the off‐target plastome, when the group membership is accounted for (Table 3) in brms. See Table 3 for wet‐lab treatment groups 1–9. In the boxplots (A, C, E, G), thick dark lines indicate the median, boxes correspond to the third (upper edge) and first (lower edge) quartile, the dotted lines lead to the minimum and maximum values, and the circles correspond to outliers. In B and D, the blue line corresponds to the correlation coefficient and dark gray shading is the estimated error. In D and H, the circles indicate the estimated means and the vertical lines are error bars. Script used in R for brms regression models can be found here: https://github.com/katy-e-jones/Asteraceae/blob/master/lab_modelling.

Boxplots showing the variation in numbers and percentages of reads mapped to targets and the plastome among groups in Table 3, and marginal effect graphs from regression models in brms are provided in Fig. 7A–E. Samples in groups 2 and 3 had more reads mapping to targets compared to all other groups (Table 3, Fig. 7A). The average number of reads mapped to targets for samples in groups 2 and 3 were 2,769,091 and 3,852,239, respectively, and the average for all other groups combined was 680,181 reads (groups 1 and 4–9; Fig. 7A, Appendix S1). A Pearson's correlation test suggested no significant correlation between genome size and numbers of reads mapping to targets (Appendix S6). However, when the effect of group membership (Table 3) was accounted for in the brms regression model, a larger genome had a negative effect on number of reads mapped to targets (brms estimate value: −0.08; see marginal effects graph in Fig. 7B). When the sample group's membership (Table 3) was taken into account in the regression model using brms, there was no clear impact of sample spike on number of reads mapping to targets (Table 3, Appendix S1, see Fig. 7C and D). According to the brms regression model, silica‐dried samples had a slight positive effect on the number of reads mapped to targets compared to herbarium material; however, the effect was not significant (Appendix S16; fresh material samples had fewer reads mapped to targets compared to herbarium and silica‐dried samples, but were all processed using the oldest [less efficient] version of the probe kit).

Samples in group 1 had the highest number of reads mapping to the off‐target plastome compared to other groups (HiSeq 2000 and probe kit version 1; Table 3, Fig. 7E). The average number of reads mapping to the plastome in group 1 was 1,048,243, whereas the average across all other groups was 63,727 reads. A negative correlation was observed between genome size and number of reads mapping to plastome, but this was not significant according to Pearson's correlation coefficient. However, when group was taken into account in the brms regression model, large genomes had a negative effect on number of reads mapping to the off‐target plastome (Table 3, Fig. 7F). The average number of reads mapped to the plastome when a sample was spiked and not spiked was 2,044,474 and 645,690, respectively. Brms regression models showed that sample spike had a minimal effect on number of reads mapped to the plastome; however, there was a clear effect of spiking on percentage of the plastome, with >4× coverage recovered (see Fig. 7G and H for percentage of the plastome recovered; see Appendix S16 for numbers of reads mapped to the plastome). According to the brms regression models, herbarium samples captured more of the plastome compared to silica‐dried material (for samples processed with the most recent probe kit versions) when group was taken into account in the brms regression model (Appendix S16). Fresh leaf material samples were also successful at capturing more of the plastome; these samples, however, were all processed using the oldest probe kit version, which was more successful at capturing the plastome compared to more recent versions (preliminary ANOVA; data not shown).

## DISCUSSION

### Asteraceae‐wide COS subsets are informative at multiple taxonomic levels

This study set out to test whether the “universal” COS Asteraceae family‐wide locus set (Mandel et al., 2014) is applicable for phylogenetic analyses at multiple evolutionary timescales (tribe‐ to species‐level). After potentially paralogous loci were removed from alignments in Table 1, alignments with PI characters were available for every sample group (Fig. 3; Appendices S9, S10). However, the proportion of COS loci that were parsimony informative varied among sample groups, notably between different taxonomic levels and between lineages at the same level. At the tribe level, 100% of exon and supercontig alignments were parsimony informative, whereas at the species and genus levels the proportion of exon alignments with PI characters ranged from 32% for *Sonchus* to 93% for *Lactuca* (calculated using AMAS; Fig. 3A, Appendix S10). Therefore, despite the relatively low number of loci remaining in the Vernonieae‐wide and Cichorieae‐wide non‐paralogous data sets compared to other sample groups, after filtering against paralogous loci and missing data (Fig. 2, Table 1), the potential for phylogenetic analyses is high with respect to percentage of PI sites.

In the species‐level exon alignments for *Carlina vulgaris*, proportions of PI sites ranged from 0.2–11% for ~30% of all alignments (Appendix S10); this illustrates the potential of the COS locus set for studies below the species level. An alternative method that is typically used for phylogenetic analyses at shallow taxonomic levels is restriction site–associated DNA sequencing (RAD‐Seq), because it results in greater total aligned sequence and more informative characters compared to hybrid capture studies (Harvey et al., 2016). However, RAD‐Seq is less repeatable and each locus is relatively short compared to Hyb‐Seq; furthermore, RAD‐Seq is prone to substantial amounts of missing data and homology is more difficult to assess. COS exon alignment lengths in this study ranged from 111–735 bp, ~5% of which were >500 bp, which is comparable to lengths of loci in other Hyb‐Seq studies (Appendix S10; e.g., Harvey et al., 2016). By building alignments of supercontigs (exon + flanking introns), average alignment length across all data sets was 997 bp, and more alignments contained markedly higher percentages of PI sites compared to the exon‐only alignments; we demonstrate this in Fig. 3B and C for the species complex level (*Picris hieracioides*) and genus level (*Lactuca*, *Chresta*, and *Lychnophora*). Despite the presence of shorter sequences among the exon alignments, subsets of non‐paralogous loci for different sample groups were phylogenetically informative, even at lower taxonomic levels (Appendix S9). Supercontig alignments were also informative for phylogenetic analyses across Cichorieae and for cluster networks of the *P. hieracioides* species complex; this will be discussed below. Therefore, Hyb‐Seq using the COS locus set generates reproducible data sets with relatively little missing data (all exon alignments contain 100% of samples with <70% missing data per locus, and supercontig alignments contain 80–100% samples with <75% missing data per locus; Appendices S9, S10) and provides sufficient information to resolve relationships at multiple evolutionary timescales.

### Levels of paralogy vary between tribes and paralogous loci show specificity to sample groups

Whole genome duplications have contributed to the evolution of the Asteraceae and have played a major role in the radiation of the family and its adaptation to a range of habitats (Barker et al., 2016; Huang et al., 2016). The prevalence of such events likely affects the numbers of paralogous loci in different lineages across the family. Barker et al. (2016 ) demonstrated a palaeotetraploid history at the base of the core Asteraceae (all lineages excluding Barnadesieae [Asteroideae–Mutisioideae]), and this was confirmed by Huang et al. (2016). Furthermore, previous studies have suggested that all tribes sampled in the present study have experienced more recent WGDs, with the exception of Cardueae, Vernonieae, and Moquinieae. Cichorieae and Heliantheae experienced WGD events in the early Eocene, Senecioneae in the early Miocene, and Gnaphalieae in the mid‐late Miocene (Huang et al., 2016). Cardueae, which is estimated to have originated sometime between the early‐late Eocene (54–34 mya; Panero and Crozier, 2016; Huang et al., 2016; Herrando‐Moraira et al., 2019; Mandel et al., 2019), is the oldest tribe in our sampling, and there has been no evidence of any WGDs for this tribe to date. This is consistent with our finding that it contains the lowest number of paralogous loci (267 in the Cardueae data set; Table 1) compared to all other tribes (Table 1). The average and maximum number of paralogous loci per sample in Cardueae was 140 and 166, respectively (Appendix S1). This is similar to what Herrando‐Moraira et al. (2018) reported with extensive sampling across Cardueae (>85 species) and an average of 144 paralogous COS loci per sample, according to HybPiper. When a locus is flagged as potentially paralogous in HybPiper, it may in fact indicate allelic variation. It was beyond the scope of this study to investigate this further. However, it would be recommended to explore this further by visually assessing the inferred topologies in gene trees with all copies of potentially paralogous loci for studies focused on lineages within Asteraceae (e.g., in Gardner et al., 2016; Johnson et al., 2016). Furthermore, Kates et al. (2018) provide a framework to infer phased alleles from target enrichment data to investigate allelic diversity in a Hyb‐Seq data set.

Tribe Vernonieae has a relatively high number of potentially paralogous loci (636 across the entire tribe, with 277 and 384 average and maximum paralogous loci per sample, respectively); however, to date no WGDs have been reported for this tribe. Vernonieae is one of the largest tribes in the Asteraceae, containing >1500 species, of which we sampled 26 from 15 different genera (Table 1). The high number of potentially paralogous loci flagged in Vernonieae may be due to lineage‐specific gene duplications within Vernonieae that remain to be discovered. In fact, among our sampled Vernonieae species, *Vernonia missurica* Raf., *V. gigantea* (Walter) Trel., *Vernoniastrum ambiguum* (Kotschy & Peyr.) H. Rob., and *Stokesia laevis* (Hill) Greene contained a markedly high number of loci (72) that are paralogous only in those species, suggesting that duplication events may be species‐specific. It may also be possible that duplications have occurred in other species but have diverged to such a degree that they are no longer recognized as paralogous. Paralog loss may also have occurred in certain groups of taxa, as was speculated for the Portullugo clade (Caryophyllales; Moore et al., 2018). In terms of genome size and chromosome number, Vernonieae is one of the least understood Asteraceae tribes (Garnatje et al., 2011; Vallès et al., 2013; Garcia et al., 2014), which hinders the indirect inference of gene duplications. Moore et al. (2018 ) showed that isolated duplications not confined to occasional WGD events are common along reconstructed branches in a phylogeny of the Portullugo clade (>2000 species). They also demonstrated that the targeted capture of genes that have undergone duplication events can be phylogenetically informative. Using statistical modeling and empirical data, Hellmuth et al. (2015) revealed that the distribution of gene duplications in gene families can in itself provide strong phylogenetic signal for resolving species relationships, and they concluded that it is not always necessary to restrict phylogenomic data sets to orthologous loci. Furthermore, Du et al. ( 2019a) showed that under an MSC model, analyses with paralogs is reliable. We therefore propose that further investigation into the phylogenetic signal of the potentially paralogous COS loci, as long as they can be accurately identified, would increase the utility and power of this family‐level Hyb‐Seq locus set, as was shown for *Artocarpus* (Gardner et al., 2016; Johnson et al., 2016).

Whole genome duplications are not unique to Asteraceae, and indeed it is likely that all angiosperms are descended from a paleopolyploid event (Jiao et al., 2011; but see Ruprecht et al., 2017). In addition, rampant WGDs have independently occurred across angiosperms, including within Brassicaceae, Fabaceae, Solanaceae, Poaceae, and Orchidaceae (Blanc and Wolfe, 2004; Vanneste et al., 2014). Moreover, WGDs have occurred at different times throughout history, with 25–35% of angiosperm species appearing to be recent polyploids (Wood et al., 2009). Therefore, Hyb‐Seq studies on any angiosperm lineage would need to deal with potentially paralogous loci. Our study shows that certain Asteraceae‐wide COS loci are non‐paralogous only in specific genera or tribes (Appendix S8). There are significant implications of recognizing the uniquely paralogous or non‐paralogous loci within different clades (genus or tribe) when sampling across broad taxonomic scales (and therefore across timescales). First, it allows for further investigation into the potential phylogenetic informativeness of paralogs. Lineage‐specific paralogous loci may be further investigated by extracting and analyzing all copies to understand the evolution of a lineage, as shown by Moore et al. (2018). Second, it allows a data set (in this case COS loci) to be subsampled to build locus data sets that are non‐paralogous for different clades (e.g., for genera within a tribe or family). These locus data sets would contain loci that are non‐paralogous and informative for some clades but that may be paralogous in others. This subsampling approach increases the power and applicability of Hyb‐Seq data sets to provide phylogenetic signal across broad taxonomic scales. The construction of locus data sets for the COS set and their use in different phylogenetic methods, as well as comparisons of the outcomes of those methods, are the focus of the next three sections.

### Tree estimation approaches influence node resolution in Cichorieae

A sister group relationship between subtribes Hyoseridinae (four *Sonchus* species) and Crepidinae (*Nabalus albus* and *Taraxacum kok‐saghyz*) was revealed in all analyses of our Cichorieae tribe‐wide data sets (Table 2; 24 samples). This is promising because the separation of subtribes Crepidinae and Lactucinae has proven complicated (see Kilian et al., 2017), yet Crepidinae is always sister to Hyoseridinae rather than to Lactucinae in our analyses (Fig. 4; Appendices S5, S13). Maximum likelihood analyses of the tribe‐supercontig data set (Fig. 4) resolved the subtribal backbone (for both partitioned and non‐partitioned data sets) that was unresolved in the ASTRAL tribe‐supercontig tree and in all analyses of the exon‐only alignments (Appendix S13). Further sampling within the Cichorieae subtribes will be necessary to further investigate the relationships observed here. Backbone relationships in tribe Cardueae were previously resolved using the COS locus set, in particular using a concatenation approach of exon‐only alignments with partitioning (Herrando‐Moraira et al., 2018, 2019).

Species tree approaches under the MSC model are often regarded as more accurate than concatenated approaches when analyzing multi‐locus sequence data (Heled and Drummond, 2010; Edwards et al., 2016). In concatenation approaches, high support can be observed for incorrect branches, and they may be statistically inconsistent in the presence of incomplete lineage sorting (Kubatko and Degnan, 2007; Roch and Steel, 2015). However, partitioning of concatenated data and including best‐fitting substitution models increases the reliability of this approach (Warnow, 2015), which we observe here for resolution within *Lactuca* and *Picris* in ML analyses of the tribe‐exon‐complete data set (Table 2, Appendix S5). This study therefore reveals not only the power of the COS locus set for phylogenetics at broad taxonomic levels but also the positive impact of data partitioning and model selection on ML concatenation approaches. This contradicts the results of a recent study suggesting that model selection may not be a mandatory step in phylogeny reconstruction (Abadi et al., 2019); however, see a critical response to that study by Gerth (2019).

### Resolution and levels of variation are influenced by the data subsampling approach

As expected, data sets of loci that are non‐paralogous for sample groups at shallow taxonomic levels (such as within a species complex) contain markedly more loci compared to data sets for a broad taxonomic group, such as a tribe (e.g., for Cichorieae; Table 2). Previous studies have shown that random subsampling of a set of loci and ordered subsampling (with increasing numbers of loci) can influence phylogenetic inferences (Simon et al., 2012; Bayzid and Warnow, 2013; Edwards, 2016). In this study, every genus contained uniquely non‐paralogous loci with respect to other genera in their tribes (Appendix S8); these loci have the potential to be informative for some clades but may be removed during tribe‐wide sampling. This highlights the potential benefit of using a locus subsampling strategy for large data sets that is guided by taxonomic level to maximize percentages of loci with PI sites. A recently developed supertree phylogeny estimation method (Molloy and Warnow, 2019) would enable the combination of phylogenies from different clades and would provide a powerful tool for such a locus subsampling strategy.

Clustering according to networks based on data sets containing non‐paralogous loci at the species complex level (and therefore more loci compared to at the tribe level; Table 2) revealed a distant position of *P. amalecitana* from all other *Picris* species, in accordance with previous studies of *Picris* (Appendix S14; Slovák et al., 2018). Supercontig alignments at the species complex level (Picris‐supercontig data set; Table 2) contained markedly more alignments with higher percentages of PI loci (Fig. 3B) and distances between samples were greater in the concatenated supercontig alignment, compared to exon‐only data sets and to the alignment of *Picris* from the tribe‐wide data set (Appendix S14). Therefore, analyses of the species complex–level sample group provide more informative regions compared to the tribe‐wide data set (with fewer loci) to disentangle relationships at shallow taxonomic levels. The power of the data set was further strengthened by generating supercontig alignments using HybPiper (Fig. 3B, Appendix S14).

High levels of conflict between gene trees are observed within the *Picris hieracioides* species complex according to phyparts, even when long branches were removed (Fig. 5, Appendix S15). This is likely due to the recent origin of the lineage and hybridization, which is consistent with recent studies that uncovered extensive gene flow both within and between species (Slovák et al., 2014, 2018).

### Long branches and incorporation of introns influence topological inferences

Previous phylogenetic studies of *Picris*, based on combined nrITS and plastid data, as well as comparisons of carpological characters, showed that *P. amalecitana* is distant from the *P. hieracioides* species complex (Slovák et al., 2012, 2014, 2018). In contrast, *P. amalecitana* is resolved within the species complex in the ML tree based on the tribe‐exon‐complete data set in our study (both with and without partitioning; Fig. 5A). However, by removing samples with long branches from exon alignments, the ML tree was consistent with all other analyses and previous studies (tribe‐exon‐shrunken data set; Table 2, Fig. 5B). Therefore, ML analyses of exon‐only alignments were influenced by long‐branch attraction (tribe‐exon‐complete tree data set vs. tribe‐exon‐shrunken data set; Fig. 5A vs. B, respectively). These contrasting relationships were also revealed by network and similarity matrices of the *Picris* alignments of loci from the 218 Cichorieae‐wide data set (tribe‐exon‐complete and tribe‐exon‐shrunken data sets; Fig. 6). The coalescent species tree approach at the tribe level (ASTRAL) alleviates the impact of long‐branch attraction, similar to a study within Cuppressaceae (Qu et al., 2017). Thus, the topological position of members of the *Picris hieracioides* species complex with respect to *P. amalecitana*, in the tribe‐exon ASTRAL trees, was unaffected by the removal of long branches and in accordance with previous studies (Appendix S13). Furthermore, 67% of all gene trees support the clade containing *P. amalecitana* outside of the *P. hieracioides* species complex in the tribe‐exon‐complete ASTRAL tree. *Picris* is a relatively recent (~5.23 mya) and rapidly evolving genus that has likely accumulated multiple mutations, which tend to be saturated, and this likely contributes to the impact of long‐branch attraction in ML analyses (Pisani, 2004). Villaverde et al. (2018 ) also detected the impact of long‐branch attraction on topologies inferred in phylogenetic analyses of concatenated data sets of *Euphorbia balsamifera* by sequentially removing samples and re‐estimating the ML tree using IQ‐Tree. The percentages of gene trees supporting the clade containing *P. amalecitana* in ML analyses of exon data sets show a clear increase when long branches are removed; from only 8% in the tribe‐exon‐complete tree to 67% in the tribe‐exon‐shrunken tree (Fig. 5A, B). Therefore, removing samples with long branches helps to increase the reliability of topological inferences at the shallower taxonomic levels in ML analyses. Furthermore, by generating alignments containing both flanking intron regions and exons, the tribe‐wide concatenated alignment length increased more than threefold (Table 2) and the support for the *Picris* clade (with *P. amalecitana* outside of the species complex) increased even more (to 84%), compared to exon‐only alignments (tribe‐supercontig data set; Fig. 5C). We therefore reveal the potential of supercontig alignments when taxon sampling is broad (tribe‐wide) to infer relationships within rapidly evolving lineages at shallow taxonomic levels (Fig. 4).

### Factors affecting number of reads mapping to targets and off‐target plastome

Previous studies have suggested that a number of factors may influence the capture of targets in Hyb‐Seq (Hart et al., 2016; Villaverde et al., 2018; Johnson et al., 2019) and numbers of reads mapping to plastomes (Bakker et al., 2016). As would be expected, HiSeq generates more reads than other sequencing platforms in our study (MiSeq and NextSeq; Appendix S1, Fig. 7A); see also Wolf et al. (2018). Correlation tests showed that total read number was positively correlated with number of reads mapped to targets and with number of targets recovered (Appendix S6), similar to Johnson et al. (2019). Therefore, we aimed to investigate the factors that influence number of reads mapped to targets and the off‐target plastome. Samples in group 3 had higher numbers of reads mapped to targets compared to other groups, as would be expected as they were processed using the most recent probe kit version (version 3) in combination with a HiSeq 3000 sequencing platform; 96 samples were included in a single sequencing lane for samples in this group (Fig. 7A, Appendix S1). It is notable that, for samples processed with the most recent probe kit (version 3) and sequenced using MiSeq, on average more reads mapped to targets when hybrid capture pool size and incubation time were 24 samples and 36 h, respectively, compared to fewer than four samples and 26 h, respectively (group 8 vs. 9; average: 352,987 vs. 740,290 reads mapped, respectively; Fig. 7A, Table 3). Therefore, a markedly higher hybrid capture pool size and 10‐h shorter incubation time did not show a detrimental impact on number of reads mapping to targets. Brms analyses across the entire data set suggested that the number of reads mapped to targets decreased for larger genomes (genome sizes in this data set range from 0.57–16.25 1C picograms [1C pg] and average genome size is 2.23 1C pg; Fig. 7B, Appendix S1). Indeed, the sample with the highest genome size in this study (*Stokesia laevis* (Hill) Greene; 16.25 1C pg) had the lowest number of reads mapped to targets compared to all other samples in group 2 (362,713 [1.92%] mapped reads, group 2; Table 3). Wolf et al. (2018 ) found no clear effect of genome size on mapping to targets in ferns, but with fewer samples than this study. Therefore, it would be beneficial for future Hyb‐Seq studies to report genome sizes and number of reads mapping to targets to gain a clearer consensus of the impact of genome size on the performance of Hyb‐Seq protocols. Indeed, it would be useful to have more genome size estimates available. In this study, estimations were only available for 34 samples; remaining genome sizes were based on averages for the taxonomic group. Our brms analysis suggested that silica‐dried samples have only a slight positive effect on number of reads mapped to targets compared to herbarium samples (Appendices S1, S16). Villaverde et al. (2018) showed that capture success (summed captured length divided by the summed mean reference length) was markedly higher for silica‐dried compared to herbarium material for *Euphorbia balsamifera*. Overall, our study shows that the Hyb‐Seq approach is relatively flexible using the COS locus set; a range of lab steps were applied and, of the 1061 genes targeted, >702 were captured with >70% of the reference length according to HybPhyloMaker (mean: 954, highest value: 1055 target loci; Appendix S1, Table 3).

When samples were sequenced using HiSeq3000 or NextSeq, more targets reached >75% of the reference length compared to using MiSeq; an average of 660 (587–698) and 574 (127–698) targets reached >75% of the reference length according to HybPiper after HiSeq3000 or NextSeq compared to MiSeq sequencing (based on samples processed with the most recent probe set; Appendix S1). Therefore, HiSeq3000 or NextSeq sequencing platforms maximized the target length, which increased the potential for capturing flanking intron regions. This is reflected by the fact that more supercontig alignments with >80% samples survived trimming of spurious sequences and gap removal when samples had more loci that were >75% of the reference length according to HybPiper. For example, samples in Cichorieae and Gnaphalieae had on average 635 and 380 loci with >75% of the reference length, and 166 and 64 supercontig alignments with >80% samples survived trimming (see Appendices S1, S9).

This study highlights the significance of library spiking for increasing the percentage of the plastome recovered (>4×) when using the most recent version of the probe kit (Fig. 7G, H). When samples were spiked, >94% of the plastome was recovered for 90% of the 31 that were spiked (Appendix S1). Among all samples that were spiked, just two recovered <70% of the plastome; they were also the only two samples that were not sheared prior to library preparation (33.65% for *Pericallis papyracea* (DC.) B. Nord. and 69.1% for *P. webbii* (Sch. Bip.) Bolle). This may suggest that the DNA was too degraded for plastome recovery or that shearing DNA in addition to sample spiking facilitates plastome recovery; further sampling would be necessary to understand this fully. Of the 40 samples that were not spiked and processed using the most recent probe kit, ~30% had <50% plastome recovery (Appendix S1, Fig. 7G). The following library spiking approaches were used in the present study: 40% or 33% unenriched with 60% or 66% enriched library (Appendix S2). Similar to number of reads mapping to targets, increased genome size showed a slight negative impact on number of reads mapping to the plastome according to the brms regression models. A previous study investigating plastome sequence assembly of herbarium specimens showed no significant impact of *C* values on plastome capture (Bakker et al., 2016). When the variation in percentage of plastome recovered among groups listed in Table 3 was taken into account, higher proportions of the off‐target plastome were recovered for herbarium samples than for silica‐dried samples (with >4× coverage; according to the brms regression model in Appendix S16). Therefore, in support of previous studies, we reveal the potential for herbarium specimens for next‐generation sequencing and plastome capture (Staats et al., 2013; Bakker et al., 2016; Hart et al., 2016).

## CONCLUSIONS

The COS Asteraceae family‐wide 1061 locus Hyb‐Seq probe set is parsimony informative at multiple taxonomic levels (tribe to species). It is therefore a powerful tool for phylogenetic analyses in systematic and evolutionary studies across the family. This study reveals that there are genus‐specific non‐paralogous COS loci with respect to other genera in the same tribe. Analyses of different non‐paralogous locus data sets (species complex level vs. tribe level) sampled from the targeted COS locus set lead to contrasting topological inferences at shallower timescales. Furthermore, we show the impact of long branches as a potential source of conflict between ASTRAL and RAxML‐NG species trees, which can be alleviated by removing long branches. Hyb‐Seq probe set design therefore does not necessarily need to be lineage‐specific for shallow taxonomic levels; rather, how the locus set is subsampled and analyzed is important for resolution and inferred topologies. These findings have implications for angiosperm phylogenetics using Hyb‐Seq, especially as universal probe kits are becoming available (Buddenhagen et al., 2016; Johnson et al., 2019).

This study also reveals the broad applicability of Hyb‐Seq when a range of lab steps are used, and we provide the wet‐lab workflows used in the three labs included in this study (Appendix S2). Number of reads mapping to targets increased when samples were sequenced using HiSeq (also when the number of samples in a sequencing lane was 96). The number of reads mapping to targets was not negatively affected when more samples were pooled in a hybrid capture reaction (24 vs. <4) and sample incubation time was shorter (24 vs. 36 h). We show that library spiking was important for obtaining maximum plastome completeness (with >4× coverage). More Hyb‐Seq probe kits are being applied to evolutionary studies across angiosperms. Therefore, it would be highly beneficial for researchers to provide more information regarding read mapping and locus capture success in combination with lab steps as supplemental data. This not only would help novices with the development of this method in their research laboratories but also would lead to a stronger overview of the processes that can improve the efficiency of target capture.

## AUTHOR CONTRIBUTIONS

The initial idea and planning were carried out by discussions between K.E.J., R.E.S., T.F., and J.R.M. J.R.M. built the collaboration to make this work possible, making the Hyb‐Seq data and sample information available for analyses. T.F. and R.E.S. also provided a significant amount of Hyb‐Seq data and sample information, including unpublished genome size data from Charles University, Prague, Czech Republic. M.S. contributed important knowledge and data for *Picris*, and N.K. for Cichorieae. T.F. conducted HybPhyloMaker analyses. K.E.J. conducted analyses in HybPiper, phylogenetic analyses within Cichorieae, and regression analyses. K.E.J. planned and wrote the manuscript; discussions with and edits from J.R.M., T.F., L.E.W., N.K., and R.E.S. contributed to this. All other co‐authors (R.B.D., N.K., V.A.F., S.H.‐M., C.M.S., A.S., P.R.J., and R.T.) made important contributions, in particular regarding data availability and accessibility, project planning, and/or ideas for data presentation. All authors read and commented on the manuscript.

## Supporting information


**APPENDIX S1.** Sequence Read Archive and National Center for Biotechology Information sample accession numbers or reference for data accessibility.

**APPENDIX S2.** Wet‐lab workflow for hybrid capture of the mybaits conserved orthologous set used in three different labs for samples in this paper (Berlin Botanic Garden, Charles University Prague, and University of Memphis), including the workflow conducted at Barcelona Botanic Garden.

**APPENDIX S3.** Comparison of HybPiper and HybPhyloMaker mapping results to the conserved orthologous set target references across all samples in Appendix 1.

**APPENDIX S4.** Preliminary assessment of two trimming approaches of supercontig (exon + flanking intron region) alignments (the splash‐zone) using trimAl.

**APPENDIX S5.** Phylogenetic analyses of the Cichorieae tribe‐exon‐complete data set (218 loci) revealed inconsistencies in topological inferences for *Picris amalecitana* between maximum likelihood and coalescent species tree (ASTRAL) analyses.

**APPENDIX S6.** Corrected Pearson correlation coefficients with *P* values between the number of sequenced reads, reads mapped to targets (number and percentage), off‐target plastome (number and percentage capture), number of targets mapped, and number of targets with sequences.

**APPENDIX S7.** Numbers of targeted loci removed at each stage of data cleaning of exon alignments in HybPhyloMaker for each sample (see also pipeline in Fig. 2).

**APPENDIX S8.** Area‐proportional Venn diagrams for each tribe illustrating the proportions of non‐paralogous loci that are unique to each genus, species complex, or species sampled.

**APPENDIX S9.** Summary of numbers of samples in supercontig (exon + intron) alignments before and after data trimming and raw data of supercontig alignment summaries for all sample groups in Table 1.

**APPENDIX S10.** Exon alignment summary statistics (non‐paralogous loci) for all sample groups in Table 1.

**APPENDIX S11.** Variable and parsimony informative sites in concatenated exon alignments at multiple taxonomic levels within tribes Vernonieae, Senecioneae, Moquinieae, Heliantheae, and Gnaphalieae.

**APPENDIX S12.** RAxML‐NG likelihood scores of analyses with and without partitioning and different branch linkage models for the Cichorieae tribe‐wide data sets.

**APPENDIX S13.** ASTRAL species tree and RAxML‐NG trees with and without partitioning based on the Tribe‐exon‐shrunken data set and ASTRAL species tree of the Tribe‐supercontig‐complete data set. Scale bars indicate the expected number of nucleotide substitutions per site.

**APPENDIX S14.** *Picris hieracioides* species complex similarity matrices based on the Picris‐610‐exon‐complete and ‐shrunken and Picris‐supercontig data sets, and networks based on the Picris‐610‐exon‐complete and ‐shrunken data sets (see data set information in Table 2).

**APPENDIX S15.** Summaries of conflicting and concordant gene trees from phyparts with the tribe‐exon‐complete ASTRAL species trees and supercontig maximum likelihood (ML) and ASTRAL trees.

**APPENDIX S16.** Boxplots and regression models (using Bayesian regression analyses) to explore the impact of sample type (herbarium and silica‐dried material) on number of reads mapped to targets and the off‐target plastome.

## Data Availability

Raw sequence data for 42 of the 112 samples in this study are available in .fastq format in the National Center for Biotechnology Information's Sequence Read Archive (BioProject PRJNA516161). Data for 47 samples were published in Mandel et al. (2019). Due to ongoing manuscript preparation, raw data for the remaining 24 samples are embargoed until other manuscripts are accepted; details of authorship for these papers, along with respective samples, are given in Appendix S1. These embargoed raw data can be made available on request. All alignments and files for Cichorieae data analyses (see Tables 1 and 2) are made available on Dryad (Jones et al., 2019).

## APPENDIX 1 Tribe and voucher information for samples used in this study.

**Table:**

Tribe	Species and authority	Collector name, number (Herbarium)	Collection date	Collection locality
Cardueae	*Carlina vulgaris* L.	H. Mašková (PRC)	s.d.	Czech Republic: Prague
Cardueae	*Carlina vulgaris* L.	Z. Kaplan, H. Mašková (PRC)	s.d.	Czech Republic: Hodonín
Cardueae	*Carlina vulgaris* L.	Z. Kaplan (PRC)	s.d.	Slovak Republic: Ružomberok
Cardueae	*Carlina vulgaris* L.	Z. Kaplan (PRC)	s.d.	Czech Republic: Břeclav
Cardueae	*Carlina vulgaris* L.	F. Kolář (PRC)	s.d.	Sweden: Kalmar
Cardueae	*Carlina vulgaris* L.	H. Mašková (PRC)	s.d.	Czech Republic: Znojmo
Cardueae	*Cousinia albertoregelia* C. Winkl.	V. Botschantzev 166 (LE)	14 May 1975	Tadjikistan: Tujuntau mountains
Cardueae	*Cousinia macroptera* C. A. Mey.	Tamanian (ERE)	11 June 2004	Armenia: Ararat province, Ashtarak district
Cardueae	*Cousinia pusilla* C. Winkl.	V. Botschantzev 117 (LE)	s.d.	Tajikistan: S Tajikistan
Cardueae	*Cousinia spryginii* Kult.	V. Botschantzev 46 (LE)	9 May 1975	Uzbekistan: Kashkadarbinskaya region
Cardueae	*Carthamus tinctorius* L.	n/a	n/a	Greenhouse‐grown seed, USDA, PI 592391
Cardueae	*Cynara cardunculus* L.	J. R. Mandel 135 (GA)	24 Sep. 2014	Greenhouse‐grown seed, collected UW Medicinal Plant Garden
Cardueae	*Echinops strigosus* L.	L. E. Watson 95‐7A (MU)	July 1995	Spain: Andalucia
Cardueae	*Cousinia strobilocephala* Tscherneva & Vved.	R. Aydarova & O. Chypaev (FRU)	6 July 1980	Kyrgyzstan: Kirghizia, Qurama Range, Kayyndy‐Say River
Cichorieae	*Gundelia tournefortii* L.	al‐Hosseini s.n. (US)	—	Iran
Cichorieae	*Leontodon tingitanus* Ball	L. E. Watson 95‐33A (MU)	July 1995	Spain: Andalucia
Cichorieae	*Nabalus albus* (L.) Hook.	Schilling, E. 3225 (TENN)	—	USA: Campbell Co., TN
Cichorieae	*Taraxacum kok‐saghyz* L. E. Rodin	J. R. Mandel 102 (GA)	27 Aug. 2013	Greenhouse‐grown seed, USDA, W6 35156
Cichorieae	*Tragopogon dubius* Scop.	Scop. (WS)	s.d.	USA: Oakesdale, WA
Cichorieae^a^	*Hieracium alpinum* L.	P. Mráz, J. Chrtek, J. Košút ALP10/5 (PRC)	s.d.	Italy: Passo di Tonale
Cichorieae^a^	*Hieracium alpinum* L.	P. Mráz, J. Košút ALP2/2 (PRC)	s.d.	Switzerland: Col du Grand St. Bernard
Cichorieae^a^	*Hieracium alpinum* L.	P. Mráz, P. Turis ALP31/4 (PRC)	s.d.	Slovakia: Mt. Chohuľa
Cichorieae^a^	*Hieracium alpinum* L.	M. Puşcaş ALP59/4 (PRC)	s.d.	Romania: Mt. Pietrosul Călimanulu
Cichorieae^a^	*Hieracium alpinum* L.	J. Chrtek ALP85/1 (PRC)	s.d.	Czech Republic: Mt. Praděd
Cichorieae^a^	*Hieracium alpinum* L.	P. Mráz, R. Mráz ALP93/10 (PRC)	s.d.	Norway: Haukelitunnelen
Cichorieae	*Lactuca orientalis* Boiss.	Weber s.n. (B)	30 Sep. 1998	Iran: Isphahan
Cichorieae	*Lactuca palmensis* Bolle	M. Cubr 35816 (B)	4 Jul. 1997	Switzerland: Valais, cult. BG Berlin‐Dahlem Acc. 137‐02‐89‐14
Cichorieae	*Lactuca perennis* L.	M. Ristow, D. Lauterbach & B. Gemeinholzer MiRi 578/09 (B)	12 July 2009	Italy: Piemont. Cuneo
Cichorieae	*Lactuca quercina* L.	C. Oberprieler 10168 (B)	27 June 2002	Armenia: Vayotsdzor province
Cichorieae	*Lactuca serriola* L.	Coskuncelebi & Güzel 141 (KTUB)	10 Sep. 2013	Turkey: Artvin: Kafkasör'e çıkarken, 1072 m
Cichorieae	*Lactuca tatarica* (L.) C. A. Mey.	M. Ristow 718/08 (B)	22 June 2008	Germany: Brandenburg
Cichorieae	*Picris amalecitana* (Boiss.) Eig	Marek Slovák & Jaromír Kučera IZ3/6 (SAV)	3 Apr. 2012	Israel: Center district, near Michmoret village, Alexander river park, 4 m, 32°23′43″N, 34°52′20″E
Cichorieae	*Picris hieracioides* L. subsp. *hieracioides*	Marek Slovák & Jaromír Kučera CZ1/3 (SAV)	21 Sep. 2011	Czech Republic: Břeclav, 166 m, 48°46′51″N, 16°54′19″E
Cichorieae	*Picris hieracioides* subsp. *hispidissima* (Bartl.) Slovák & Kučera	Marek Slovák, Jaromír Kučera & A. Guttová LOV1 (SAV)	13 June 2012	Montenegro: Danilovgrad, between the villages Kujava and Cerovo
Cichorieae	*Picris hieracioides* subsp. *umbellata* (Schrank) Ces.	Marek Slovák & Judita Zozomová‐Lihová MAD4 (SAV)	21 Aug. 2003	Slovakia: Západné Tatry Mts., Mačie diery
Cichorieae	*Picris hieracioides* subsp. hispidissima (Bartl.) Slovák & Kučera	Marek Slovák, Jaromír Kučera & A. Guttová PAK2 (SAV)	11 June 2012	Croatia: Ličko‐senjska županija, Velika Paklenica valley
Cichorieae	*Picris hieracioides* subsp. *hieracioides* L.	Marek Slovák PESK1 (SAV)	21 June 2004	Italy: Abruzzi, Pescara, 6 m, 42°27′29″N, 14°12′36″E
Cichorieae	*Picris japonica* Thunb.	Karol Marhold & Judita Zozomová‐Lihová JP106/1 (SAV)	29 June 2004	Japan: Akita pref., Kitaakita‐gun, Tashiro‐cho, Hirataki, 339 m, 40°22′23″N, 140°26′20″E
Cichorieae	*Picris nuristanica* Bornm.	N.A., NUR7 (SAV)	N.A.	Kirgiyzia: Fergana Kyrka Toosu, Mts., 2800 m, 40°52′29″N, 74°04′59″E
Cichorieae	*Picris olympica* Boiss.	Jaromír Kučera, Marek Slovák & A. Guttová UD9 (SAV)	15 June 2010	Turkey: Bursa province, Uludağ Mts., 2059 m, 40°05′35″N, 29°07′52″E
Cichorieae	*Sonchus radicatus* Aiton	Jan Suda (PRC)	2004	Spain: Tenerife
Cichorieae	*Sonchus tuberifer* Svent.	Jan Suda (PRC)	2004	Spain: Tenerife
Cichorieae	*Sonchus ustulatus* Lowe subsp. *maderensis* Aldridge	Jan Suda (PRC)	2004	Portugal: Madeira
Cichorieae	*Sonchus pinnatus* Aiton	Jan Suda (PRC)	2004	Spain: Tenerife
Gnaphalieae	*Antennaria anaphaloides* Rydb.	R. J. Bayer, Purdy & Newby MT‐92005 (ALTA)	12 June 92	USA: Montana, Choteau Co., 47.47 −110.53
Gnaphalieae	*Antennaria corymbosa* E. E. Nelson	Bayer and Lebedyk M‐508 (ALTA)	27 July 85	USA: Montana, Beaverhead Co., 1951 m, 45.23 −111.45
Gnaphalieae	*Antennaria flagellaris* (A. Gray) A. Gray	R. J. Bayer, Minish, & Francis OR‐91006 (ALTA)	s.d.	USA: Oregon, Crook Co., Ochocho Mountains 47.47 −110.53
Gnaphalieae	*Antennaria geyeri* A. Gray	R. J. Bayer, Minish, and Francis OR‐91008 (ALTA)	4 June 1991	USA: Oregon, Deschutes County 44.3 −121.58
Gnaphalieae	*Disparago* sp. Gaertn.	V. A. Funk et al. 12985 (US)	15 Jan. 2014	South Africa: Western Cape
Gnaphalieae	*Facelis lasiocarpa* (Griseb.) Cabrera	R. J. Bayer & Chandler ARG‐02049 (CANB)	20 Jan. 2002	Argentina: Mendoza Province, Tunuyan, Andes Range
Gnaphalieae	*Gamochaetopsis alpina* (Poepp.) Anderb. & S. E. Freire	Bayer R. J. & Chandler ARG‐02080 (CANB)	26 Jan. 2002	Argentina: Tierra del Fuego, Isla Grande, Garibaldi Pass
Gnaphalieae	*Luciliocline subspicata* (Wedd.) Anderb. & S. E. Freire	R. J. Bayer & Chandler ARG‐02029A (CANB)	15 Jan. 2002	Argentina: Jujuy Province, Yavi
Gnaphalieae	*Oedera squarrosa* (L.) Anderb. & K. Bremer	Watson, L. E. & Panero, J. 94‐28 (MU)	18 Nov. 1994	South Africa: Western Cape
Gnaphalieae	*Pseudognaphalium obtusifolium* (L.) Hilliard & B. L. Burtt	Funk, V. A. 12773 (US)	12 Sep. 2011	USA: Falls Church, VA
Gnaphalieae	*Syncarpha* sp. DC.	V. A. Funk et al. 12987 (US)	15 Jan. 2014	South Africa: Western Cape
Heliantheae	*Helianthus annuus* L.	n/a	n/a	Greenhouse‐grown seed, USDA, PI 603989
Heliantheae	*Helianthus argophyllus* Torr. & A. Gray	n/a	n/a	Voucher n/a, USDA, PI 435623
Heliantheae	*Helianthus porteri* (A. Gray) Pruski	J. R. Mandel 103 (GA)	22 Oct. 2013	USA: DeKalb Co., Georgia, greenhouse‐grown seed collected
Heliantheae	*Helianthus verticillatus* Small	J. R. Mandel 101 (GA)	1 Sep. 2004	USA: Madison Co., Tennesee, greenhouse‐grown seed collected
Heliantheae	*Lipochaeta lobata* (Gaudich.) DC.	S. Keeley s.n. (US)	1 Mar. 1993	USA: HI, Hanaula Rd., Maui
Heliantheae	*Lipochaeta micrantha* (Nutt.) A. Gray	T. Flynn 735 (PTBG)	12 Jan. 1984	Hawaii: Kauai
Heliantheae	*Lipochaeta subcordata* A. Gray	J. Davis 299 (US)	7 June 1987	USA: Pōhakuloa Training Area btw. Mauna Loa, Mauna Kea
Heliantheae	*Montanoa tomentosa* Cerv.	Velasco & Funk 5819 (US)	29 Aug. 2014	Mexico: Oaxaca
Heliantheae	*Phoebanthus tenuifolius* (Torr. & A. Gray) S. F. Blake	C. M. Mason 101 (GA)	10 Sep. 2010	USA: Florida, Liberty County, greenhouse‐grown seed collected
Heliantheae	*Rojasianthe superba* Standl. & Steyerm.	Funk, V. A. 13328 (US)	30 Apr. 2016	San Francisco Botanical Garden‐Cloud Forest
Heliantheae	*Tithonia rotundifolia* (Mill.) S. F. Blake	J. R. Mandel 116 (MEM)	19 Mar. 2014	Greenhouse‐grown seed, USDA, PI 545684
Heliantheae	*Verbesina alternifolia* (L.) Britton ex Kearney	Mandel, J. R. 133 (GA)	24 Sep. 2014	Greenhouse‐grown seed, collected UW Medicinal Plant Garden
Heliantheae	*Wollastonia biflora* (L.) DC.	K. Woolliams 165 (PTGB)	22 July 1973	National Tropical Botanical Garden, Okinawa Island, grown in NTBG garden
Moquinieae	*Moquinia racemosa* (Spreng.) DC.	C. M. Siniscalchi 536 (SPF)	2 Sep. 2014	Brazil: Minas Gerais, Diamantina
Moquinieae	*Pseudostifftia kingii* H. Rob.	N. Roque 4490 (US)	28 Oct. 2014	Brazil: Bahia
Senecioneae	*Brachyglottis repanda* J. R. Forst. & G. Forst.	K. Ford 45/91	6 Nov. 1991	New Zealand: Marlborough, Wakamarina riverbed
Senecioneae	*Senecio flavus* (Decne.) Sch. Bip.	V. A. Funk 12774	7 Nov. 2011	USA: Washington, D.C., National Museum of Natural History
Senecioneae	*Werneria aretioides* Wedd.	V. A. Funk et al. 13109 (US)	7 Mar. 2014	Chile: Arica
Senecioneae	*Xenophyllum lycopodioides* (S. F. Blake) V. A. Funk	V. A. Funk et al. 13103 (US)	7 Mar. 2014	Chile: Arica
Senecioneae	*Pericallis aurita* (L'Hér.) B. Nord.	Jan Suda (PRC)	s.d.	Portugal: Madeira
Senecioneae	*Pericallis echinata* (L. f.) B. Nord.	Jan Suda (PRC)	s.d.	Spain: Canary Islands, Tenerife
Senecioneae	*Pericallis lanata* (L'Hér.) B. Nord.	Jan Suda (PRC)	s.d.	Spain: Canary Islands, Tenerife
Senecioneae	*Pericallis murrayi* (Bornm.) B. Nord	Jan Suda (PRC)	s.d.	Spain: Canary Islands, El Hierro
Senecioneae	*Pericallis papyracea* (DC.) B. Nord.	Jan Suda (PRC)	s.d.	Spain: Canary Islands, La Palma
Senecioneae	*Pericallis webbii* (Sch. Bip.) Bolle	Jan Suda (PRC)	s.d.	Spain: Canary Islands, Gran Canaria
Senecioneae	*Senecio canescens* (Bonpl.) Cuatrec. [in BioProject as *Culticum canescens*]	P. Sklenář, E. Dušková 12356 (PRC)	s.d.	Colombia: Boyaca
Senecioneae	*Senecio involucratus* (Kunth) DC.	P. Sklenář, J. Karbulková 11116 (PRC)	s.d.	Ecuador: Azuay
Senecioneae	*Senecio lingulatus* Franch.	P. Sklenář, E. Rejzková, F. Kolář 11538 (PRC)	s.d.	Ecuador: Imbabura
Senecioneae	*Senecio nivalis* (Kunth) Cuatrec.	P. Sklenář, J. Macková 11580 (PRC)	s.d.	Ecuador: Napo
Senecioneae	*Senecio patens* (Kunth) DC.	P. Sklenář, E. Rejzková, F. Kolář 11565 (PRC)	s.d.	Ecuador: Cotopaxi
Senecioneae	*Senecio subinvolucratus* Cuatrec.	P. Sklenář, A. Kučerová A., P. Macek 11076 (PRC)	s.d.	Ecuador: Pichincha
Vernonieae	*Allocephalus gamolepis* Bringel, J. N. Nakaj. & H. Rob.	J. Bringel 416 (CEN)	31 Mar. 2008	Brazil: Goiás, Guarani de Goiás
Vernonieae	*Centrapalus pauciflorus* (Willd.) H. Rob.	Mandel, J. R. 104 (GA)	22 Oct. 2013	Greenhouse‐grown seed, USDA, PI 312852
Vernonieae	*Chronopappus bifrons* (DC. ex Pers.) DC.	B. Loeuille 465 (SPF)	28 Jan. 2009	Brazil: Minas Gerais, Santo Antônio do Itambé
Vernonieae	*Hololepis pedunculata* (DC. ex Pers.) DC.	C. M. Siniscalchi 588 (SPF)	26 May 2015	Brazil: Minas Gerais, Rio Acima
Vernonieae	*Lepidaploa opposita* A. M. Teles, Sobral & J. N. Nakaj.	C. M. Siniscalchi 508 (SPF)	3 Aug. 2014	Brazil: Minas Gerais, Alvarenga
Vernonieae	*Minasia pereirae* H. Rob.	B. Loeuille 862 (SPF)	14 July 2013	Brazil: Minas Gerais, Santana do Riacho
Vernonieae	*Paralychnophora harleyi* (H. Rob.) D. J. N. Hind	S. C. Ferreira (HUEFS)	19 May 2007	Brazil: Bahia
Vernonieae	*Piptolepis ericoides* (Lam.) Sch. Bip.	B. Loeuille 866 (SPF)	14 July 2013	Brazil: Minas Gerais, Santana do Riacho
Vernonieae	*Stilpnopappus tomentosus* Mart. ex DC.	C. M. Siniscalchi 408 (SPF)	20 Nov. 2013	Brazil: Bahia, Rio de Contas
Vernonieae	*Stokesia laevis* (Hill) Greene	C. M. Siniscalchi 645 (SPF)	4 June 2015	USA: Louisiana, New Orleans cultivated
Vernonieae	*Vernonanthura patens* (Kunth) H. Rob.	S. Keeley & J. Keeley 3297 (US)	16 June 1980	Guatemala: Chimaltenango
Vernonieae	*Vernonia gigantea* (Walter) Trel. ex Branner & Coville	S. Keeley s.n. (K)	17 Oct. 1993	U.S.A.: Kew Garden accession 611‐66‐61103
Vernonieae	*Vernonia missurica* Raf.	L. Urbatsch 5870 (LSU)	17 Aug. 1989	U.S.A.: Louisiana, La Salle, 11.8 miles NW of the LA 500 jct with LA 84 W of Jena
Vernonieae	*Vernoniastrum ambiguum* (Kotschy & Peyr.) H. Rob.	J. M. Fay 5944 (MO)	17 Oct. 1983	Central African Republic: Bamingui‐Bangoran from seed
Vernonieae	*Chresta angustifolia* Gardner	C. M. Siniscalchi 490 (SPF)	14 July 2014	Brazil: Goiás, Alto Cavalcante
Vernonieae	*Chresta curumbensis* (Philipson) H. Rob.	C. M. Siniscalchi 573 (SPF)	10 Mar. 2015	Brazil: Planaltina, Distrito Federal
Vernonieae	*Chresta harleyi* H. Rob.	C. M. Siniscalchi 459 (SPF)	1 May 2014	Brazil: Bahia, Licínio de Almeida
Vernonieae	*Chresta pacourinoides* (Mart. ex DC.) Siniscalchi & Loeuille	B. Loeuille 351 (SPF)	22 Sep. 2007	Brazil: Bahia, Feira de Santana
Vernonieae	*Chresta plantaginifolia* (Less.) Gardner	C. M. Siniscalchi 573 (SPF)	3 Dec. 2014	Brazil: Distrito Federal, Gama
Vernonieae	*Chresta sphaerocephala* DC.	C. M. Siniscalchi 576 (SPF)	10 Mar. 2015	Brazil: Distrito Federal, Planaltina
Vernonieae	*Lychnophora brunioides* Mart.	B. Loeuille 466 (SPF)	17 Dec. 2008	Brazil: Minas Gerais, Santo Antônio do Itambé
Vernonieae	*Lychnophora granmogolensis* (Duarte) D. J. N. Hind	C. M. Siniscalchi 348 (SPF)	1 May 2013	Brazil: Bahia, Ibicoara
Vernonieae	*Lychnophora haplopappa* sp. ined.	B. Loeuille 902 (SPF)	3 Aug. 2014	Brazil: Minas Gerais, Alvarenga
Vernonieae	*Lychnophora hatschbachii* (H. Rob.) Loeuille, Semir & Pirani	B. Loeuille 613 (SPF)	25 Apr. 2012	Brazil: Minas Gerais, Diamantina
Vernonieae	*Lychnophora morii* H. Rob.	B. Loeuille 658 (SPF)	25 May 2012	Brazil: Bahia, Palmeiras
Vernonieae	*Lychnophora santosii* H. Rob.	B. Loeuille 669 (SPF)	26 May 2012	Brazil: Bahia, Rio de Contas

a Not included in Cichorieae phylogenetic analyses.
