# Supplementary material for: An empirical assessment of a single family‐wide hybrid capture locus set at multiple evolutionary timescales in Asteraceae
Source: Appl Plant Sci. 2019 Oct 25;7(10):e11295. doi: 10.1002/aps3.11295 (PMC6814182; doi:10.1002/aps3.11295)
Supplement: Supplementary file 2 — APPENDIX S2. Wet‐lab workflow for hybrid capture of the mybaits conserved orthologous set used in three different labs for samples in this paper (Berlin Botanic Garden, Charles University Prague, and University of Memphis), including the workflow conducted at Barcelona Botanic Garden. [file APS3-7-e11295-s002.docx]

**APPENDIX S2.** Wet-lab workflow for hybrid capture of the mybaits conserved orthologous set used in three different labs for samples in this paper (Berlin Botanic Garden, Charles University Prague, and University of Memphis), including the workflow conducted at Barcelona Botanic Garden. Refer to Appendix S1 for details for each sample. The lab name for each sample is also provided in Appendix S1.

**Overview of lab workflow**

STEP 1: DNA extraction

STEP 6: Pool libraries ready for hybrid capture

STEP 5: Check concentration

STEP 4: Library preparation

STEP 3: DNA fragmentation (optional, see below)

STEP 2: Check gDNA fragment size and concentration.

STEP 7: Hybrid capture and amplification of enriched library

STEP 8: Equimolar pooling of libraries, quality assessment, and high-throughput sequencing

(optional: pool pre-capture and post-capture libraries)

**Hyb-Seq lab workflow step-by-step**

| **STEP** | **Berlin** | **Memphis** | **Barcelona** | **Prague** |
| --- | --- | --- | --- | --- |
| **1: DNA extraction** | *Kits and protocols:* QIAGEN DNeasy Plant Mini Kit  NucleoSpin Plant II Macherey Nagel  (NB: These protocols were adapted so that ground up leaf samples were incubated overnight in lysis buffer prior to adding RNase, this increases.)  *Tissue type, weight:* Herbarium sample, 10–20 mg | *Kits and protocols:* Omega Biotek E.Z.N.A SQ DNA, QIAGEN DNeasy Plant Mini Kit  *Tissue types, weight:* Fresh material, silica dried, and herbarium sample, 10–25 mg | *Kits and protocols:* QIAGEN DNeasy Plant Mini Kit  *Tissue type, weight:* Herbarium sample, 20 mg | *Kits and protocols:*  CTAB and Sorbitol (Štorchová et al., 2000)  E.Z.N.A (R) Plant DNA kit  Invisorb Spin Plant Mini Kit  *Tissue types, weight:* Silica dried and herbarium sample, 10–20 mg |
| **2: Check gDNA fragment size and concentration** | *Size check*: 0.9–1.2% agarose gel with Thermofisher 1 Kb plus DNA ladder and 100-bp DNA ladder, or Fragment analyser (Thermo Fisher).  *Concentration measure*: Thermo Fisher Qubit, or Fragment analyser (Thermo Fisher).  *Target concentration*: 5–max ng/µL | *Size check*: either no size check or checking in 1% agarose gel with GenRuler DNA ladder  *Concentration measure*: Thermo Fisher Qubit  *Target concentration*: >500 ng of DNA in 60 µL, preferably 1 μg of DNA | *Size check*: 1% agarose gel with GenRuler DNA ladder  *Concentration measure*: Thermo Fisher Qubit  *Target concentration*: >500 ng of DNA in 60 µL, preferably 1 μg of DNA | *Size check*: 1% agarose gel with Thermo Fisher 1 Kb plus DNA ladder and 100-bp DNA ladder  *Concentration measure*:  Thermo Fisher Qubit  *Target concentration*: 10–13 ng/µL |
| **3: gDNA fragmentation**  **Target size: ~500 bp**  ***NB: For Covaris and QSonica, do a test run with different timings first and check fragment sizes on an agarose gel using samples with sufficient volume.*** | NB: Shearing is only conducted if gDNA fragments are >400 bp. No shearing if there is a “smear” and >50% gDNA fragments are <400 bp. See example images below.  *gDNA:* 1 µg in 70 µL  *Shearing:* Covaris S220. (Focused-ultrasonicator; Covaris Inc., Germany)  *Covaris settings*:  5% Duty  5 Intensity  200 Cycles/burst  55 s | Shearing always conducted no matter how fragmented the gDNA is.  Shearing when samples are not heavily fragmented (band pattern in the pre-shearing gel) or if fragmented <400 bp.  Target size: 500 bp  *gDNA:* 1µg in 70 µL  *Shearing:* QSonica 700 sonicator (Newtown, CT, USA)  *QSonica settings:*  Usually 7 min total, with 10 s of sonication followed by 10 s without sonication (pulsing), 20% amplitude. Some samples were sonicated by 5 or 9 min total, depending on gel results. | Shearing always conducted no matter how fragmented the gDNA is.  Shearing when samples are not heavily fragmented (band pattern in the pre-shearing gel) or if fragmented <400 bp.  Target size: 500 bp  *gDNA:* 1 µg in 70 µL  *Shearing:* QSonica 700 sonicator (Newtown, CT, USA)  *QSonica settings:* usually 3–5 min total, with 10 s of sonication followed by 10 s without sonication (pulsing), 20% amplitude. | Shearing always conducted no matter how fragmented the gDNA is.  Shearing is only conducted if gDNA fragments are >400bp. No shearing if there is a “smear” and >50% gDNA fragments are <400bp.  Target size: 500 bp  *gDNA:*0.6-0.8 μg in 62 µL  *Shearing:* Covaris M220 (Covaris Ltd., United Kingdom)  *Covaris settings*:  20% Duty  50 Peak Power  200 Cycles/burst  25 s |
| **3: Check sheared gDNA fragment size and concentration** | Same as step 2 for samples that were sheared. | 1% agarose gel | 1% agarose gel | Same as step 2 |
| **4: Library preparation** | *Kit and protocol*: NEBNext Ultra II.  *Primers:* NEBNext dual indexing (E7600, in this kit there are 8 i5 index primers and 12 i7 index primers for dual indexing)  *Size selection*: 300–400 bp. GC Biotech cleanNA (not in the recommended list of beads from NEB Next but works well)  *PCR protocol:* as per NEBNext step 4.1.3. with 15 cycles | *Kit and protocol*: NEBNext Ultra, NEBNext Ultra II and TruSeq  *Primers:* NEBNext E6609 (in this kit 96 8-base index primers are pre-mixed with the universal primer) or E7335, E7500  *Size selection*: 300–400 bp.  AMPure XP  beads as recommended in NEBNext Ultra II protocol  *PCR protocol* as per NEBNext step 4.1.3. with 15 cycles | *Kit and protocol*: NEBNext Ultra II  *Primers:* NEBNext E6609  *Size selection*: 300–400 bp. AMPure XP  beads as recommended in NEBNext Ultra II protocol  *PCR protocol:* as per NEBNext step 4.1.3. with 15 cycles | *Kit and protocol*: NEBNext Ultra II (E7370)  *Primers:* Single index NEBNext (E7335, 7500)  *Size selection*: 500 bp. 1% agarose gel-based selection with Thermo Fisher 1 Kb Plus DNA ladder and 100-bp DNA ladder Plus  *PCR protocol:* same as Berlin with 8 cycles |
| **5: Check size and concentrations** | *Size check*: Agilent BioAnalyser (chip of 11 samples at one time, expensive) or Agilent 4200 TapeStation (1–96 samples, cheaper and similar quality to BioAnalyser)  NB: If small fragments still present in any library, carry out a second clean up of small fragments (as per step 5 in NEBNext Ultra II protocol).  *Concentration measure*: as in step 2 | *Size check*: Combination of Agilent BioAnalyser (chip of 11 samples at one time) for some samples and 1% agarose gel for remaining to lower costs  *Concentration measure*: as in step 2 | *Size check*: 1% agarose gel  *Concentration measure*: as in step 2 | *Size check*: None, because of gel-based fragment size selection (step 4)  *Concentration measure*: As in step 2 (Qubit) |
| **6: Pool libraries (equimolar) ready for hybrid capture (require 7 μL of pool)** | *Pool*: 4–12 libraries  *Target concentration for pool:* 100–500 ng per library in 7 µL  NB: check library pool concentration as in step 5. | *Pool*: 4 libraries  *Target concentration for pool:* 125 ng per library in 7 µL | *Pool*: 4 libraries  *Target concentration for pool:* 125 ng per library in 7 µL | *Pool*: 18–24 libraries  *Target concentration for pool:* 100–500 ng per library in 7 µL |
| **7: Hybrid capture and amplification of enriched library** | *Kit and protocol*: MyBaits COS Compositae/Asteraceae1kv1 (versions 2–3).  *Hybridization temperature*: 65°C  *Hybridization time*: 24–27 h  *Polymerase for amplification of hybrid capture reactions:* KAPA HiFi (Kapa Biosystems)  *PCR protocol for amplification of hybrid capture reactions*: Activation at 98°C for 2 min, 14–16 cycles of: [denaturation at 98°C for 20 s, annealing at 55°C for 30 s, extension at 72̊C for 30 s], and a final extension of 72°C for 5 min  *Clean up of amplified captures:* as per step 5 in NEBNext Ultra II protocol | *Kit and protocol*: MyBaits COS Compositae/Asteraceae 1kv1 (versions 1–3)  *Hybridization temperature*: 65°C  *Hybridization time*: 36 h  *Polymerase for amplification of hybrid capture reactions:* KAPA HiFi (Kapa Biosystems)  *PCR protocol for*  *amplification of hybrid capture reactions*: as for Berlin except for: no. of cycles: 16 and annealing temperature: 60°C  *Clean up of amplified captures:* as per step 5 in NEBNext Ultra II protocol | *Kit and protocol*: MyBaits COS Compositae/Asteraceae 1kv1 (versions 2–3)  *Hybridization temperature*: 65°C  *Hybridization time*: 40–46 h  *Polymerase for amplification of hybrid capture reactions:* KAPA HiFi (Kapa Biosystems)  *PCR protocol for*  *amplification of hybrid capture reaction*: as for Memphis  *Clean up of amplified captures:* as per step 5 in NEBNext Ultra II protocol | *Kit and protocol*: MyBaits COS Compositae/Asteraceae 1kv1 (versions 2–3)  *Hybridization temperature*: 65°C  *Hybridization time*: 26 h  *Polymerase for amplification of hybrid capture reactions:* KAPA HiFi (Kapa Biosystems)  *PCR protocol for*  *amplification of hybrid capture reaction:* Activation at 98°C for 2 min, 12 cycles of: [denaturation at 98°C for 20 s, annealing at 62°C for 30 s, extension at 72°C for 45 s], and a final extension of 72°C for 5 min  *Clean up of amplified captures:* QIAquick PCR Purification Kit and AMPure XP |
| **8: Equimolar pooling of libraries, quality check and high-throughput sequencing,**  **with option to spike post-capture libraries with pre-capture libraries** | *Quality check:* Size and concentration assessment as per step 5 (see example of FragmentAnalyser graph of final sample for sequencing)  *Platform(s), # libraries on one lane:*  NextSeq (v2 mid output 300 cycles), 1–20  NB: other high-throughput sequencing approaches used but not in this paper:  MiSeq v. 2 300 cycles, 18–24  HiSeqX 300 cycles (Macrogen), 80–96. | *Quality check:* Size and concentration assessment as per step 5  *Platform(s), # libraries on one lane:*  Illumina MiSeq (v2, 300 cycles), 24  Illumina HiSeq 2000 (200 cycles), 18 (*Helianthus*)  Illumina HiSeq 2500 High (300 cycles), 48 (*Chresta*)  Illumina MiSeq (v2, 300 cycles), 28 (*Antennaria*) | *Pooling pre- and post-capture libraries:* 33.3% unenriched with 66.7% enriched library  *Quality check:* size and concentration assessment as per step 5  *Platform(s), # libraries on one lane:*  Illumina HiSeq 3000 (200 cycles), 96 (*Cousinia*) | *Pooling pre- and post-capture libraries:* 50% unenriched with 50% enriched library (*Picris*)  *Quality check:* Qubit and Bioanalyzer  *Platform(s), # libraries on one lane:*  Illumina MiSeq (v2, 300 cycles), 18–24 |

**Example of pooling: To create pool #1 for hybrid capture containing library numbers 1**–**9**

| Pool number | Library number | **Library conc measurement (ng/µL)** | Volume in pool (µL) | ng of library in pool | Total volume of pool | Total ng of pool | ng in 7 µL for hybrid capture |
| --- | --- | --- | --- | --- | --- | --- | --- |
| Pool 1 | 1 | 22.5989 | 7.00 | 158.19 | 32 | 1423.73 | 309.29 |
|  | 2 | 40.33 | 3.92 | 158.19 |  |  |  |
|  | 3 | 54.1037 | 2.92 | 158.19 |  |  |  |
|  | 4 | 58.2536 | 2.72 | 158.19 |  |  |  |
|  | 5 | 85.4486 | 1.85 | 158.19 |  |  |  |
|  | 6 | 39.7557 | 3.98 | 158.19 |  |  |  |
|  | 7 | 46.9524 | 3.37 | 158.19 |  |  |  |
|  | 8 | 53.3424 | 2.97 | 158.19 |  |  |  |
|  | 9 | 45.2595 | 3.50 | 158.19 |  |  |  |

To calculate volumes needed of each library in pool #1:

1. Select the library with lowest concentration (library 1 highlighted in red above) and use 7 μL of that library.
2. Calculate ng of library 1 in pool (7 μl × library concentration in ng/µL = 158.19 here).
3. Calculate volume needed for all other libraries in order to have the same ng as pool 1 (library concentration/158.19)
4. Calculate final ng of the pool in 7 μL for hybrid capture (total ng in the pool × 7/total volume of the pool).

**Final pool for sequencing example of peak distribution**

**(TapeStation peak graph: High Sensitivity D1000 ScreenTape)**


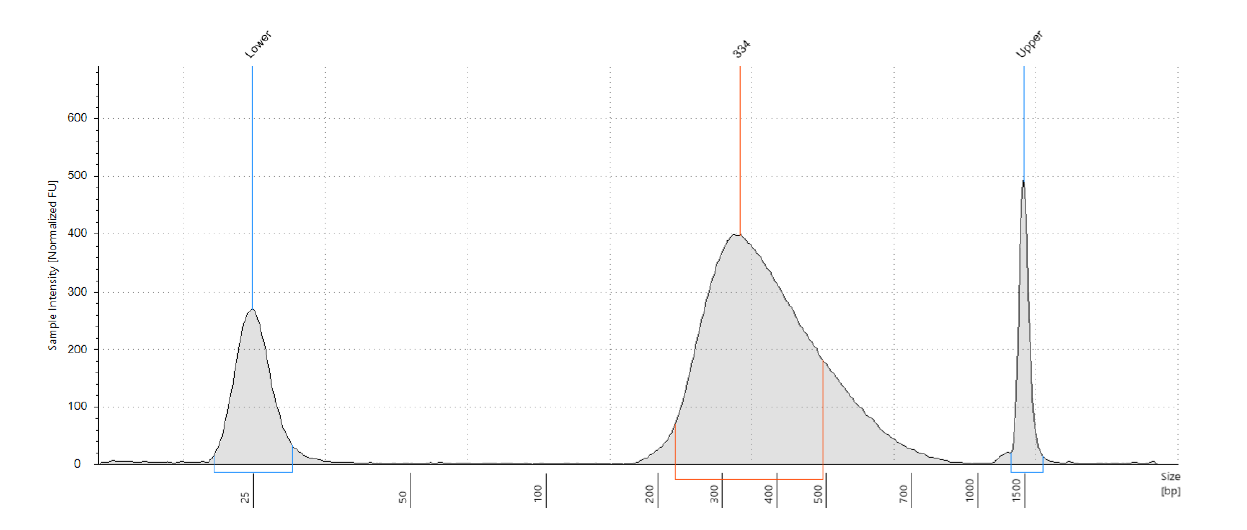


**LITERATURE CITED**

Štorchová, H., R. Hrdličková, J. Chrtek, M. Tetera, D. Fitze, and J. Fehrer. 2000. An improved method of DNA isolation from plants collected in the field and conserved in saturated NaCl/CTAB solution *Taxon* 49 (1): 79-84. doi: 10.2307/1223934
