# Supplementary material for: An empirical assessment of a single family‐wide hybrid capture locus set at multiple evolutionary timescales in Asteraceae
Source: Appl Plant Sci. 2019 Oct 25;7(10):e11295. doi: 10.1002/aps3.11295 (PMC6814182; doi:10.1002/aps3.11295)
Supplement: Supplementary file 4 — APPENDIX S4. Preliminary assessment of two trimming approaches of supercontig (exon + flanking intron region) alignments (the splash‐zone) using trimAl. [file APS3-7-e11295-s004.docx]

**Appendix S4.** Preliminary assessment of two trimming approaches of supercontig (exon + flanking intron region) alignments (the splash-zone) using trimAl.

Trimal v. 1.4 (Capella-Gutiérrez et al., 2009) was used to remove spurious sequences from each alignment in all data sets. We first tested two trimming approaches for two data sets (Cichorieae-wide and *Picris* species complex) and manually checked ~30 alignments of each data set in order to select an approach for all other data sets. The two trimming approaches were: (1) “strict” (-resoverlap and -seqoverlap were set to 0.75 and 0.80, respectively) and (2) “less-strict” (-resoverlap and -seqoverlap were set to 0.65 and 70, respectively). In addition, we used the -gappyout parameter, which efficiently removes poorly aligned regions (Capella-Gutiérrez et al., 2009). We manually checked ~30 alignments from each data set and found that the “less-strict” approach described above generated reliable alignments and minimized numbers of samples that were removed. Therefore, all other sample groups were subject to the following parameters in trimal: -resoverlap and -seqoverlap = 0.65 and 70.

The directories in Dryad DOI: <https://datadryad.org/review?doi=doi:10.5061/dryad.60vb576> contain alignments for the Cichorieae-wide and the *Picris hieracioides* species complex supercontig alignments (non-paralagous loci).

| **Directory name** | **Contents** |
| --- | --- |
| Cichorieae_supercontig_preliminary | Directories containing all alignments of supercontigs for the Cichorieae wide data set trimmed using the “strict” and “less strict” settings described above. Summary statistics of alignments are also provided. |
| Picris_complex_preliminary | Directories containing alignments of supercontigs for the *Picris hieracioides* species complex data set trimmed using the “strict” and “less strict” settings described above. Summary statistics of alignments are also provided. |

**LITERATURE CITED**

Capella-Gutiérrez, S., J. M. Silla-Martínez, and T. Gabaldón. 2009. trimAl: A tool for automated alignment trimming in large-scale phylogenetic analyses. *Bioinformatics* 25(15): 1972–1973. doi: https://doi.org/10.1093/bioinformatics/btp348
