# Supplementary material for: An empirical assessment of a single family‐wide hybrid capture locus set at multiple evolutionary timescales in Asteraceae
Source: Appl Plant Sci. 2019 Oct 25;7(10):e11295. doi: 10.1002/aps3.11295 (PMC6814182; doi:10.1002/aps3.11295)
Supplement: Supplementary file 5 — APPENDIX S5. Phylogenetic analyses of the Cichorieae tribe‐exon‐complete data set (218 loci) revealed inconsistencies in topological inferences for Picris amalecitana between maximum likelihood and coalescent species tree (ASTRAL) analyses. [file APS3-7-e11295-s005.pdf]

DOI

APPENDIX S5. Phylogenetic analyses of the Cichorieae tribe-exon-complete data set (218 loci) revealed inconsistencies in topological inferences for *Picris amalecitana* between maximum likelihood and coalescent species tree (ASTRAL) analyses. RAxML-NG trees are presented (A) without partitioning and (B) with partitioning and substitution models. Green lines in the RAxML-NG tree with partitioning (B) indicate well-resolved nodes that are either unresolved or with lower bootstrap support in the RAxML-NG tree without partitioning (A) and ASTRAL species tree (C). Subtribe names are indicated next to their corresponding nodes. Blue backbone nodes indicate intersubtribal nodes that are newly resolved compared to previous studies on Cichorieae.

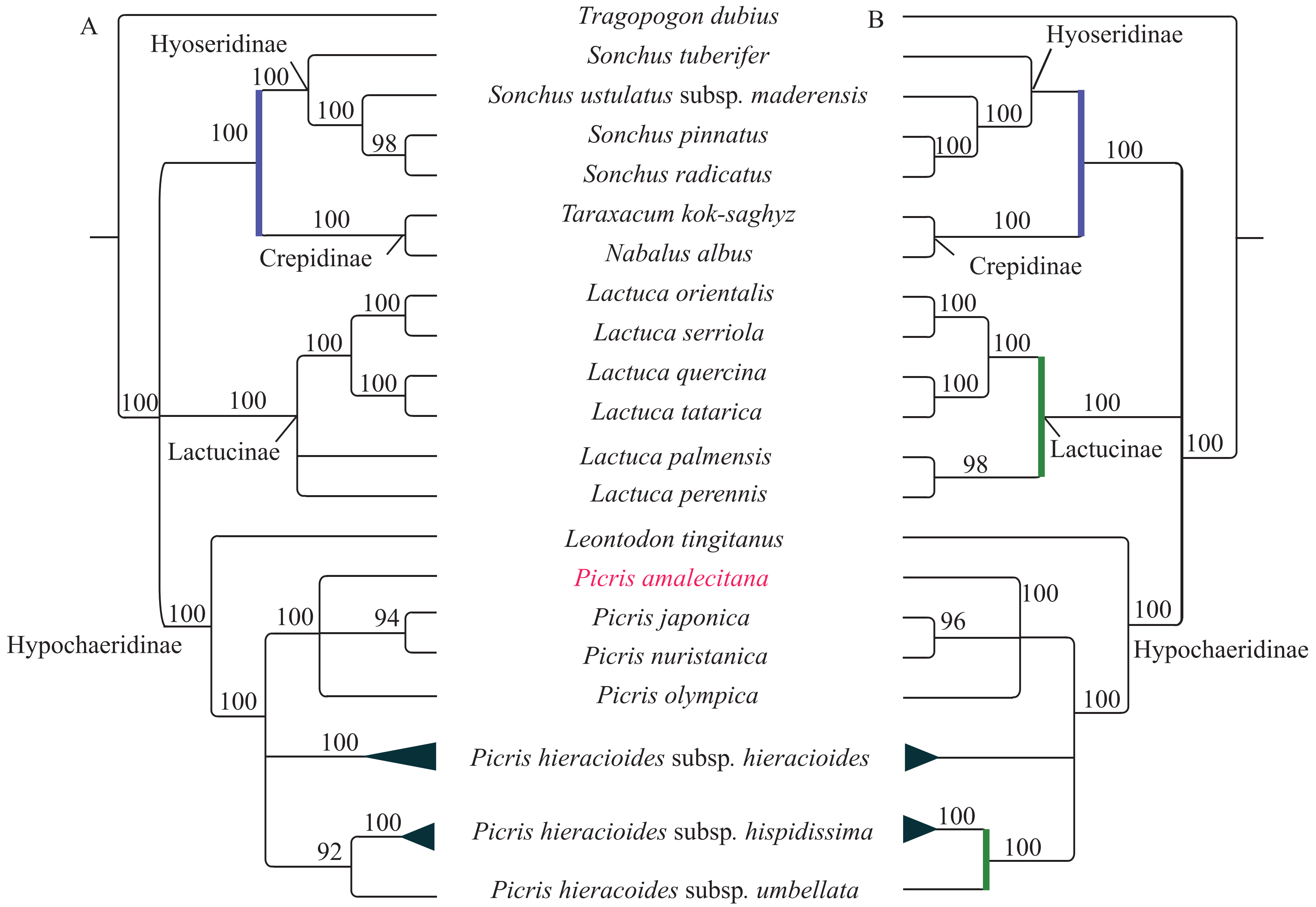

DOI

C

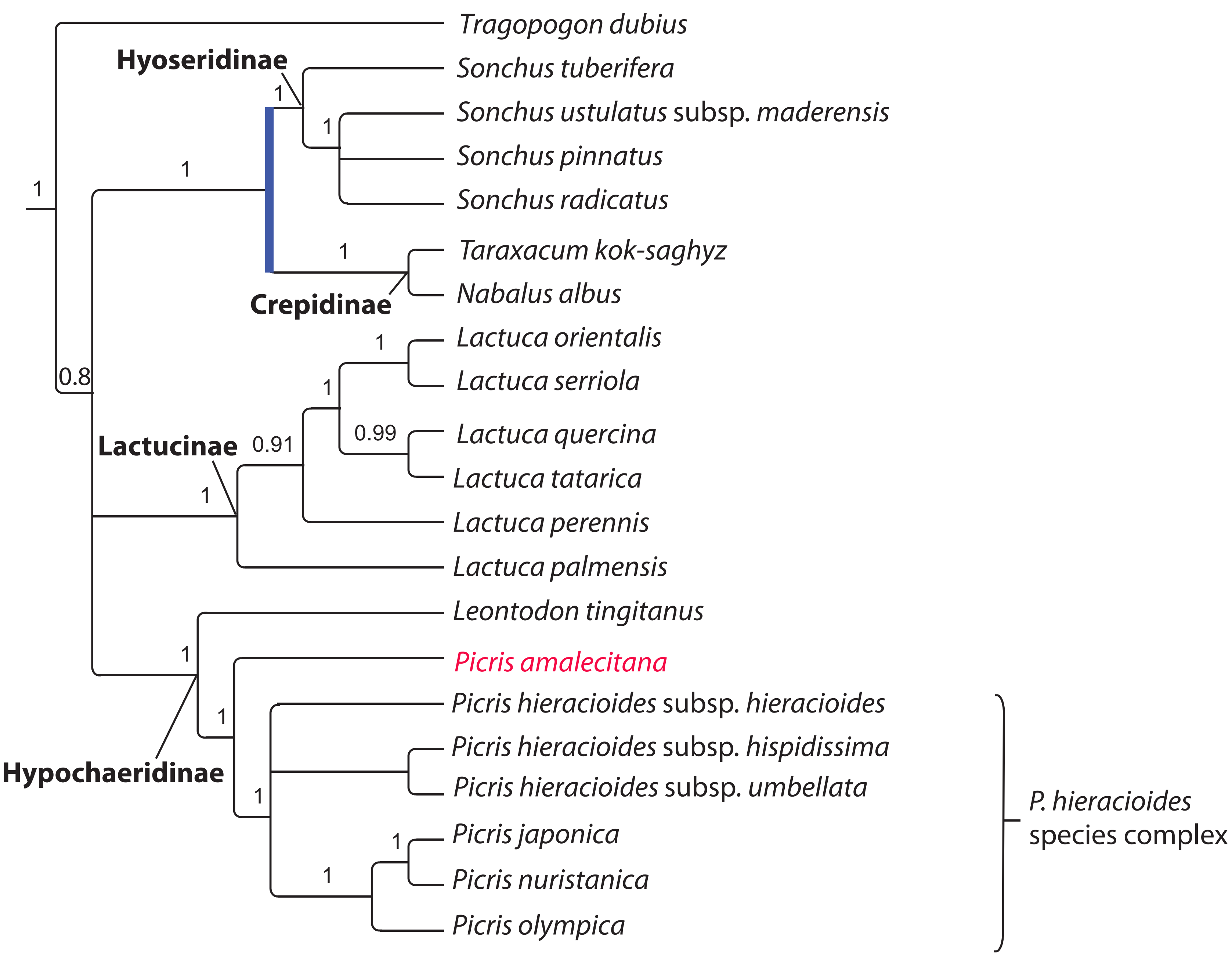
Tragopogon dubius
Hyoseridinae
Sonchus tuberifera
Sonchus ustulatus subsp. maderensis
Sonchus pinnatus
Sonchus radicatus
Taraxacum kok-saghyz
Nabalus albus
Crepidinae
Lactuca orientalis
Lactuca serriola
Lactucinae
Lactuca quercina
Lactuca tatarica
Lactuca perennis
Lactuca palmensis
Leontodon tingitanus
Picris amalecitana
Picris hieracioides subsp. hieracioides
Picris hieracioides subsp. hispidissima
Picris hieracioides subsp. umbellata
Hypochaeridinae
Picris japonica
Picris nuristanica
Picris olympica
P. hieracioides species complex
1
0.8
0.91
0.99
