# Supplementary material for: An empirical assessment of a single family‐wide hybrid capture locus set at multiple evolutionary timescales in Asteraceae
Source: Appl Plant Sci. 2019 Oct 25;7(10):e11295. doi: 10.1002/aps3.11295 (PMC6814182; doi:10.1002/aps3.11295)

DOI

APPENDIX S8. Area-proportional Venn diagrams for each tribe illustrating the proportions of non-paralogous loci that are unique to each genus, species complex, or species sampled. *Species-level sampling within *Carlina vulgaris*, ***Picris hieracioides* species complex-level sampling. For total numbers of non-paralogous loci for each genus, refer to Table 1. Refer to Appendix 1 for sample details of each genus.

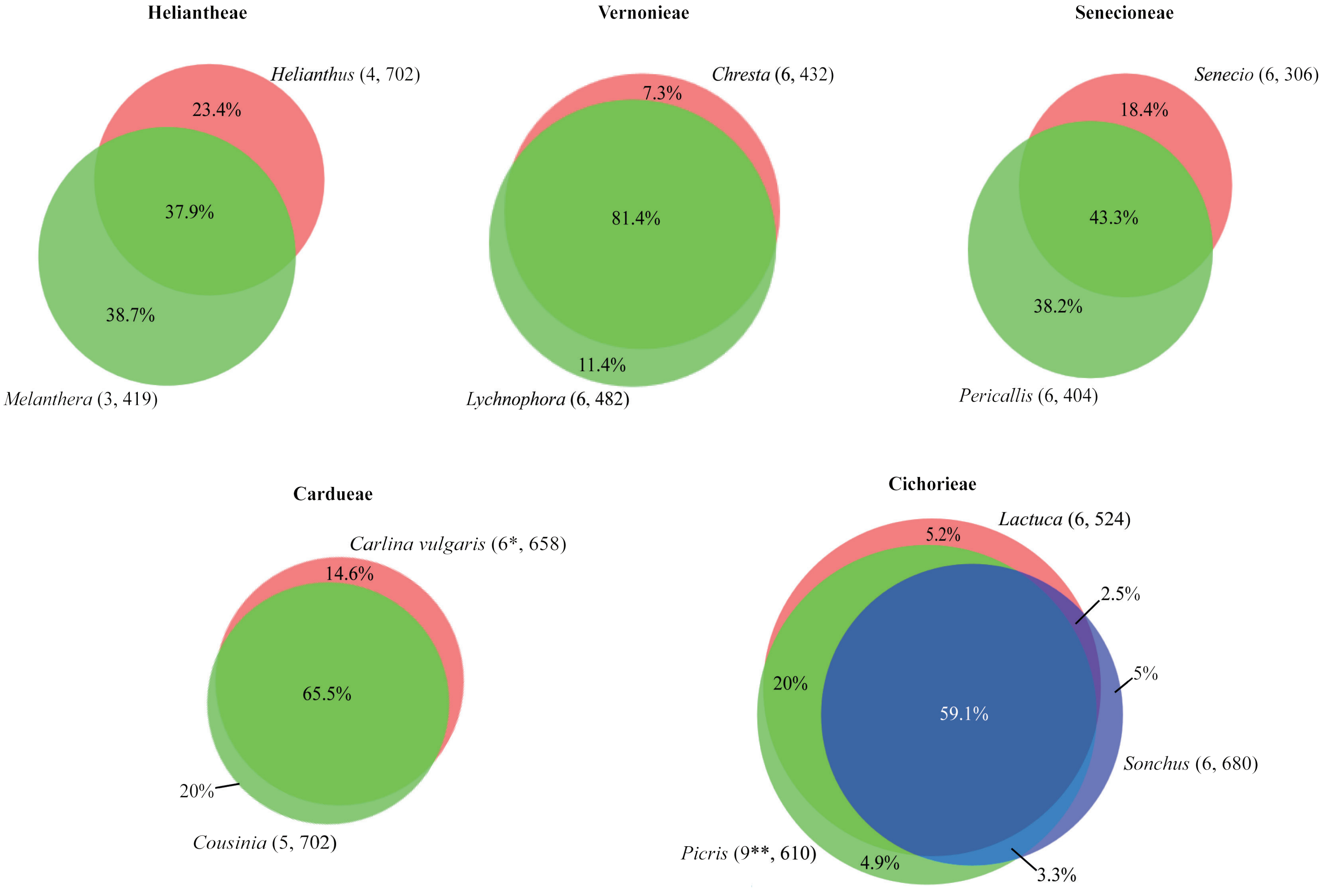

Supplement: Supplementary file 8 — APPENDIX S8. Area‐proportional Venn diagrams for each tribe illustrating the proportions of non‐paralogous loci that are unique to each genus, species complex, or species sampled. [file APS3-7-e11295-s008.pdf]
