# Supplementary material for: An empirical assessment of a single family‐wide hybrid capture locus set at multiple evolutionary timescales in Asteraceae
Source: Appl Plant Sci. 2019 Oct 25;7(10):e11295. doi: 10.1002/aps3.11295 (PMC6814182; doi:10.1002/aps3.11295)
Supplement: Supplementary file 13 — APPENDIX S13. ASTRAL species tree and RAxML‐NG trees with and without partitioning based on the Tribe‐exon‐shrunken data set and ASTRAL species tree of the Tribe‐supercontig‐complete data set. Scale bars indicate the expected number of nucleotide substitutions per site. [file APS3-7-e11295-s013.pdf]

Appendix S13. ASTRAL species tree and RAxML-NG tree with partitioning based on the Tribe-exon-shrunken data set and ASTRAL species tree of the tribe-supercontig-complete data set. (A) ASTRAL tree of the tribe-exon-shrunken data set. (B) RAxML analyses (with partitioning and substitution models) of the tribe-exon-shrunken data set. (C) ASTRAL tree of the tribe-supercontig data set. Scale bars indicate the expected number of nucleotide substitutions per site. In the ASTRAL trees, the numbers on nodes indicate posterior probability values and on the ML trees the numbers indicate percentage bootstrap support.

A

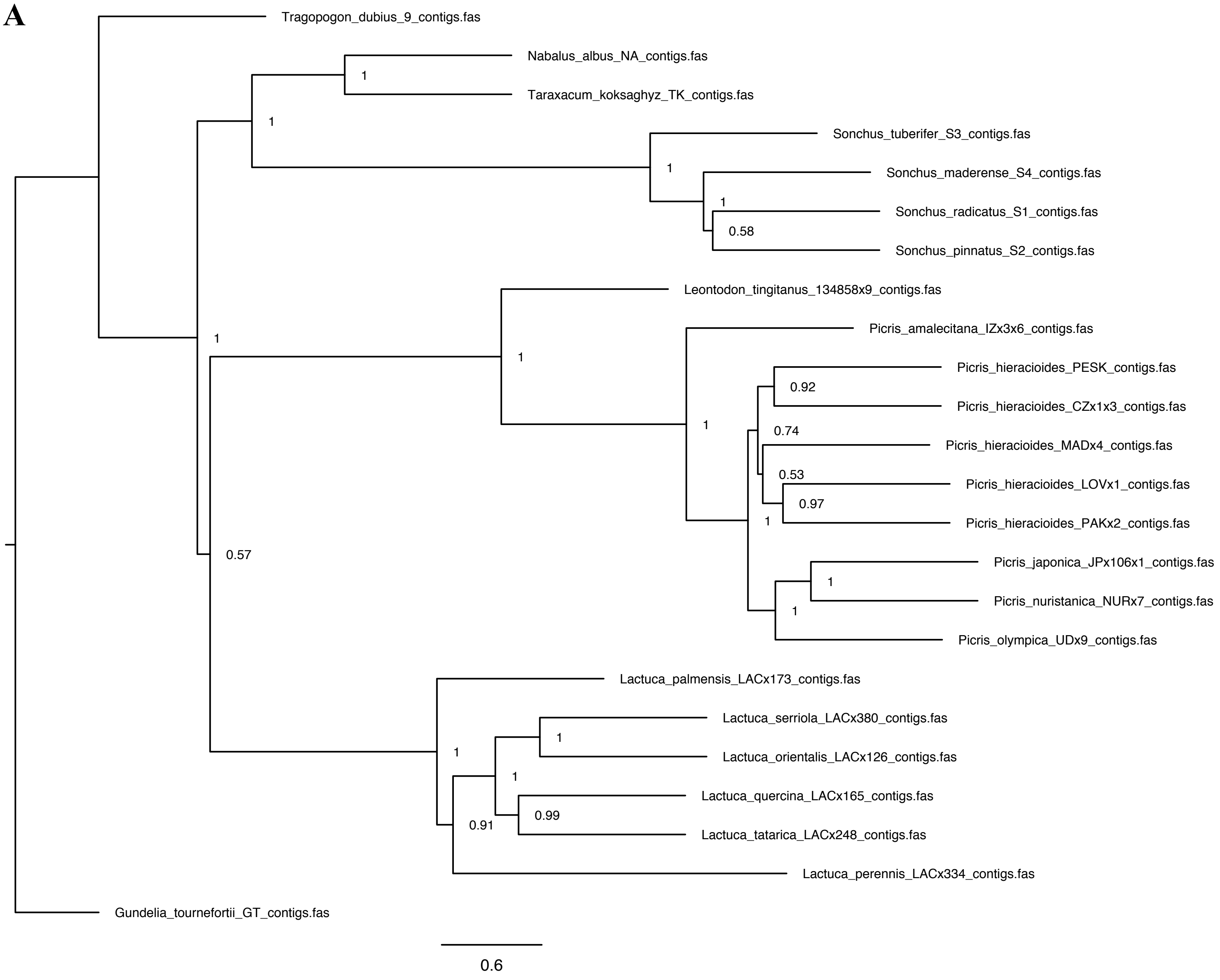

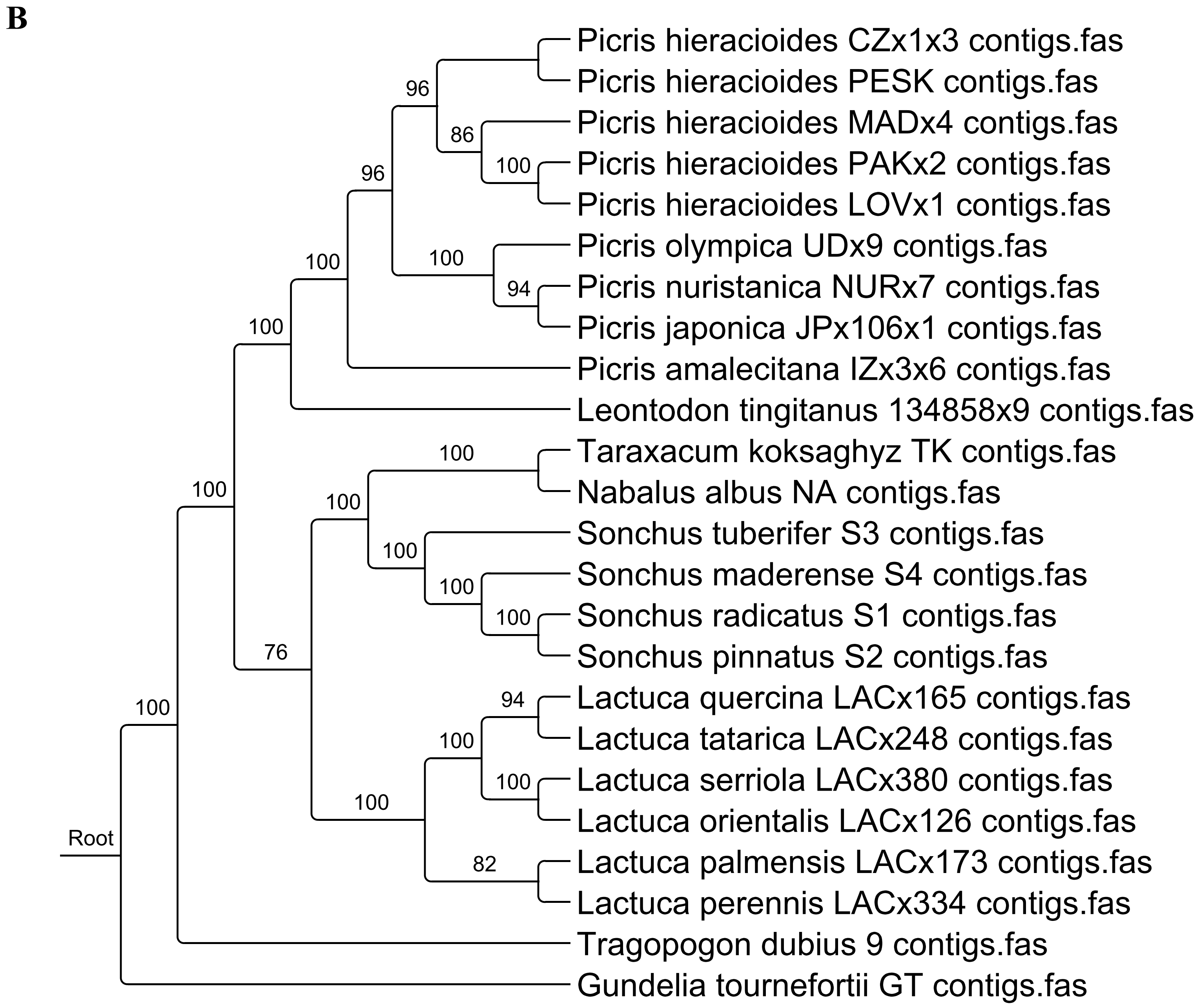
B
Picris hieracioides CZx1x3 contigs.fas
Picris hieracioides PESK contigs.fas
Picris hieracioides MADx4 contigs.fas
Picris hieracioides PAKx2 contigs.fas
Picris hieracioides LOVx1 contigs.fas
Picris olympica UDx9 contigs.fas
Picris nuristanica NURx7 contigs.fas
Picris japonica JPx106x1 contigs.fas
Picris amalecitana IZx3x6 contigs.fas
Leontodon tingitanus 134858x9 contigs.fas
Taraxacum koksaghyz TK contigs.fas
Nabalus albus NA contigs.fas
Sonchus tuberifer S3 contigs.fas
Sonchus maderense S4 contigs.fas
Sonchus radicatus S1 contigs.fas
Sonchus pinnatus S2 contigs.fas
Lactuca quercina LACx165 contigs.fas
Lactuca tatarica LACx248 contigs.fas
Lactuca serriola LACx380 contigs.fas
Lactuca orientalis LACx126 contigs.fas
Lactuca palmensis LACx173 contigs.fas
Lactuca perennis LACx334 contigs.fas
Tragopogon dubius 9 contigs.fas
Gundelia tournefortii GT contigs.fas
Root
96
96
86
100
100
100
94
100
100
100
100
100
100
100
76
100
94
100
100
100
82

C

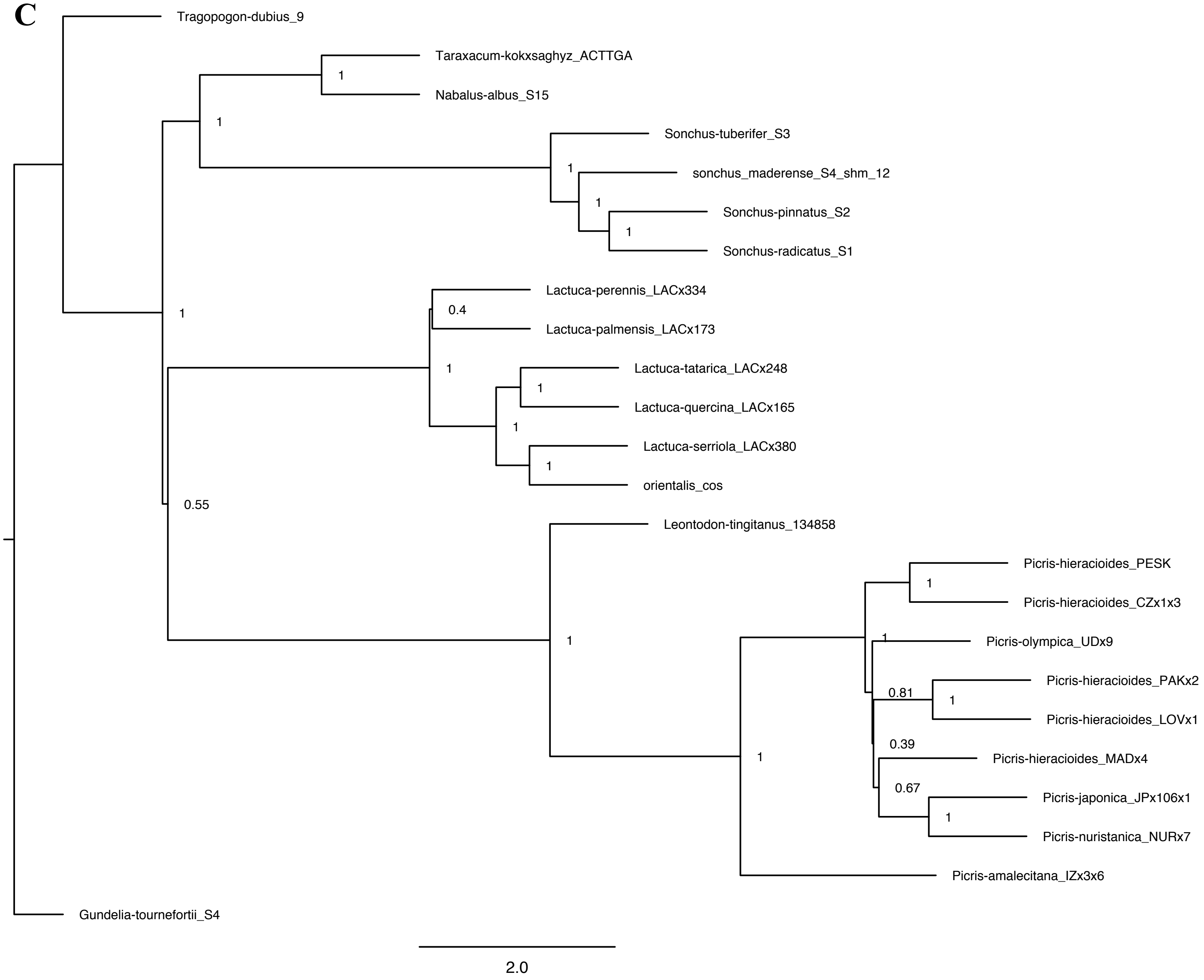
Tragopogon-dubius_9
Taraxacum-kokxsaghyz_ACTTGA
Nabalus-albus_S15
Sonchus-tuberifer_S3
sonchus_maderense_S4_shm_12
Sonchus-pinnatus_S2
Sonchus-radicatus_S1
Lactuca-perennis_LACx334
Lactuca-palmensis_LACx173
Lactuca-tatarica_LACx248
Lactuca-quercina_LACx165
Lactuca-serriola_LACx380
orientalis_cos
Leontodon-tingitanus_134858
Picris-hieracioides_PESK
Picris-hieracioides_CZx1x3
Picris-olympica_UDx9
Picris-hieracioides_PAKx2
Picris-hieracioides_LOVx1
Picris-hieracioides_MADx4
Picris-japonica_JPx106x1
Picris-nuristanica_NURx7
Picris-amalecitana_IZx3x6
Gundelia-tournefortii_S4
1
1
1
1
1
1
0.4
1
1
1
1
0.55
1
1
1
1
0.81
1
0.39
0.67
1
2.0
