# Supplementary material for: An empirical assessment of a single family‐wide hybrid capture locus set at multiple evolutionary timescales in Asteraceae
Source: Appl Plant Sci. 2019 Oct 25;7(10):e11295. doi: 10.1002/aps3.11295 (PMC6814182; doi:10.1002/aps3.11295)
Supplement: Supplementary file 14 — APPENDIX S14. Picris hieracioides species complex similarity matrices based on the Picris‐610‐exon‐complete and ‐shrunken and Picris‐supercontig data sets, and networks based on the Picris‐610‐exon‐complete and ‐shrunken data sets (see data set information in Table 2). [file APS3-7-e11295-s014.pdf]

DOI

Appendix S14. *Picris hieracioides* species complex similarity matrices based on the Picris-610-exon-complete (A) and -shrunken (B) and Picris-supercontig (C) data sets, and networks based on the Picris-610-exon-complete (D) and -shrunken (E) data sets (see data set information in Table 2). (A) *Picris* species complex similarity matrix based on 218 exons from the Cichorieae tribe-wide data sets after trimming of spurious sequences and removal of loci that are potentially paralogous across the tribe-wide Cichorieae data set. (B) *Picris* species complex similarity matrix based on 610 exons after trimming of spurious sequences and removal of loci that are potentially paralogous across the *Picris* data set. (C) *Picris* species complex supercontigs similarity matrix based on 576 supercontigs (exons + flanking intron regions) after trimming of spurious sequences and removal of loci that are potentially paralogous across the *Picris* data set). Percentage similarities are given in the boxes; shading corresponds to: darker = more similar, lighter = less similar. Distances between samples are higher in the supercontig data set (C) compared to the two exon data sets (A and B).

A

Picris-amalecitana_IZx3x6		95.4%	95.3%	95.8%	95.2%	95.5%	95.6%	95.6%	95.8%
Picris-nuristanica_NURx7	95.4%		97.2%	97.3%	97.2%	97.3%	97.6%	97.5%	97.6%
Picris-japonica_JPx106x1	95.3%	97.2%		96.9%	96.9%	97.2%	97.4%	97.4%	97.4%
Picris-olympica_UDx9	95.8%	97.3%	96.9%		97.5%	97.6%	97.6%	97.7%	97.6%
Picris-hieracioides_PESK	95.2%	97.2%	96.9%	97.5%		97.4%	97.4%	97.4%	97.8%
Picris-hieracioides_CZx1x3	95.5%	97.3%	97.2%	97.6%	97.4%		97.8%	98.2%	97.8%
Picris-hieracioides_PAKx2	95.6%	97.6%	97.4%	97.6%	97.4%	97.8%		97.9%	97.9%
Picris-hieracioides_LOVx1	95.6%	97.5%	97.4%	97.7%	97.4%	98.2%	97.9%		97.9%
Picris-hieracioides_MADx4	95.8%	97.6%	97.4%	97.6%	97.8%	97.8%	97.9%	97.9%	

B

Picris-amalecitana_IZx3x6		96.7%	96.8%	97.0%	97.0%	96.9%	97.1%	96.9%	97.2%
Picris-nuristanica_NURx7	96.7%		98.0%	97.7%	97.7%	97.7%	98.0%	97.8%	97.8%
Picris-japonica_JPx106x1	96.8%	98.0%		97.8%	97.8%	97.8%	98.1%	97.9%	97.9%
Picris-olympica_UDx9	97.0%	97.7%	97.8%		98.1%	98.0%	98.1%	97.9%	98.1%
Picris-hieracioides_PESK	97.0%	97.7%	97.8%	98.1%		98.0%	98.1%	97.9%	98.3%
Picris-hieracioides_CZx1x3	96.9%	97.7%	97.8%	98.0%	98.0%		98.2%	98.1%	98.1%
Picris-hieracioides_PAKx2	97.1%	98.0%	98.1%	98.1%	98.1%	98.2%		98.1%	98.3%
Picris-hieracioides_LOVx1	96.9%	97.8%	97.9%	97.9%	97.9%	98.1%	98.1%		98.1%
Picris-hieracioides_MADx4	97.2%	97.8%	97.9%	98.1%	98.3%	98.1%	98.3%	98.1%	

C

Picris-amalecitana_IZx3x6		89.5%	91.1%	90.2%	90.4%	89.6%	87.8%	88.6%	91.4%
Picris-nuristanica_NURx7	89.5%		94.4%	92.7%	93.0%	92.3%	91.2%	91.8%	93.9%
Picris-japonica_JPx106x1	91.1%	94.4%		94.8%	95.4%	94.0%	91.6%	93.2%	96.6%
Picris-olympica_UDx9	90.2%	92.7%	94.8%		94.5%	93.3%	91.0%	92.7%	95.2%
Picris-hieracioides_PESK	90.4%	93.0%	95.4%	94.5%		94.4%	91.3%	92.4%	95.8%
Picris-hieracioides_CZx1x3	89.6%	92.3%	94.0%	93.3%	94.4%		91.3%	92.4%	94.2%
Picris-hieracioides_PAKx2	87.8%	91.2%	91.6%	91.0%	91.3%	91.3%		92.0%	91.4%
Picris-hieracioides_LOVx1	88.6%	91.8%	93.2%	92.7%	92.4%	92.4%	92.0%		93.2%
Picris-hieracioides_MADx4	91.4%	93.9%	96.6%	95.2%	95.8%	94.2%	91.4%	93.2%	

DOI

D

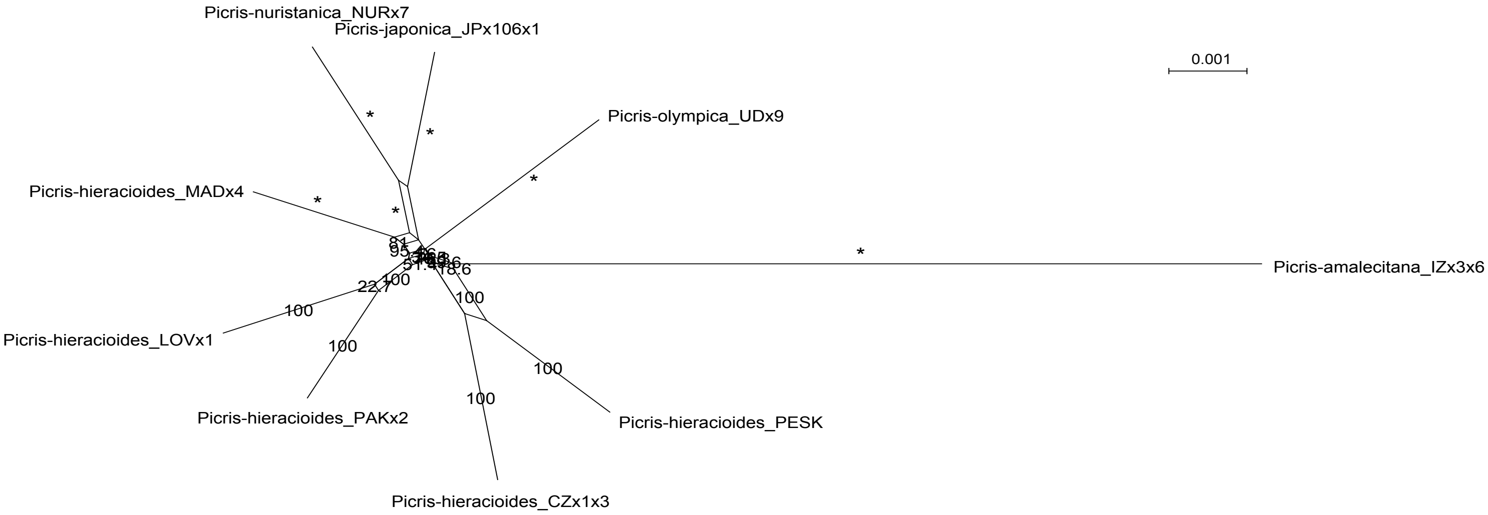
Picris-nuristanica_NURx7
Picris-japonica_JPx106x1
0.001
Picris-olympica_UDx9
Picris-hieracioides_MADx4
Picris-amalecitana_IZx3x6
Picris-hieracioides_LOVx1
Picris-hieracioides_PAKx2
Picris-hieracioides_PESK
Picris-hieracioides_CZx1x3
*
100
22.7
18.6

E

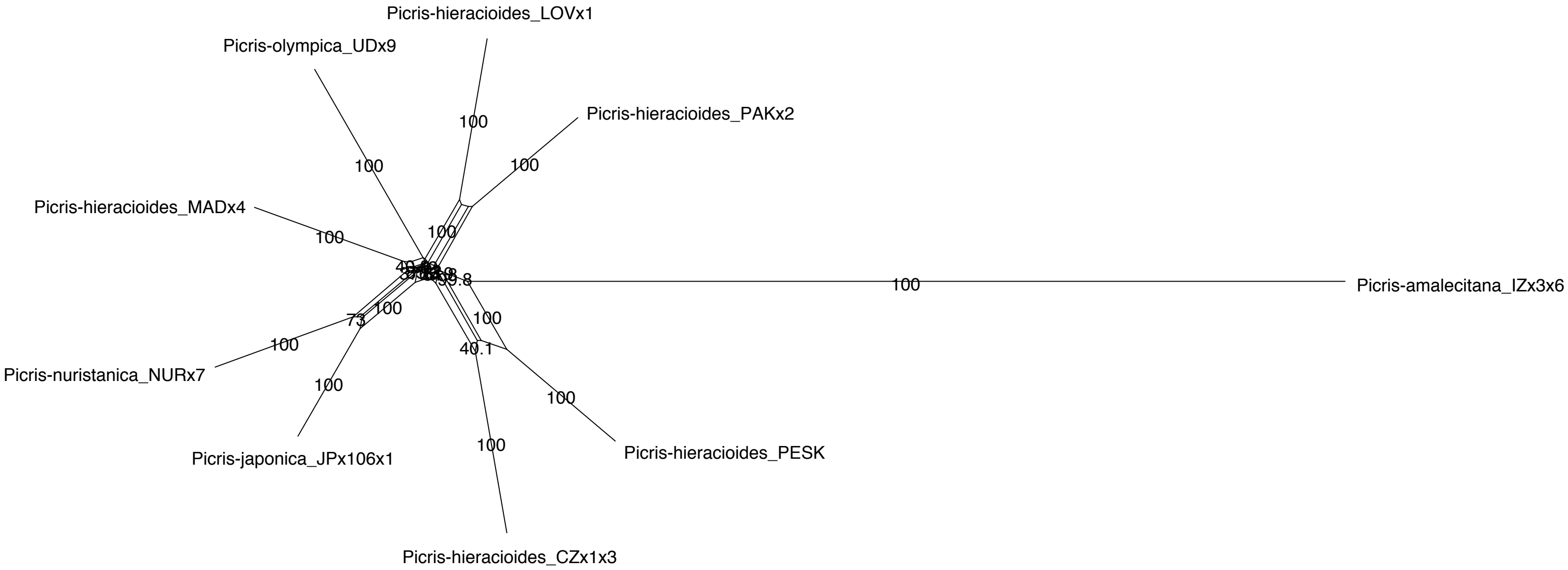
Picris-hieracioides_LOVx1
Picris-olympica_UDx9
Picris-hieracioides_PAKx2
Picris-hieracioides_MADx4
Picris-amalecitana_IZx3x6
Picris-nuristanica_NURx7
Picris-japonica_JPx106x1
Picris-hieracioides_PESK
Picris-hieracioides_CZx1x3
100
73
40.1
