# Supplementary material for: An empirical assessment of a single family‐wide hybrid capture locus set at multiple evolutionary timescales in Asteraceae
Source: Appl Plant Sci. 2019 Oct 25;7(10):e11295. doi: 10.1002/aps3.11295 (PMC6814182; doi:10.1002/aps3.11295)
Supplement: Supplementary file 15 — APPENDIX S15. Summaries of conflicting and concordant gene trees from phyparts with the tribe‐exon‐complete ASTRAL species trees and supercontig maximum likelihood (ML) and ASTRAL trees. [file APS3-7-e11295-s015.pdf]

Appendix S15. Summaries of conflicting and concordant gene trees from phyparts with the tribe-exon-complete ASTRAL species trees and supercontig maximum likelihood (ML) and ASTRAL trees. (A) phyparts analyses of the RAxML-NG species tree from the tribe-exon-complete data set. (B) phyparts analyses of the ASTRAL species tree from the tribe-exon-complete data set. (C) phyparts analyses of the RAxML-NG species tree from the tribe-exon-shrunken data set (201 gene trees with outgroup). (D) phyparts analyses of the RAxML-NG species tree from the tribe-exon-shrunken data set (139 gene trees with outgroup). For each branch, the top number indicates the number of gene trees concordant with the ML tree at that node and the bottom number indicates the number of gene trees in conflict with that node. The pie charts present the proportion of gene trees that support that clade (blue), the proportion that support the main alternative topology for that clade (yellow), the proportion that support the remaining alternatives topologies (red), and the proportion that inform (conflict or support) that clade that have <50% bootstrap support (gray). The scale bars in each tree correspond to expected number of nucleotide substitutions per site.

A

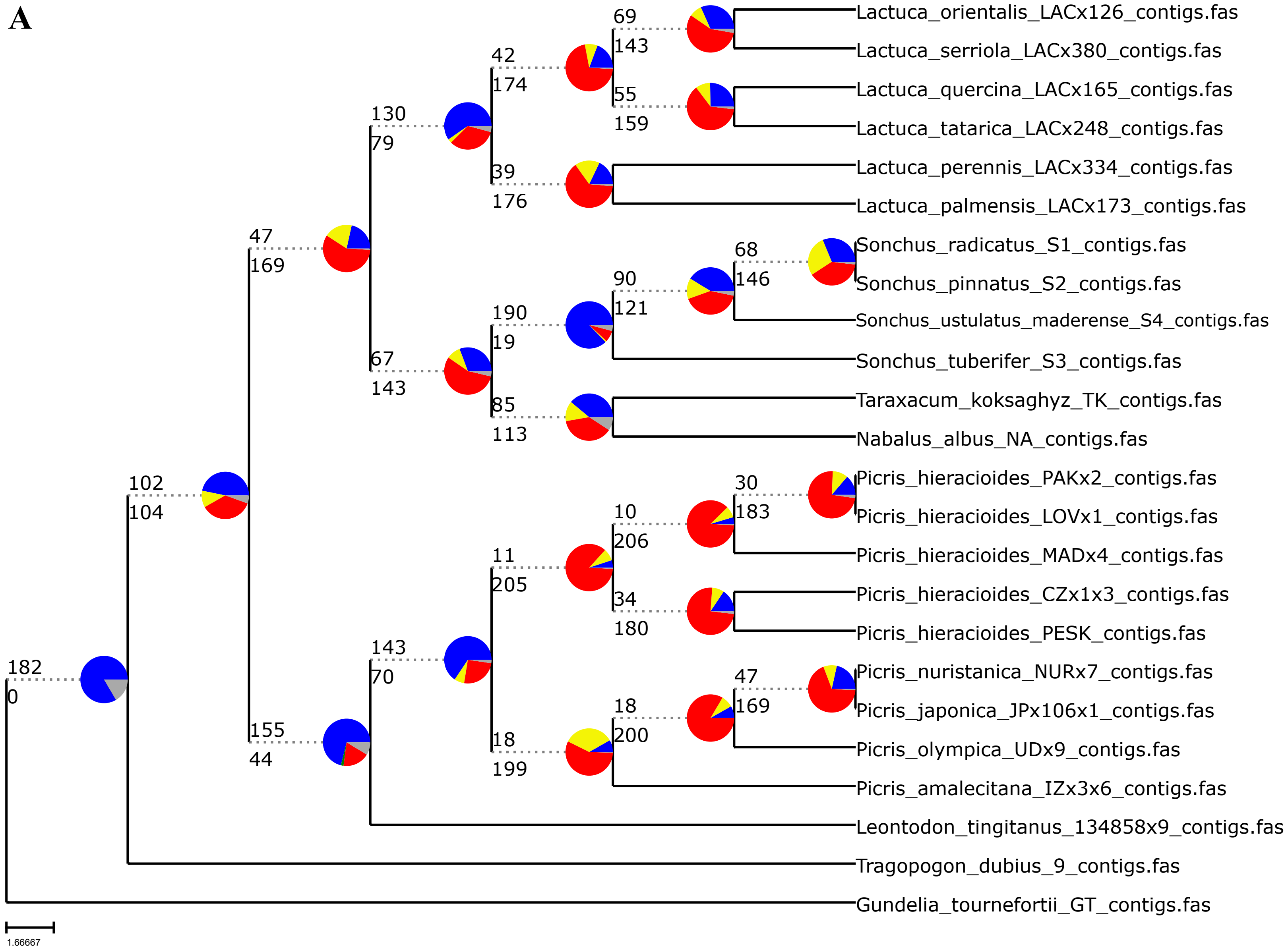

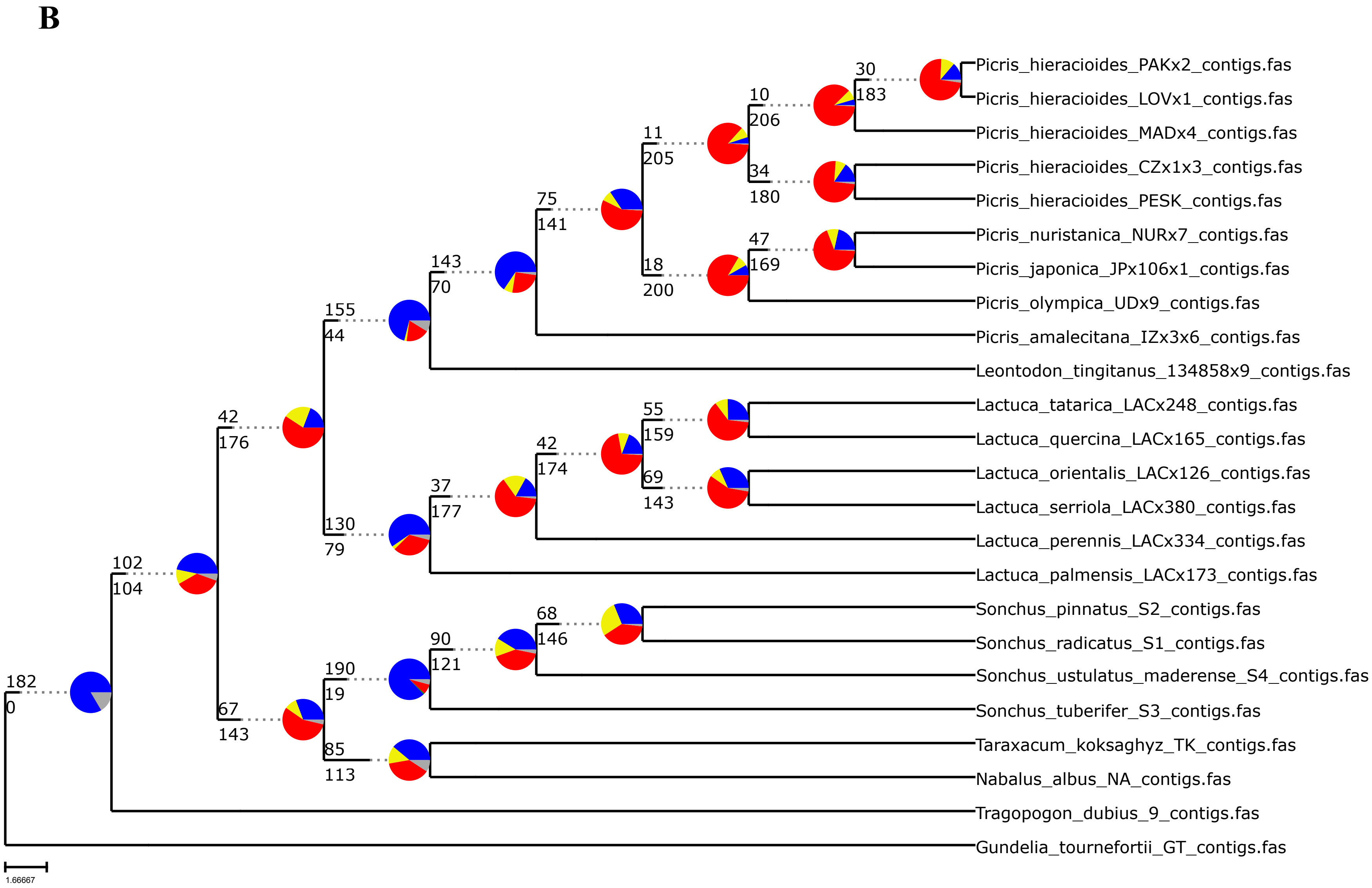
B
Picris_hieracioides_PAKx2_contigs.fas
Picris_hieracioides_LOVx1_contigs.fas
Picris_hieracioides_MADx4_contigs.fas
Picris_hieracioides_CZx1x3_contigs.fas
Picris_hieracioides_PESK_contigs.fas
Picris_nuristanica_NURx7_contigs.fas
Picris_japonica_JPx106x1_contigs.fas
Picris_olympica_UDx9_contigs.fas
Picris_amalecitana_IZx3x6_contigs.fas
Leontodon_tingitanus_134858x9_contigs.fas
Lactuca_tatarica_LACx248_contigs.fas
Lactuca_quercina_LACx165_contigs.fas
Lactuca_orientalis_LACx126_contigs.fas
Lactuca_serriola_LACx380_contigs.fas
Lactuca_perennis_LACx334_contigs.fas
Lactuca_palmensis_LACx173_contigs.fas
Sonchus_pinnatus_S2_contigs.fas
Sonchus_radicatus_S1_contigs.fas
Sonchus_ustulatus_maderense_S4_contigs.fas
Sonchus_tuberifer_S3_contigs.fas
Taraxacum_koksaghyz_TK_contigs.fas
Nabalus_albus_NA_contigs.fas
Tragopogon_dubius_9_contigs.fas
Gundelia_tournefortii_GT_contigs.fas
1.66667

C

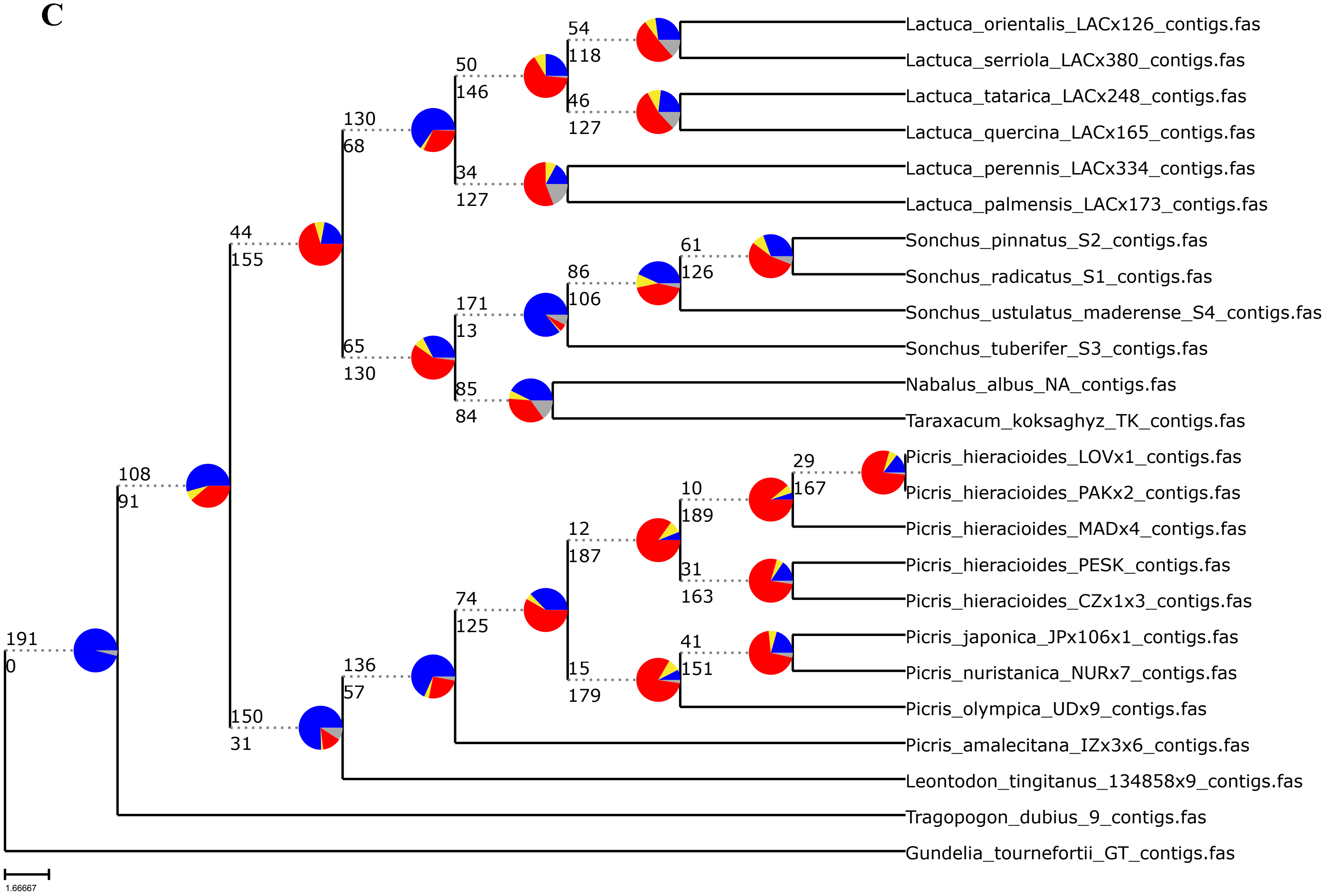
Lactuca_orientalis_LACx126_contigs.fas
Lactuca_serriola_LACx380_contigs.fas
Lactuca_tatarica_LACx248_contigs.fas
Lactuca_quercina_LACx165_contigs.fas
Lactuca_perennis_LACx334_contigs.fas
Lactuca_palmensis_LACx173_contigs.fas
Sonchus_pinnatus_S2_contigs.fas
Sonchus_radicatus_S1_contigs.fas
Sonchus_ustulatus_maderense_S4_contigs.fas
Sonchus_tuberifer_S3_contigs.fas
Nabalus_albus_NA_contigs.fas
Taraxacum_koksaghyz_TK_contigs.fas
Picris_hieracioides_LOVx1_contigs.fas
Picris_hieracioides_PAKx2_contigs.fas
Picris_hieracioides_MADx4_contigs.fas
Picris_hieracioides_PESK_contigs.fas
Picris_hieracioides_CZx1x3_contigs.fas
Picris_japonica_JPx106x1_contigs.fas
Picris_nuristanica_NURx7_contigs.fas
Picris_olympica_UDx9_contigs.fas
Picris_amalecitana_IZx3x6_contigs.fas
Leontodon_tingitanus_134858x9_contigs.fas
Tragopogon_dubius_9_contigs.fas
Gundelia_tournefortii_GT_contigs.fas
54
118
50
146
46
127
130
68
34
127
44
155
61
126
86
106
171
13
65
130
85
84
108
91
29
167
10
189
12
187
31
163
74
125
41
151
15
179
136
57
191
0
150
31
1.66667

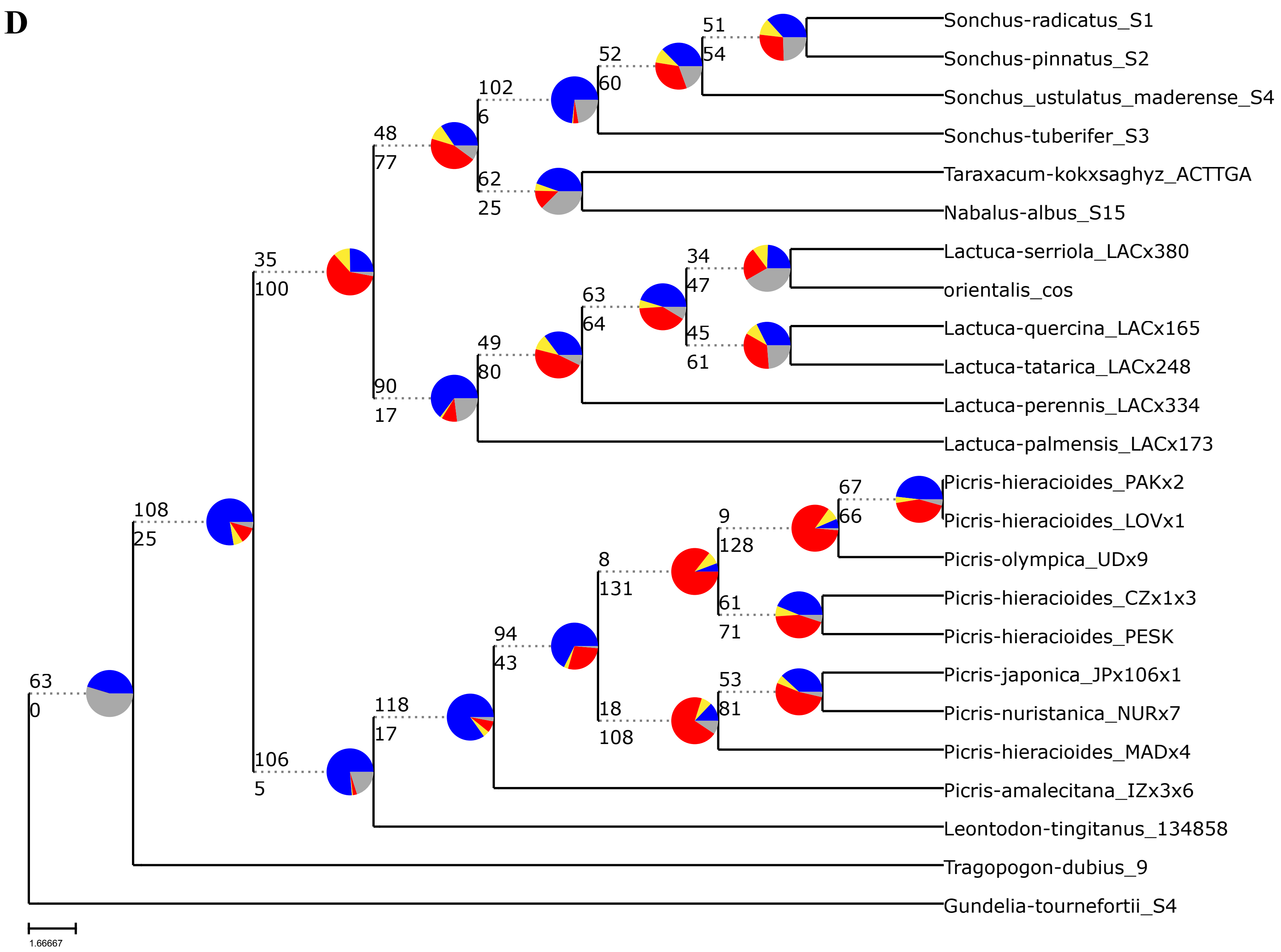
D
Sonchus-radicatus_S1
Sonchus-pinnatus_S2
Sonchus_ustulatus_maderense_S4
Sonchus-tuberifer_S3
Taraxacum-kokxsaghyz_ACTTGA
Nabalus-albus_S15
Lactuca-serriola_LACx380
orientalis_cos
Lactuca-quercina_LACx165
Lactuca-tatarica_LACx248
Lactuca-perennis_LACx334
Lactuca-palmensis_LACx173
Picris-hieracioides_PAKx2
Picris-hieracioides_LOVx1
Picris-olympica_UDx9
Picris-hieracioides_CZx1x3
Picris-hieracioides_PESK
Picris-japonica_JPx106x1
Picris-nuristanica_NURx7
Picris-hieracioides_MADx4
Picris-amalecitana_IZx3x6
Leontodon-tingitanus_134858
Tragopogon-dubius_9
Gundelia-tournefortii_S4
51
54
52
60
102
6
48
77
62
25
35
100
34
47
63
64
45
61
49
80
90
17
108
25
67
66
9
128
8
131
61
71
94
43
53
81
18
108
118
17
106
5
63
0
1.66667
