# Supplementary material for: An empirical assessment of a single family‐wide hybrid capture locus set at multiple evolutionary timescales in Asteraceae
Source: Appl Plant Sci. 2019 Oct 25;7(10):e11295. doi: 10.1002/aps3.11295 (PMC6814182; doi:10.1002/aps3.11295)
Supplement: Supplementary file 16 — APPENDIX S16. Boxplots and regression models (using Bayesian regression analyses) to explore the impact of sample type (herbarium and silica‐dried material) on number of reads mapped to targets and the off‐target plastome. [file APS3-7-e11295-s016.pdf]

Jones et al.—Applications in Plant Sciences 2019 7(10)—Data Supplement S16.
DOI

APPENDIX S16. Boxplots and regression models (using Bayesian regression analyses) to explore the impact of sample type (herbarium and silica-dried material) on number of reads mapped to targets and the off-target plastome. (A) Differences in number of reads mapped to targets depending on sample type (fresh, herbarium, and silica-dried; fresh samples were all processed with the oldest version of the probe kit). (B) Illustrates that when sample group A is taken into account, the positive effect of silica-dried material on numbers of reads mapped to targets is slightly higher than herbarium material. (C and D) Numbers (C) and percentages (D) of reads mapping to plastome for the three different leaf material types (fresh, herbarium, and silica-dried). (E) A brms regression model showing that herbarium samples recovered a higher proportion of the plastome (>4x coverage) compared to silica-dried material (percentage of the plastome recovered for fresh material was even higher than the latter two). The thick dark lines in boxplots (A, C, and D) indicate the median, the boxes correspond to the third (upper) and first quartile (lower), the dotted lines lead to the minimum and maximum values, and the circles correspond to outliers. In B and E, the circles indicate the estimated means and the vertical lines are error bars. See also Fig. 7. Script used in R for brms regression models can be found here: https://github.com/katy-e-jones/Asteraceae/blob/master/lab_modelling

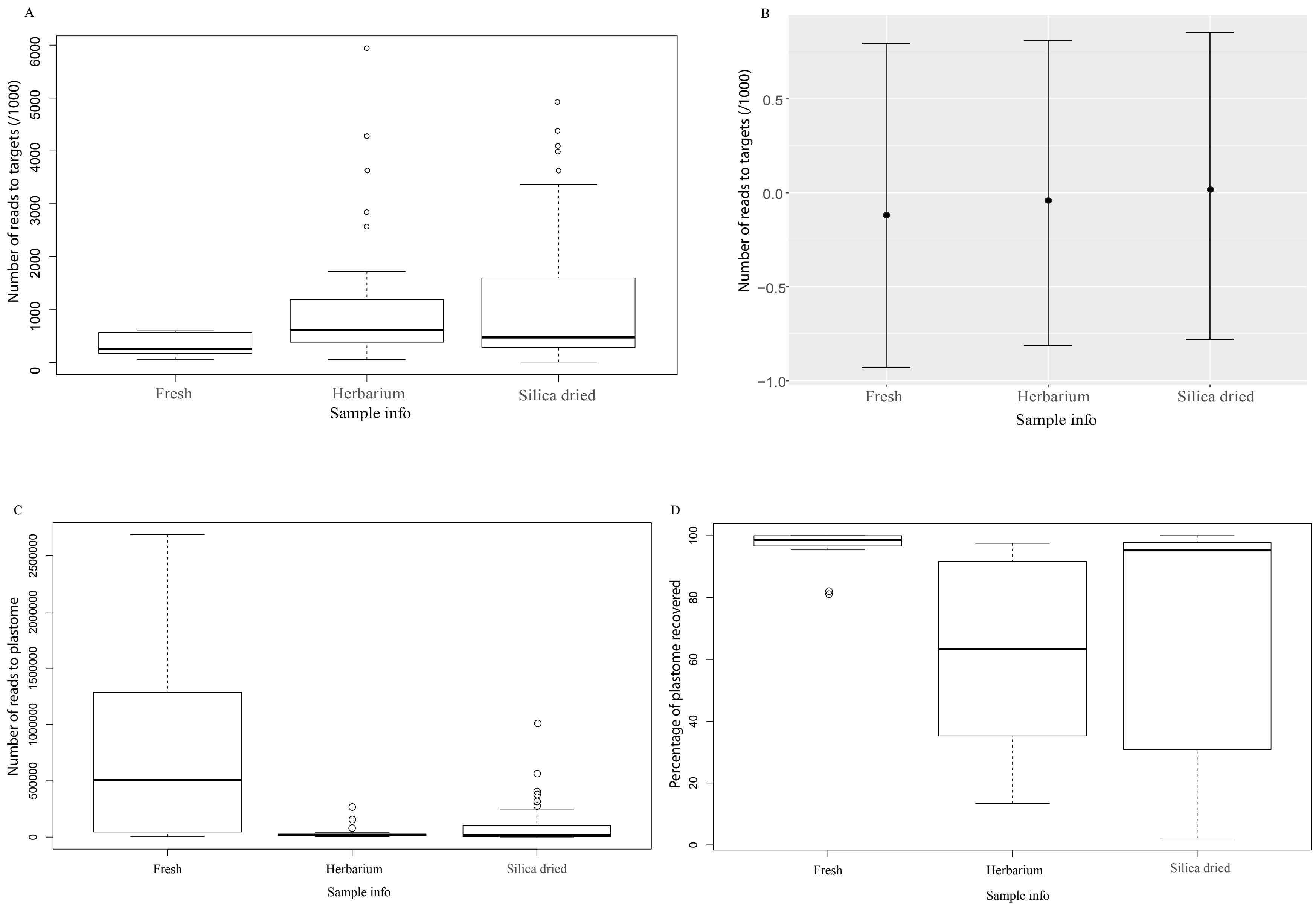

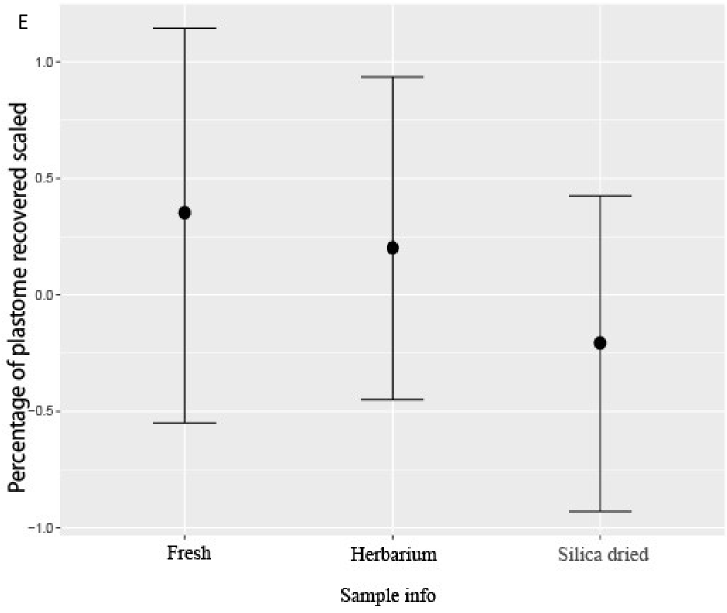
E
1.0
0.5
0.0
-0.5
-1.0
Percentage of plastome recovered scaled
Fresh
Herbarium
Silica dried
Sample info
